# Towards a comprehensive understanding of RNA deamination: synthesis and properties of xanthosine-modified RNA

**DOI:** 10.1093/nar/gkac477

**Published:** 2022-06-10

**Authors:** Stefan Mair, Kevin Erharter, Eva Renard, Karl Brillet, Melanie Brunner, Alexandra Lusser, Christoph Kreutz, Eric Ennifar, Ronald Micura

**Affiliations:** Institute of Organic Chemistry, Center for Molecular Biosciences Innsbruck, University of Innsbruck, Innsbruck 6020, Austria; Institute of Organic Chemistry, Center for Molecular Biosciences Innsbruck, University of Innsbruck, Innsbruck 6020, Austria; Architecture et Réactivité de l’ARN - CNRS UPR 9002, Institut de Biologie Moléculaire et Cellulaire, Université de Strasbourg, 67000 Strasbourg, France; Architecture et Réactivité de l’ARN - CNRS UPR 9002, Institut de Biologie Moléculaire et Cellulaire, Université de Strasbourg, 67000 Strasbourg, France; Institute of Molecular Biology, Biocenter, Medical University of Innsbruck, Innsbruck 6020, Austria; Institute of Molecular Biology, Biocenter, Medical University of Innsbruck, Innsbruck 6020, Austria; Institute of Organic Chemistry, Center for Molecular Biosciences Innsbruck, University of Innsbruck, Innsbruck 6020, Austria; Architecture et Réactivité de l’ARN - CNRS UPR 9002, Institut de Biologie Moléculaire et Cellulaire, Université de Strasbourg, 67000 Strasbourg, France; Institute of Organic Chemistry, Center for Molecular Biosciences Innsbruck, University of Innsbruck, Innsbruck 6020, Austria

## Abstract

Nucleobase deamination, such as A-to-I editing, represents an important posttranscriptional modification of RNA. When deamination affects guanosines, a xanthosine (X) containing RNA is generated. However, the biological significance and chemical consequences on RNA are poorly understood. We present a comprehensive study on the preparation and biophysical properties of X-modified RNA. Thermodynamic analyses revealed that base pairing strength is reduced to a level similar to that observed for a G•U replacement. Applying NMR spectroscopy and X-ray crystallography, we demonstrate that X can form distinct wobble geometries with uridine depending on the sequence context. In contrast, X pairing with cytidine occurs either through wobble geometry involving protonated C or in Watson–Crick-like arrangement. This indicates that the different pairing modes are of comparable stability separated by low energetic barriers for switching. Furthermore, we demonstrate that the flexible pairing properties directly affect the recognition of X-modified RNA by reverse transcription enzymes. Primer extension assays and PCR-based sequencing analysis reveal that X is preferentially read as G or A and that the ratio depends on the type of reverse transcriptase. Taken together, our results elucidate important properties of X-modified RNA paving the way for future studies on its biological significance.

## INTRODUCTION

Deamination of the nucleobases in DNA and RNA is a result of spontaneous hydrolysis, endogenous or environmental factors as well as deaminase enzymes (1,2). As a result (deoxy)cytidine, (deoxy)adenosine and (deoxy)guanosine are transformed into (deoxy)uridine, (deoxy)inosine, and (deoxy)xanthosine (dX, X) and (deoxy)oxanosines (dO, O). This can alter the genetically encoded information with significant consequences for cellular life. For example, in case of DNA, emerging deoxyinosine preferentially base pairs with cytosine instead of thymidine. These premutagenic events are counteracted by DNA repair enzymes specifically engaged in recognition and removal of deoxyinosines (3). In contrast, the deamination of adenosine to inosine in RNA is an essential cellular pathway involving specialized enzymes in a highly regulated manner to generate transcriptome diversity. Defects in A-to-I editing are associated with various human diseases including cancer, viral infections and neurological and psychiatric disorders (4,5). Enzymes catalyzing the A-to-I deaminase reaction are well characterized and known as a family of adenosine deaminases acting on double-stranded RNA (ADARs) (6,7). Likewise, albeit less frequent, C-to-U editing is an important mechanism for regulating genetic plasticity in mammalian cells. The best characterized example is the nuclear transcript encoding intestinal apolipoprotein B (apoB) (8), in which RNA editing changes a CAA to a UAA stop codon leading to a truncated protein which has important effects on lipoprotein metabolism (9). Interestingly, evidence for enzymatic editing of G-to-X in RNA has not yet been found. Nevertheless, at the nucleotide level, XMP plays a significant role in purine nucleotide metabolism and the corresponding purine salvage pathways that produce XMP from IMP for further processing into GMP (10–13). It was demonstrated that defects in purine nucleotide metabolism lead to substantial incorporation of xanthine and hypoxanthine into DNA and RNA which may also provide a mechanistic basis for the pathophysiology of human inborn errors of purine nucleotide metabolism (14).

It is surprising that xanthosine containing RNA is underexplored; hardly anything is known about its biophysical properties. To the best of our knowledge, neither efficient approaches for the chemical solid-phase synthesis of xanthosine modified RNA nor thermodynamic data on xanthosine base pairing in RNA are currently available. The same applies for the 3D architecture of xanthosine RNA; not a single crystallographic study that would allow precise insights into xanthosine base pair geometries and potential chemical and biochemical consequences is found in the literature. Only one NMR spectroscopic study of xanthosine containing RNA prepared by means of transcription using T7 polymerase has been directed towards this aim very recently (15). The urgent need for thorough biophysical characterization of xanthosine RNA arises on one hand from a biomedical perspective to better understand nucleic acid damage caused by deamination through spontaneous hydrolysis (being a slow process) or nitrosative chemistry (being a fast process), and from nucleic acid editing through regulated deamination by specific enzymes (yet to be discovered for G-to-X transformations) (1–3,14,55,56). In addition, knowledge about the properties of nucleotide deamination products becomes important also in the light of recent methodological developments of deep sequencing approaches. For instance, NO-seq is a novel method to map m^6^A modification sites in RNA by exploiting the fact that nitrous acid deaminates all exocyclic amino groups present in nucleobases and thus significantly changes sequence information but leaves m^6^A intact (16,17). Since NO also induces G-to-X transformations, it is important to understand the behavior of X in subsequent amplification and sequencing reactions to increase the reliability of mapping and m^6^A calling algorithms.

Here, we set out to accomplish a thorough chemical and biophysical analysis of xanthosine containing RNA, along with the synthesis of an appropriately protected xanthosine building block for RNA solid-phase synthesis beforehand. We describe the impact of xanthosine on RNA properties. Based on UV-spectroscopic melting experiments, a detailed thermodynamic analysis of duplex and hairpin stabilities is provided and the effects on base pairing are discussed in the light of the sequence context. Furthermore, solution NMR spectroscopy sheds light on base pair geometry. In addition, we have solved the X-ray structure of xanthosine containing RNAs at atomic resolution to disclose crucial structural features, such as ribose puckers, hydrogen-bonding networks, metal ion interactions, and hydration patterns of the xanthosine, and to correlate them to base pairing properties. Finally, we report on reverse transcription assays and RNA sequencing analysis revealing that X in RNA is read as G and A in distinct ratio depending on the type of reverse transcriptase used.

## MATERIALS AND METHODS

### Synthesis and characterization of organic compounds

Reagents were purchased in the highest available quality from commercial suppliers (Merck/Sigma-Aldrich, ABCR, VWR, ChemGenes, CarboSynth) and used without further purification. All reactions were carried out under argon atmosphere, unless otherwise noted. Analytical thin-layer chromatography (TLC) was performed on Macherey-Nagel Polygram® SIL G/UV_254_ plates. Silica gel 60 (mesh size 0.04–0.063 mm) for column chromatography was purchased from Macherey-Nagel. The procedures for chemical synthesis of xanthosine phosphoramidite **9** and the characterization data are available in the Supporting Information (phosphoramidite **9**: nine steps, nine chromatographic purifications, 21% overall yield; total amount synthesized: 2.5 g. ^1^H, ^13^C and ^31^P NMR spectra were recorded on a Bruker Ultrashield^TM^ 400 Plus spectrometer. Chemical shifts (δ) are reported relative to tetramethylsilane (TMS), referenced to the residual solvent signal (DMSO-d_6_: 2.50 ppm for ^1^H and 39.52 ppm for ^13^C NMR spectra; CDCl_3_: 7.26 ppm for ^1^H and 77.16 ppm for ^13^C NMR spectra). The following abbreviations were used to denote multiplicities: s = singulet, d = doublet, t = triplet, q = quadruplet, m = multiplet, b = broad. Signal assignments are based on ^1^H–^1^H-COSY, ^1^H–^13^C-HSQC and ^1^H–^13^C-HMBC experiments. High resolution mass spectra were recorded in positive ion mode on a Thermo Scientific Q Exactive Orbitrap, ionized via electrospray at 3.7 kV spray voltage.

### RNA solid-phase synthesis

Standard phosphoramidite chemistry was applied for RNA strand elongation and the incorporation of xanthosine building block **9** (>98% coupling yield). 2′-*O*-TOM and acetyl protected nucleoside phosphoramidite building blocks and 2′-*O*-Tbs 1000 Å CPG solid support (>15nt) were purchased from ChemGenes, Primer support^TM^ 5G (<15nt) was purchased from GE Healthcare. ^15^N-labeled nucleoside phosphoramidites were provided by INNotope (https://www.innotope.at). All oligonucleotides were synthesized on an ABI 392 nucleic acid synthesizer following standard methods: detritylation (90 sec) with dichloroacetic acid/1,2-dichloroethane (4/96); coupling (5.0 min) with phosphoramidites/acetonitrile (100 mM, 200 μl) and benzylthiotetrazole/acetonitrile (300 mM, 500 μl); capping (2 × 25 sec) with Cap A/Cap B (1/1) for unmodified and xanthosine modified RNA, Cap A: 4-(dimethylamino)pyridine/acetonitrile (500 mM), Cap B: acetic anhydride/*sym*-collidine/acetonitrile (2/3/5); oxidation (60 sec) with iodine (20 mM) in tetrahydrofuran/pyridine/H_2_O (35/10/5). Solutions of phosphoramidites, tetrazole and Cap were dried over activated molecular sieves (3 Å) overnight.

### Deprotection, purification and quantification of unmodified and xanthosine modified RNA

For deprotection of unmodified and xanthosine modified RNA, the solid support was mixed with aqueous methylamine (40%, 0.50 ml) and aqueous ammonia (28%, 0.50 ml) for 15 to 25 minutes at 65°C. The supernatant was removed and the solid support was washed twice with H_2_O/ethanol (1.0 ml; 1/1) and once with tetrahydrofuran. Combined supernatant and washings were evaporated to dryness and the residue was dissolved in a solution of tetrabutylammonium fluoride in tetrahydrofuran (1.0 M, 1.0 ml) and incubated for 14 h at 37°C for removal of 2′-*O*-silyl protecting groups. The reaction was quenched by addition of triethylammonium acetate/H_2_O (1.0 M, 1.5 ml, pH 7.4). Tetrahydrofuran was removed under reduced pressure and the sample was desalted with size-exclusion column chromatography (GE Healthcare, HiPrep™ 26/10 Desalting; Sephadex G25) eluting with H_2_O; collected fractions were evaporated and the RNA dissolved in H_2_O (1 ml). The crude RNA was purified by anion exchange chromatography (GE Healthcare Äkta Basic HPLC System) on a semipreparative Dionex DNAPac® PA-100 column (9 mm x 250 mm) at 80°C with a flow rate of 2 ml/min (eluent A: 20 mM NaClO_4_, 25 mM Tris·HCl, pH 8.0, 20 v/v % acetonitrile; eluent B: 600 mM NaClO_4_, 25 mM Tris·HCl, pH 8.0, 20% v/v acetonitrile). Fractions containing RNA were evaporated an the residue redissolved in 0.1 M triethylammonium bicarbonate solution (10 to 20 ml), loaded on a C18 SepPak Plus^®^ cartridge (Waters/Millipore), washed with H_2_O, and then eluted with acetonitrile/H_2_O (1/1). Crude and purified RNA were analyzed by anion exchange chromatography (GE Healthcare Äkta Basic HPLC System) on a Dionex DNAPac^®^ PA-100 column (4 mm × 250 mm) at 80°C with a flow rate of 1 ml/min. For RNA up to 15 nucleotides in length, a gradient of 0–44% B in 30 min was applied; for longer RNA a gradient of 0–60% B was applied; eluent A: 20 mM NaClO_4_, 25 mM Tris·HCl, pH 8.0, 20% v/v acetonitrile; eluent B: 600 mM NaClO_4_, 25 mM Tris·HCl, pH 8.0, 20% v/v acetonitrile. HPLC traces were recorded at UV absorption by 260 nm. RNA quantification was performed on an Implen P300 Nanophotometer.

### Mass spectrometry of oligoribonucleotides

RNA samples (∼200 pmol in ∼3 μl) were diluted with aqueous soulution of ethylenediaminetetraacetic acid disodium salt dihydrate (Na_2_H_2_EDTA) (40 mM, 15 μl). Water was added to obtain a total volume of 30 μl. The sample injected onto a C18 XBridge column (2.5 μm, 2.1 mm × 50 mm) at a flow rate of 0.1 ml/min and eluted using a gradient 0 to 100% B gradient at 30°C (eluent A: 8.6 mM triethylamine, 100 mM 1,1,1,3,3,3-hexafluoroisopropanol in H_2_O; eluent B: methanol). RNA was detected by a Finnigan LCQ Advantage Max electrospray ionization mass spectrometer with 4.0 kV spray voltage in negative mode.

### Melting curve measurements of oligoribonucleotides

RNA samples were lyophilized as triethylammonium or sodium salts, dissolved in 800 or 330 μl buffer (10 mM Na_2_HPO_4_ (pH 7.0) containing 150 mM NaCl) and transferred into UV permeable high precision cells made of quartz SUPRASIL^®^ with a light path of 10 mm or 1 mm. UV melting profiles were recorded at 250 and 260 nm on a Varian Cary-100 spectrophotometer equipped with a multiple cell holder and a peltier temperature control device. Each RNA was measured at five different concentrations (between ∼1 and ∼100 μM) and with at least four ramps (heating-cooling-heating-cooling; 1°C min^–1^ heating/cooling rate). *T*_m_ values were determined by calculating the first derivative, usually from data of the fourth ramp (cooling). Thermodynamic parameters were determined according to reference (18,19). The error limits for Δ*G*, Δ*H* and Δ*S* reflect the standard deviation of at least three independent measurements for a confidence interval of 95%.

For the pH dependent UV-melting curve experiments the following buffers were used: pH 5.0: 10 mM Na_2_HPO_4_ containing 150 mM NaCl; pH 5.99: 10 mM Na_2_HPO_4_ containing 150 mM NaCl.

### NMR experiments of oligoribonucleotide

RNA samples were lyophilized as triethylammonium or sodium salts, dissolved in 500 μl NMR buffer (15 mM sodium phosphate, 25 mM NaCl, 3 mM NaN_3_, in H_2_O/D_2_O 9:1, pH 6.5) and transferred into 5 mm NMR tubes. Sample concentrations varied between 0.1 and 2.0 mM and experiments were run at 298 K unless otherwise stated. All NMR experiments were conducted on a Bruker 600 MHz Avance II + NMR or a 700 MHz Avance Neo NM both equipped with a Prodigy TCI probe. Assignments of imino proton signals were achieved by a combination of ^1^H–^1^H jump and return NOESY and ^1^H–^15^N sofast HSQC experiments.

For the pH dependent NMR experiments the following buffers were used: pH 4.2: 15 mM sodium phosphate, 25 mM NaCl, in H_2_O/D_2_O 9:1; pH 8.1: 15 mM sodium phosphate, 25 mM NaCl, in H_2_O/D_2_O 9:1.

### Crystallization and structure solution of xanthosine modified RNAs

Three oligoribonucleotide sequences have been used for crystallization: a 27 nt RNA corresponding to the sequence of *E. coli* 23 S rRNA sarcin-ricin loop (SRL) (20,21) and containing xanthosine at position 2648 (Xan2648-SRL), a dodecamer RNA similar to a previously-published oligoribunucleotide (22) but containing a xanthosine instead of an adenosine at position 5 (Xan-12-mer), and a 14-mer RNA similar to a previously published oligoribonucleotide (23) but containing a xanthosine instead of a guanosine at position 10 (Xan-14-mer). The Xan2648-SRL and Xan-14-mer RNAs were dissolved at a concentration of ∼350 μM in a buffer made of Tris-HCl (10 mM), Na_2_H_2_EDTA (1 mM), pH 8.0. Both RNA samples were then heated to 55°C and cooled down to 10°C using a temperature-controlled device equipped with a Peltier element. For Xan2648-SRL, only one unique cubic-shaped crystal could be obtained. It grew after one month at 20°C using vapor diffusion method by mixing one volume of RNA sample with one volume of a crystallization buffer made of ammonium sulfate (2.5 M), magnesium acetate (10 mM), and 2-(*N*-morpholino)ethanesulfonic acid (MES) (50 mM), pH 5.6 (the other drops made in identical conditions led to spherullites). Prior data collection, the crystal was cryoprotected for about 5 min in a reservoir solution containing 15% of glycerol and 3.0 M of ammonium sulfate, flash-frozen in liquid ethane and then transferred into liquid nitrogen. The Xan-14-mer RNA was crystallized at 20°C by mixing one volume of RNA sample with one volume of a solution made with 25 mM MgSO_4_, 50 mM Tris-HCl pH 8.5, 1.8 M ammonium sulfate. Crystals were cryoprotected for about 5 min by adding ∼5 volumes of a solution made with 2.5 M ammonium sulfate and 20% glycerol, then flash frozen in liquid ethane. The Xan-12-mer RNA was dissolved in water at a concentration of 1 mM, heated at 80°C for 10 min and cooled at 20°C at a 1°C min^–1^ rate. Crystals were grown at 20°C by mixing 2 μl of RNA sample with 2 μl of a solution made with 10% (v/v) 2-methyl-2,4-pentanediol (MPD), 40 mM sodium cacodylate pH 7.0, 12 mM spermine, 80 mM NaCl, 30 mM MgCl_2_, against a reservoir made by 35% MPD. Crystals were frozen in liquid ethane without any further cryoprotection. X-ray diffraction data at atomic resolution for all three crystals was performed on the X06DA beamline at the SLS synchrotron, Villigen, Switzerland. Processing of the data was done with the XDS Package (24) and structures were solved by molecular replacement with MOLREP (25) using PDB ID 3DVZ (SRL), 2Q1R (12-mer) and 433D (14-mer) as search models. Structures were refined with the PHENIX package (26) and models were built using Coot (27). Coordinates have been deposited with the PDB database (PDB ID 7QSH for Xan2648- SRL, 7QTN for Xan-14-mer and 7QUA for Xan-12-mer).

### Primer extension analysis

The 37 nt RNA 5′-AUUCCUCXUCAUCCAUACAGACAGAACUAACGAUUCG (10 μl; 1 μM) and 4 μl of Alexa Fluor 647 5′-end labelled DNA primer (2 pmol/μl; IDT; 5′-/5Alexa647N/CGAATCGTTAGTTCTGTC-3′) were annealed for 5 min at 65°C, then incubated at 35°C for 5 min and cooled on ice for 1 min (performed in an Eppendorf Mastercycler personal). Then, 8 μl of a ‘primer extension mix’ containing 4 μl of 5× SS IV RT buffer (provided by supplier), 1 μl of 0.1 M 1,4-dithiothreitol (DTT) solution, 1 μl of 5 mM dNTPs mixture (1.25 mM for each dNTP), 2 μl of dimethyl sulfoxide (DMSO), and 0.4 μl of SuperScript IV reverse transcriptase (200 U/μl; Invitrogen) were added and the reaction mixtures incubated at 55°C for 10 min (28,29). The primer extension reaction was stopped by addition of 1 μl of 4 M NaOH solution, the mixture was incubated at 90°C for 5 min, and then cooled on ice. The Alexa Fluor 647 labelled cDNA strands were precipitated by adding 90 μl of precipitation solution (650 μl water, 150 μl 1 M NaOAc pH 5.2, 10 μl of 20 mg/ml glycogen) and 250 μl of cold ethanol and stored for 30 min at –20°C. After centrifugation for 30 min at 4°C at 11 740 rpm, the supernatant was removed and the pellets dried under reduced pressure. The pellets were resuspended in 8 μl of gel loading buffer (97% formamide, 10 mM EDTA), loaded on 10% polyacrylamide gels with 7 M urea and run for approximately 120 min at 35 W. Sequencing ladders were produced by adding 2 μl of 5 mM ddNTPs to the RNA samples prior to addition of the ‘primer extension mix’. The extension products were analyzed by scanning the gel at 635 nm with a Typhoon FLA 9500 instrument (GE Healthcare).

### PCR-mediated analysis of X-containing RNA

#### Ligation of template

RNA containing two xanthosines at positions 33 and 61 (5′-AAUGUAAAACGACGGCCAGGCUUAAGCCCUAA-X–CGUUGAUAGUUAGAUUCCUCAUCAUCC–X–UACAGACAGAACUAACGAUUCG-3′) was prepared by splint ligation using T4 DNA ligase (Fermentas). Both RNAs fragments (2 nmol each; 5′-AAUGUAAAACGACGGCCAGGCUUAAGCCCUAA-X–CGUUGAUAGUUAG and 5′-p-AUUCCUCAUCAUCC–X–UACAGACAGAACUAACGAUUCG) and the DNA splint oligonucleotide (2 nmol; 5′-ATGATGAGGAATCTAACTATCAAC) were dissolved in water (140 μl), heated to 90°C for 2 min, and cooled to room temperature. Then, 10× ligation buffer (20 μl; 400 mM Tris–HCl, 100 mM MgCl_2_, 100 mM DTT, 5 mM ATP (pH 7.8 at 25°C)), poly(ethylene glycol) (50% PEG 4000 solution) (20 μl) and T4 DNA ligase (20 μl, 5 U/μl) were added to obtain a total volume of 200 μl. The mixture was incubated for 5 h at 37°C. The reaction was stopped by a phenol/chloroform extraction (twice) and extracted three times with chloroform to remove remaining phenol from the reaction. Analysis of the reaction and purification was performed via anion exchange chromatography on analytic columns (for conditions see above section ‘Deprotection, purification and quantification of unmodified and xanthosine modified RNA’.

#### PCR analysis

RNA was reverse transcribed with primer Xant_long (5′-GATCCGAATCGTTAGTTCTGTC) using either the GoScript Reverse Transcription System (Promega) or SuperScript IV (ThermoFisher) according to the manufacturer's instructions. The resulting cDNA was amplified by PCR using primers Xant_fw (5′-AAUGUAAAACGACGGCCAG) and Xant_rev (5′-CGAATCGTTAGTTCTGTC) and GoTaq polymerase (Promega). PCR products were purified from agarose gels, ligated to pGEM-T vector (Promega) and used to transform bacteria. Plasmid DNA was isolated from single colonies and subjected to Sanger sequencing.

## RESULTS AND DISCUSSION

### Synthesis of xanthine building block

For the solid–phase synthesis of xanthosine modified RNA, appropriate phosphoramidite building blocks are needed. While for 2′-deoxyxanthosine phosphoramidites several approaches have been reported in the literature (30–33), for xanthosine only a single study can be found that describes a nucleobase unprotected 2′-*O*-*tert*-butyldimethylsilyl (TBS) phosphoramidite. This building block was used in an early mutational study from 1993 to evaluate the activity of active site nucleosides in the hammerhead ribozyme (34). The problem with unprotected nucleobase xanthosine phosphoramidites, however, is very low coupling yield (34), and more alarming, branching at the O2 of the nucleobase leading to cross-coupled oligonucleotides (35).

**Scheme 1.**
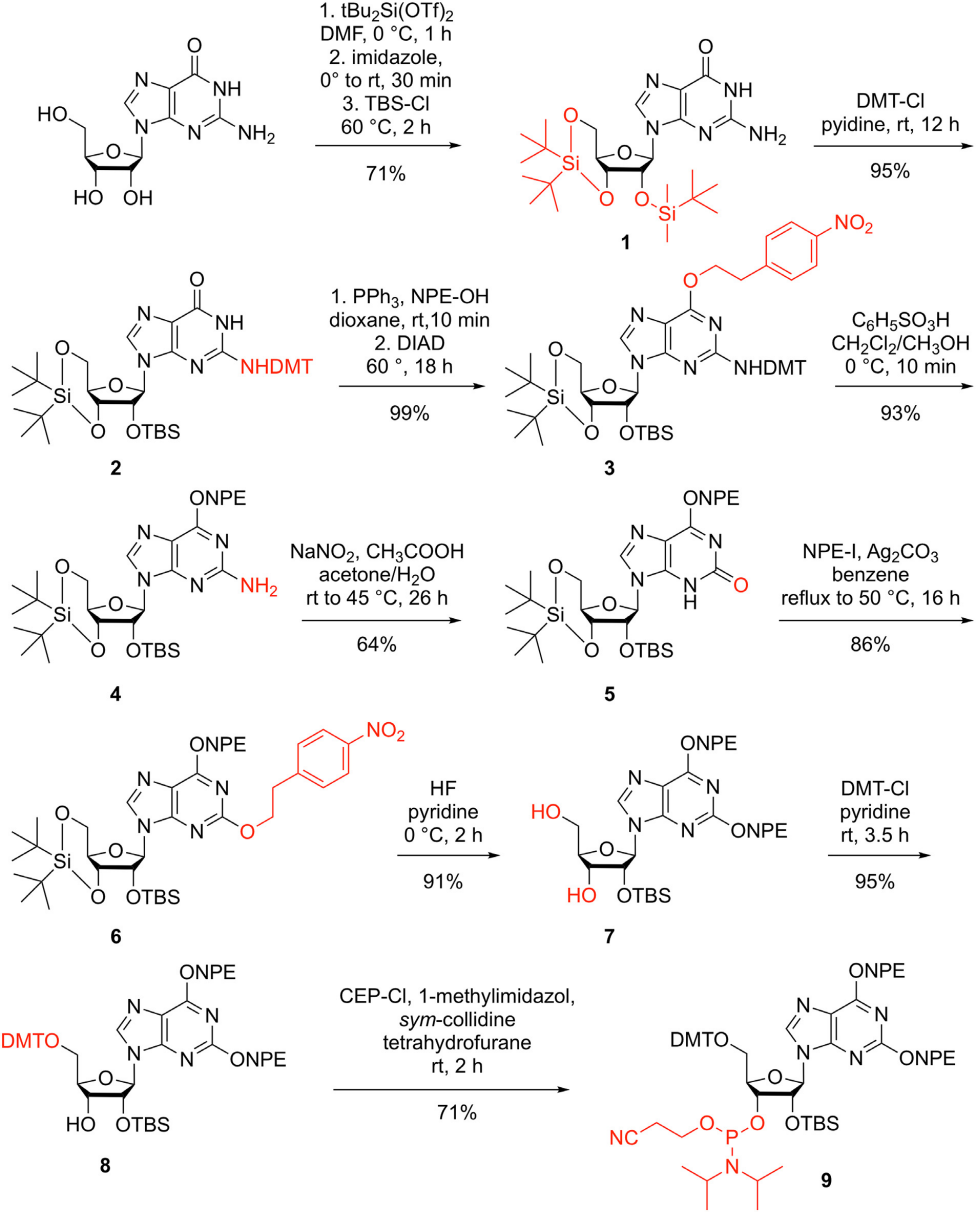
Synthesis of the novel xanthosine building block **9**. *tert*-butyldimethylsilyl chloride (TBS), 4,4′-dimethoxytriphenylmethyl (DMT), 4-(*N*,*N*-dimethylamino)pyridine (DMAP), 2-(4-nitrophenyl)ethyl (NPE), chloro-2-cyanoethyl-*N*,*N*-diisopropylphosphoroamidite (CEP).

Our novel synthetic route to obtain a powerful nucleobase protected xanthosine phosphoramidite started from guanosine by applying Beigelman protection of the 5′ and 3′ hydroxyl groups to regioselectively install the 2′-*O*-TBDMS group providing compound **1** in high yields (Scheme 1). Then, the exocyclic NH_2_ of guanine was protected with a dimethoxytrityl moiety (DMT) and the resulting derivative **2** was treated under Mitsunobu conditions to introduce the *O*^6^-(4-nitrophenyl)-2-ethyl (NPE) protecting group which gave compound **3**. The selective cleavage of the DMT under acidic conditions provided the free NH_2_ functionality of compound **4** that was further transformed into the 2-oxo group of compound **5** via the diazonium intermediate using aqueous sodium nitrate in acetone and acetic acid. Then, alkylation of O2 using 1-(2-iodoethyl)-4-nitrobenzene (NPE-I) and silver carbonate proceeded smoothly with high selectivity to yield compound **6**. Selective removal of the 3′,5′-*O*-di-*tert*-butyl silylether was accomplished with hydrogen fluoride (HF) in pyridine and gave the diol **7** in high yield, leaving the 2′-*O*-tert-butylsilyl group unaffected. Nucleoside **7** was then transformed into the dimethoxytritylated compound **8** using 4,4′-dimethoxytriphenylmethyl chloride in pyridine without any additional base to avoid migration of the 2′-*O*-tert-butylsilyl group. Finally, phosphitylation was executed with 2-cyanoethyl-*N*,*N*-diisopropylchlorophosphoramidite in the presence of 1-methylimidazole and *sym*-collidine. Starting from guanosine, our route provides phosphoramidite **9** with 21% overall yield in nine steps and with nine chromatographic purifications; in total, 2.1 g of **9** was obtained in the course of this study.

### Synthesis of xanthosine containing RNA

**Figure 1.**
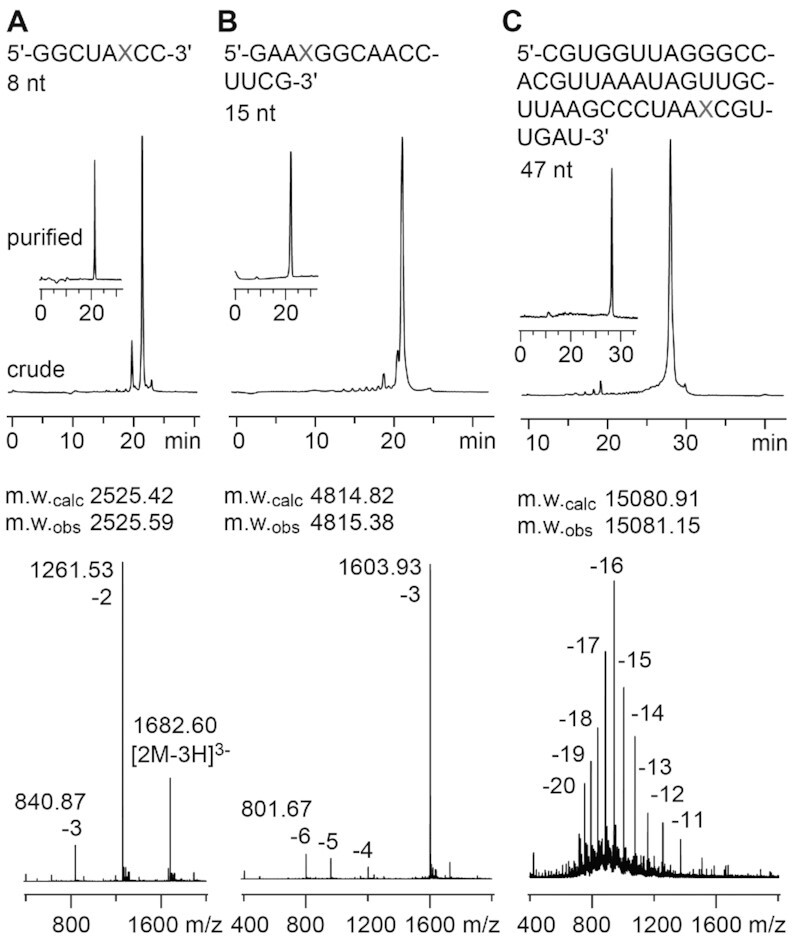
Characterization of X-modified RNA. (**A**) Anion-exchange HPLC traces of 8 nt RNA (top) and LC–ESI iontrap mass spectrum (bottom). (**B**) Same as (A) but for 15 nt RNA; (**C**) Same as (A) but for 47 nt RNA. HPLC conditions: Dionex DNAPac column (4 × 250 mm), 80°C, 1 ml min^–1^, 0–40% buffer B (for 8 nt RNA) in buffer A within 30 min; 0–60% buffer B (for 47 nt RNA) in buffer A within 30 min; buffer A: Tris–HCl (25 mM), acetonitrile (20% v/v), NaClO_4_ (20 mM), pH 8.0; buffer B: Tris–HCl (25 mM), acetonitrile (20 v/v %), NaClO_4_ (600 mM), pH 8.0. See the experimental for LC–ESI MS conditions.

The incorporation of building block **9** into oligoribonucleotides proceeds with excellent coupling yields using standard RNA solid-phase synthesis protocols. Importantly, deprotection of the NPE groups also proceeds under standard conditions. Figure 1 exemplarily illustrates the synthesis of 8, 15 and 47 nt long RNA oligonucleotides with a single xanthosine modification (for a complete list of synthesized oligos see Supporting Table S1). In short, the xanthosine phosphoramidite building block **9** was applied in combination with standard *N*-acetylated 2′-*O*-[(triisopropylsilyl)oxy]methyl (TOM) phosphoramidites and the oligomers were assembled on controlled pore glass (CPG) supports (36,37). Cleavage from the solid support and deprotecting of the base labile groups were accomplished by treatment with methylamine/ammonia in water (1:1 mixture of 40% aqueous methylamine and 28% aqueous ammonia (AMA) for 15 to 25 min at 65°C). Subsequently, deprotection of the the *O*^6^- and *O*^2^-NPE groups, and the 2′-*O*-silyl groups was carried out with tetra-*n*-butylammonium fluoride trihydrate in tetrahydrofuran for 14 hours at 37°C. The reaction was quenched by the addition of triethylammonium acetate buffer at pH 7.4. Salts were removed by size-exclusion chromatography. Analysis by anion exchange chromatography under strong denaturing conditions usually gave a major peak for the desired RNA which was further purified by anion exchange chromatography on a semipreparative column. The molecular weights of the purified RNAs were confirmed by LC–ESI-MS (Supporting Table S1). Of note, we had no indication for depurination at the RNA xanthosine lesions at physiological and lower pH values (down to pH 4.5). The latter were found problematic for the corresponding 2′-deoxyxanthosine lesions in DNA (57).

### Thermodynamic base pairing properties of xanthosine containing RNA

**Figure 2.**
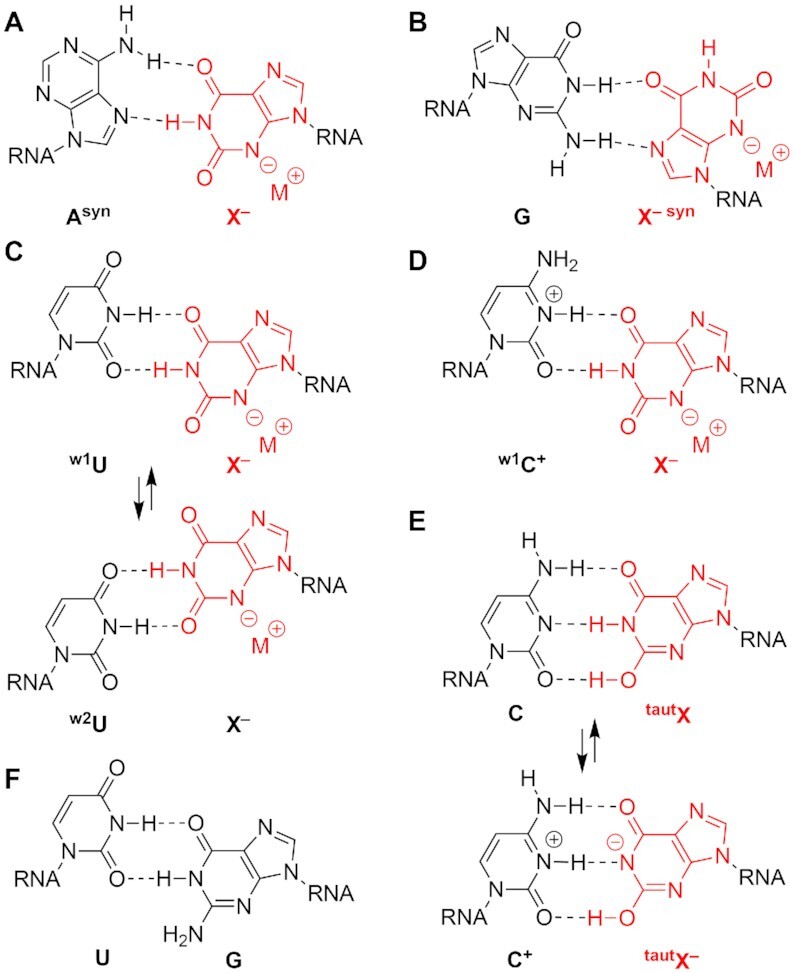
Putative interactions for xanthosine containing base pairs in RNA. (**A**) Xanthosine-adenosine; (**B**) xanthosine-guaninosine; (**C**) xanthosine-uridine; (**D**) bidendate xanthosine-cytidine (wobble-like); (**E**) tridendate xanthosine-cytidine (Watson–Crick like); (**F**) standard G–U wobble pair for reason of comparison.

Hardly anything is known about the impact of xanthosine on RNA base pairing. We therefore set out to determine the pairing stabilities of X-modified RNA double helices with regard to mismatch formation of X and the standard nucleotides A, G, C and U, respectively (Figure 2A–D). Potential geometries of X with A or G include *syn* conformations of A and X (Figure 2A, B). Pairing of X to U appears possible in two distinct wobble geometries (Figure 2C) while base pairing of X to C should favor only one wobble geometry requiring a protonated C to interact in bidentate fashion with X (Figure 2D) resembling the shape of a standard U–G wooble pair (Figure 2F). For pairing of X with C, we also have to consider Watson–Crick shape-complementary base pairing as shown in Figure 2E. The putative tridendate pairing involves a tautomeric form of xanthine (X^taut^) (Figure 2E, top); further, upon proton shuffling from X(N1-H) to C(N3) charge separation X(N1^–^) to C(N3^+^-H) is conceivable (Figure 2E, bottom). We furthermore point out the p*K*_a_^N3-H^ value of 5.2 was reported for xanthosine (38), and therefore in buffer solutions at physiological pH, xanthosine bases in RNA are deprotonated.

**Figure 3.**
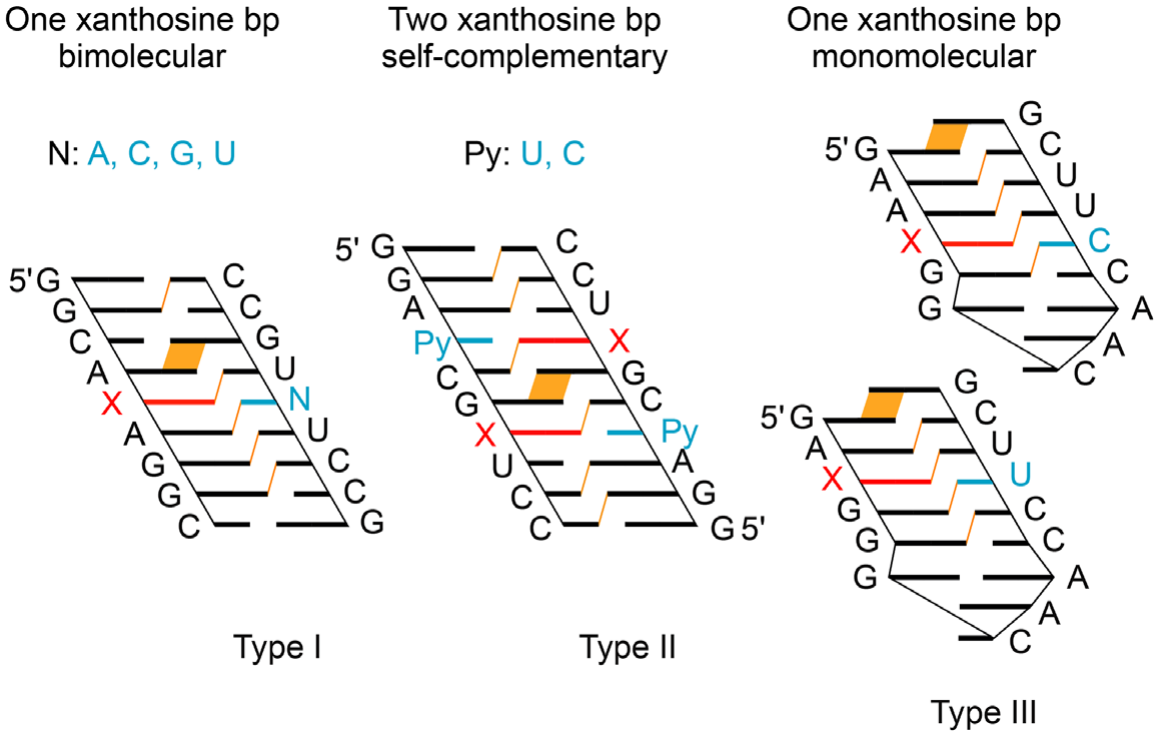
Sequence design for thermodynamic analysis of base pairing of X-modified RNAs. Cartoon presentation to highlight inter-strand stacking interactions (in orange).

The sequence design of the RNAs investigated here is illustrated in Figure 3. The first motif constitutes an asymmetric bimolecular duplex of nine base pairs with a single xanthosine modification in the center (Type I). The second motif represents a palindromic RNA of 10 bp and inter-strand purine–purine stacking with two xanthosine pairs separated by two standard base pairs (Type II). The third motif forms a hairpin with an extra-stable GNRA loop (GCAA), a 5 bp stem and a 3′-dangling guanosine to reduce fraying of the terminal base pair, with xanthosine base-paired to uridine and cytosine respectively (Type III). We point out that the type II sequence design should be particularly sensitive to the thermodynamic impact arising from a modification because only two and three regular Watson–Crick base pairs can form next to the X pairs: we stress that nucleation of a bimolecular double helix of oligonucleotides becomes thermodynamically favorable only when at least (three to) four continuous Watson–Crick base pairs can form (39,40). Therefore, these palindromic RNAs can respond to a nucleobase modification by significant changes (decease or increase) in thermal stabilities (*T*_m_) and the corresponding thermodynamic parameters (Δ*G*, Δ*H*, Δ*S*).

Table 1 summarizes the thermal and thermodynamic data we obtained for the three RNA systems by UV-spectroscopic melting profile measurements (for melting profiles see the Supporting Figures S1 to S16, for and extended table see Supporting Table S2). The native type I RNAs melt at 67.7°C (G5–C5) and at 59.5°C (A5–U5). Replacement of the purine-5 by xanthosine causes destabilization, depending on the nature of its pairing partner, being largest for G and A opposite of X (–22.0/–22.6°C relative to G5–C5, and –13.8/–14.4°C relative to A5–U5, **Ic/If** and **Ic/Ig**). Also for C opposite of X, destabilization is significant and of comparable extent (–20.9°C relative to G5–C5, and –12.7°C relative to A5–U5; **Ic/Id**). Only when U is located opposite of X, destabilization is less (–12.6°C relative to G5–C5, and –4.4°C relative to A5–U5; **Ic/e**).

**Table 1.** Thermodynamic data of xanthosine modified RNA^a^

No.	RNA sequences 5′ to 3′	*T*_m_ (°C)^b^	Δ*T*_m_ (°C)	Δ*G*°298 (kcal mol^–1^)^c^	Δ*H*°298 (kcal mol^–1^)^c^	Δ*S*° (cal mol^–1^ K^–1^)^c^
**Ia/Id**	GGCAGAGGC/GCCUCUGCC	**67.7 ± 0.2**	–	–17.2 ± 0.3	–84.0 ± 2.0	–224 ± 6
**Ib/Ie**	GGCAAAGGC/GCCUUUGCC	**59.5 ± 0.1**	–8.2	–15.4 ± 0.1	–84.0 ± 0.7	–230 ± 2
**Ic/If**	GGCAXAGGC/GCCUGUGCC	**45.7 ± 0.4**	–22.0/–13.8	–11.4 ± 0.1	–71.9 ± 1.0	–203 ± 4
**Ic/Ig**	GGCAXAGGC/GCCUAUGCC	**45.1 ± 0.3**	–22.6/–14.4	–11.3 ± 0.3	–72.4 ± 3.6	–205 ± 11
**Ic/Id**	GGCAXAGGC/GCCUCUGCC	**46.8 ± 0.2**	–20.9/–12.7	–12.2 ± 0.2	–80.9 ± 2.7	–230 ± 8
**Ic/Ie**	GGCAXAGGC/GCCUUUGCC	**55.1 ± 0.2**	–12.6/–4.4	–14.9 ± 0.2	–89.4 ± 1.7	–250 ± 5
**Ia/Ie**	GGCAGAGGC/GCCUUUGCC	**54.3 ± 0.1**	–13.4/–5.2	–14.0 ± 0.3	–81.3 ± 2.8	–226 ± 9
**IIa**	GGACCGGUCC (Palindrome)	**74.5 ± 0.2**	–	–18.7 ± 0.2	–83.9 ± 1.9	–219 ± 6
**IIb**	GGAUCGAUCC (Palindrome)	**59.7 ± 0.3**	–14.8	–15.8 ± 0.1	–87.0 ± 0.3	–239 ± 1
**IIc**	GGACCGXUCC (Palindrome)	**42.0 ± 0.2**	–32.5/–17.7	–11.6 ± 0.1	–89.9 ± 1.7	–263 ± 5
**IId**	GGAUCGXUCC (Palindrome)	**55.5 ± 0.2**	–19.0/–4.2	–15.1 ± 0.1	–90.7 ± 1.2	–254 ± 4
**IIe**	GGAUCGGUCC (Palindrome)	**56.0 ± 0.2**	–19.5/–4.7	–15.6 ± 0.1	–94.0 ± 0.7	–263 ± 2
**IIIa**	GAAGGGCAACCUUCG (Hairpin)	**71.1 ± 0.1**	–	–7.6 ± 0.1	–57.7 ± 0.3	–168 ± 1
**IIIb**	GAAXGGCAACCUUCG (Hairpin)	**39.4 ± 0.1**	–31.7	–1.8 ± 0.1	–37.2 ± 1.3	–120 ± 4
**IIIc**	GAXGGGCAACCUUCG (Hairpin)	**65.8 ± 0.4**	–5.3	–6.1 ± 0.1	–51.6 ± 1.7	–153 ± 5
**IIId**	GAGGGGCAACCUUCG (Hairpin)	**64.2 ± 0.1**	–6.9	–4.9 ± 0.1	–43.7 ± 0.7	–130 ± 2

^a^Buffer: 10 mM Na_2_HPO_4_, 150 mM NaCl, pH 7.0. Δ*H* and Δ*S* values were obtained by van’t Hoff analysis or based on RNA concentration dependent measurements according to references 18 and 19.

^b^The UV-spectroscopically determined *T*_m_ values are noted for an RNA concentration of 12 μM.

^c^Errors for Δ*H* and Δ*S* were determined from at least three independent measurements; in general, errors arising from noninfinite cooperativity of two-state transitions and from the assumption of a temperature-independent enthalpy, are typically 10−15%. Additional error is introduced when free energies are extrapolated far from melting transitions; errors for Δ*G* are typically 3−5%.

The same trend is observed for the palindromic set of RNAs. The native type II RNAs melt at 74.5°C (C4–G7, **IIa**) and at 59.7°C (U4–A7, **IIb**). Replacement of the purine-7 by xanthosine causes destabilization that is larger for C opposite of X (–32.5°C [–16.25°C per modification] relative to C4–G7, and –17.7°C [–8.85°C per modification] relative to U4–A7, **IIc**) and smaller for U opposite of X (–19.0°C [–9.5°C per modification] relative to C4–G7, and –4.2°C [–2.1°C per modification] relative to U4–A7, **IId**).

Next, we analyzed the impact of xanthosine in a monomolecular RNA system (type III, GNRA hairpin with 5 bp stem) melting at 71.1°C (**IIIa**). Substitution of G4 by X results in a X4–C11 pair accompanied by a drastic decrease of 31.7°C in *T*_m_ value (**IIIb**), while substitution of A3 by X results in a X3–U12 pair that gave a minor decrease of 5.3°C only (**IIIc**). This reflects the same trend as observed for bimolecular systems, showing that out of the four natural nucleosides, uridine is tolerated best for stable pairing to xanthosine.

Furthermore, we were wondering if U–X containing RNAs resemble their U–G counterparts with respect to pairing strength given that the putative bidendate ^w1^U–X pair (Figure 2C, top) adopts the same geometry as a U–G pair (Figure 2F) (41). Indeed, we found that the corresponding *T*_m_ values and thermodynamic parameters were nearly identical (Δ*T* of 0.8°C for **Ic/Ie** and **Ia/Ie**; Δ*T* of 0.5°C for **IId** and **IIe**; Table 1). This is consistent with the same base pair conformation in solution.

### Strong pH-dependence of X–C base pair stability

**Figure 4.**
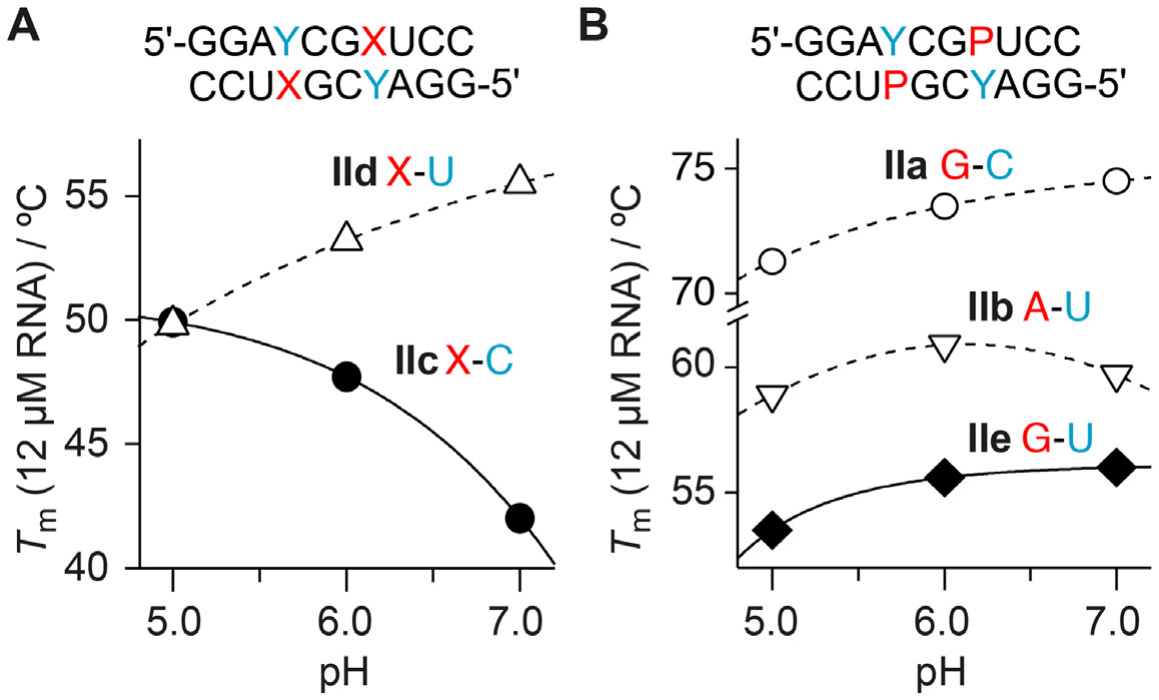
pH dependence of duplex base pairing. (**A**) The *T*_m_ value of the palindrome **IIc** (with X–C pairs) increases significantly with decreasing pH value, consistent with the involvment of a protonated C (p*K*_a_ C^+^ 4.1) in the base pair interaction with X. In contrast, the *T*_m_ value of the palindrome **IId** (with X–U pairs) decreases with decreasing pH value. This may indicate that the N3-deprotonated form of X at physiological pH is beneficial for base pairing (p*K*_a_ of X ∼5.2). (**B**) The palindromic duplexes containing base pairs G–C (**IIa**), A–U (**IIb**) and G–U (**IIe**) are significantly less pH dependent compared to X–C and X–U counterparts.

Next, we examined the influence of the pH value of the solution on pairing properties of X-modified RNA. For xanthosine, a p*K*_a_^N3-H^ value of 5.2 was reported (38), and hence, xanthine bases in RNA should be deprotonated in buffer solutions of physiologically relevant pH. Furthermore, the proposed pairing modes ^w1^C^+^–X (Figure 2D) and C^+^–^taut^X^–^ (Figure 2E, bottom) involve protonated cytidine holding a p*K*_a_^N3H+^ value of about 4.1 (42,43). Indeed, we found a pronounced pH dependence for the C–X containing duplex **IIc** with the melting temperature *T*_m_ increasing by more than 9°C when the pH value was changed from pH 7 to pH 5 (Figure 4A). In contrast, we found the reverse pH dependence for the U–X containing duplex **IId**, with the melting temperature *T*_m_ decreasing by more than 4°C when the pH value was changed from pH 7 to pH 5 (Figure 4A). Notably, the corresponding reference duplexes **IIa** (C–G), **IIb** (U–A) and **IIe** (U–G) displayed little pH dependence in the same pH range (Figure 4B). These findings support the notion that N3 protonated cytidine (C^+^) is involved in base pair formation with X (Figure 2D, E). Moreover, these findings suggest that deprotonation of xanthosine N3–H in U–X base pairs is accompanied by thermodynamic stabilization (Figure 2C).

Taken together, our data provide the first comprehensive insights into thermodynamic stabilities of xanthosine containing RNA. Thus far, only 2′-deoxyxanthosine was investigated in the context of DNA (44,45); at physiological pH values and typical sodium ion concentrations, xanthine bases caused destabilization when paired to A or C (–9°), and were less destabilizing when paired to G or T (–6° and –5°, respectively) (44). Hence, the signature of X base pair strength in DNA is distinct to RNA.

### NMR spectroscopy reveals X–U and X–C^+^ wobble pairs in RNA

**Figure 5.**
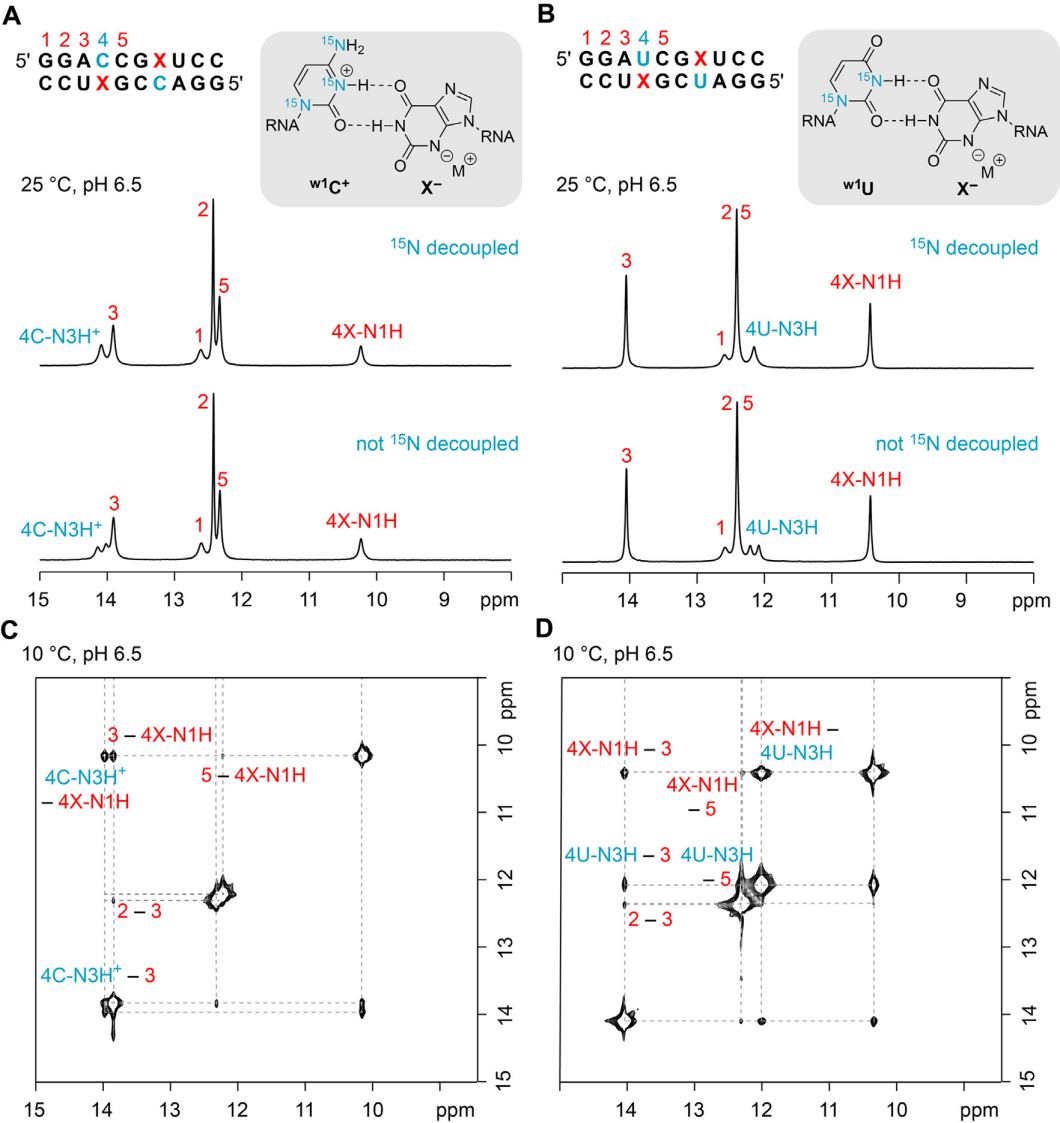
NMR spectroscopic analysis of RNA palindromes with C–X and U–X base pairs. Sequences and ^1^H NMR imino proton spectra of the RNAs with site-specifically ^15^N-modified C4 (**A**) and U4 (**B**) as illustrated in the chemical structures; signal assignment was based on ^15^N-decoupling and on ^1^H,^1^H-NOESY NMR spectra (**C, D**); conditions: c_RNA_ = 1.0 mM, 25 mM NaCl, 15 mM sodium phosphate buffer, H_2_O/D_2_O 9/1, pH 6.5.

Hydrogen-bonded protons in Watson–Crick base-pairs are detected straightforward by NMR spectroscopy. The corresponding NMR resonances of the ‘imino protons’ directly reflect double helical segments within folded RNA. The chemical shifts of these signals are characteristic for A–U (> 14 ppm) and C–G base pairs (∼12–13 ppm), and the linewidths reflect proton exchange with the solvent. Obviously, imino protons are very sensitive to modifications, in particular, if the modification concerns the nucleobase. Comparative imino proton ^1^H NMR spectra of the palindromic duplex 5′-GGAUCGAUCC **IIb** and the corresponding C–X, U–X, and U–G modified counterparts, **IIc IId** and **IIe** are depicted in Figure 5A,B and Supporting Figure S17. They show that C–X and U–X form defined pairing interactions, each characterized by two new resonances while the remaining standard Watson–Crick base pairs retain their imino proton chemical shifts in comparison to the U–A and U–G reference RNAs. This indicates that the C–X and U–X pairs integrate well into an A-form RNA duplex, hardly effecting the neighboring base pairs. Using site-specifically ^15^N1, ^15^N3, ^15^N-C4 cytidine-4 and ^15^N1, ^15^N3 uridine-4 labeled RNA and 2D ^1^H,^1^H NOESY NMR spectroscopy (Figure 5C,D), all resonances observed in the imino proton region were unequivocally assigned and supported the wobble base pair conformations ^w1^U–X and ^w1^C^+^–X depicted in Figure 5. We note that the pronounced low field chemical shift of the ^15^N3-H resonance for protonated cytidine-4 is in accordance with other NMR spectroscopic investigations on protonated cytidines as e.g. observed for C^+^–G Hoogsteen base pairs (46), or for RNA U-turns (47,48). Additionally, we point out that for both wobble pairs (U–X and C^+^–X), the imino proton of both, purine (N1-H) and pyrimidine (N3-H), are observable. This is comparable with the imino proton signature observed for the wobble U–G reference RNA (Supporting Figure S17).

We further mention that by NMR spectroscopic means we cannot distinguish between ^w1^U–X and ^w2^U–X conformations (Figure 2C) for GGAUCCGXUCC **IId**. However, we consider ^w1^U–X more likely because of the shape equivalence with a typical wobble U–G pair and the similar thermodynamic parameters obtained in our melting profile analysis. Furthermore, we mention that for paring of X with C only one wobble geometry, namely ^w1^C–X is feasible while no complementary H-donor/acceptor pattern for a ^w2^U–X like interaction is possible. Additionally, for the palindrome GGACCCGXUCC **IIc** further evidence for the wobble pair ^w1^C^+^-X (Figures 2D, 4A) formation stems from the ^1^H,^15^N-HSQC spectrum of ^15^N3,^15^N–C4 cytidine-4 modified RNA **IIc**, whichs shows only a small chemical shift difference of <0.1 ppm for NH(1) and NH(2) of the C4–NH_2_ group (Supporting Figure S18). This is consistent with an C4–NH_2_ group that is not involved in H-bonding with one of its H atoms (Figure 2D, 5A). If the C4–NH_2_ group of the cytosine base were involved in hydrogen bonding as would be expected for the putative Watson–Crick pairing mode (Figure 2E), the chemical shift difference between the two protons NH(1) and NH(2) of the amino group would be significantly larger (for a comparison see, e.g. references (49,50), and Supporting Figure S19).

Finally, we investigated if the pH dependence of base pairing strength is also reflected in the imino proton spectra of the X–C containing palindrome GGACCCGXUCC **IIc**. Strikingly, at pH 8.0 the imino proton shifts and line widths are nearly identical to those observed at pH 6.5, including the ^15^N3–H resonance for the protonated cytidine-4 (Supporting Figure S20). However, at acidic conditions (pH 4.5), a significant upfield shift for the ^15^N3-H resonance is observed, and interestingly, the C4–NH_2_ resonances of the N3 protonated cytidine-4 are emerging at 9.3 ppm (Supporting Figure S18 and S20).

To summarize, our NMR study on short RNA palindromes indicates that in solution, xanthosine can form well defined base pairs. Both, X opposite of U and X opposite of C adopt a wobble geometry (Figure 4A, B) which is comparable to a standard G–U base pair (Figure 2F). To achieve this, C is protonated at the N3 atom (Figure 5A).

### Crystal structures of xanthosine containing RNA

**Figure 6.**
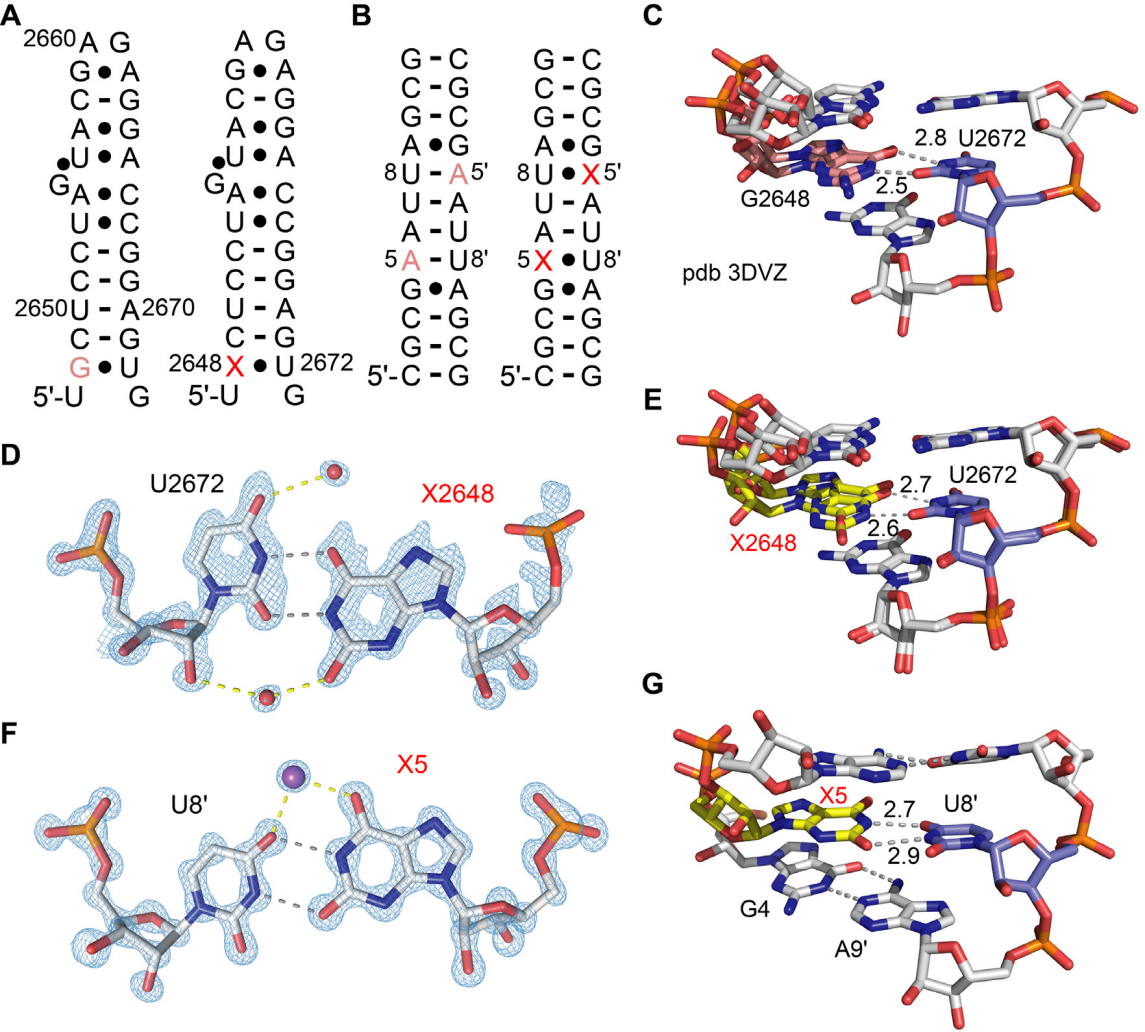
Crystal structures of X–U modified RNAs at 0.9 Å and 1.0 Å resolution. (**A**) Secondary structure of the *E. coli* sarcin–ricin stem–loop (SRL) RNA and (**B**) a 12 bp palindrome used for crystallization. The X nucleotide is labeled in red. (**C**) Side view on the G2648/U2672 base pair in the unmodified duplex (PDB ID 3DVZ). (**D**) 2*F*_obs_ – *F*_calc_ electron density map contoured at 1.5 σ level showing the X2648/U2672 base pair (PDB ID 7QSH). (**E**) Side view on the X2648/U2672 base pair in the SRL RNA (PDB ID 7QSH). (**F**) 2*F*_obs_ – *F*_calc_ electron density map contoured at 1.5 σ level showing the X5/U8′ base pair (PDB ID 7QUA). Numbers are distances in Angström (Å). (**G**) Side view on the X5/U8′ base pair in the 12 bp palindrome (PDB ID 7QUA). We note that the alternative conformations of G2648, C2649 (and U2650) are also observed in the unmodified SRL RNA scaffold (PDB ID 3DVZ). Numbers are distances in Angström (Å).

To further shed light on xanthosine containing base pairs in RNA we dedicated considerable effort towards crystallographic analysis. Unfortunately, the RNAs **IIc** and **IId** that we used above for NMR spectroscopic investigations did not crystallize. We therefore utilized the 27 nt fragment of the *E. coli* 23S rRNA sarcin−ricin loop (SRL) (Figure 6A) (20,21), the 12 nt RNA 5′-CGCGAAUUACGC (Figure 6B) (22,51), and the 14 nt RNA 5′-GGUAUUGCGGUACC (23), all of them are known crystallization scaffolds. For replacements with xanthosine, we tested several positions in a large number of RNAs, and we obtained crystals for three of them that diffracted to subatomic resolution (Supporting Table S3). Two contain an X–U pair and one an X–C pair. X-ray structure determination showed that the X nucleobases are well-defined in the electron density maps. For the SRL RNA structure (solved at 0.9 Å resolution), X2648 pairs with U2672 in wobble geometry (^w1^U–X) forming X-N1H•••O2-U and X-O6•••HN3-U hydrogen bonds similar to a typical wobble G–U pair (Figure 6C–E). Characteristically, a water molecule bridges the O2 of X2648 and the 2′-OH of U2672 (Figure 6D). Superimpositions of the X-modified RNA structure with the unmodified RNA showed a root-mean-square deviation (rmsd) of 0.10 Å (within the errors on coordinates of 0.10 Å). Of note, the unmodified RNA scaffold contains a G–U wobble pair at the very position (2648–2672) (Figure 6A,C). Of further note, the alternative conformations of the nucleosides in position 2649 and 2648 are observed for both unmodified and modified SRL RNA (C2649 and G2648, PDB ID 3DVZ; C2649 and X2648, PDB ID 7QSH) (Figure 6C, E). Finally we mention that all our attempts to crystallize the SRL scaffold with a replacement of individual A–U or G–C pairs (at diverse positions) by X–U or X–C failed.

Therefore, we switched to a different crystallization scaffold, namely 5′-CGCGAAUUACGC (22,51), and fortunately, we obtained another structure of a X–U modified RNA at high resolution. Replacement of A5 by X5 gave crystals that diffracted at 1.0 Å resolution. Surprisingly, the X5–U8′ base pair adopted a wobble conformation (^w2^U–X, Figure 2C) distinct to a typical G–U pair, involving X-N1H•••O4-U and X-O2•••HN3-U hydrogen bonds (Figure 6F,G). A possible rationale for this alternative wobble geometry is that the neighboring base pair is not a standard Watson–Crick pair but a purine-purine pair (G4-A9) that widens the helix. This specific environment likely renders the ^w2^U–X geometry (Figure 2C, 6F) energetically favorable. Interestingly, the X5-U8′ base pair coordinates a metal ion which is assigned to sodium based on the coordination geometry and the distances of 2.4 to 2.5 Å to the six coordination sites (52,53). These are O6 of X5, O4 of U8′, and the O4 atom of U7′ (Supporting Figure S21) with the remaining sites of the ion occupied by water molecules. Of note, both the riboses of xanthosine and the paired uridine are in C3′-endo conformation. Analysis of the 5′-CGCGXAUUACGC by ^1^H-NMR spectroscopy indicated that the system is rather dynamic in solution likely existing in equilibrium between hairpin and duplex conformations (Supporting Figure S22).

**Figure 7.**
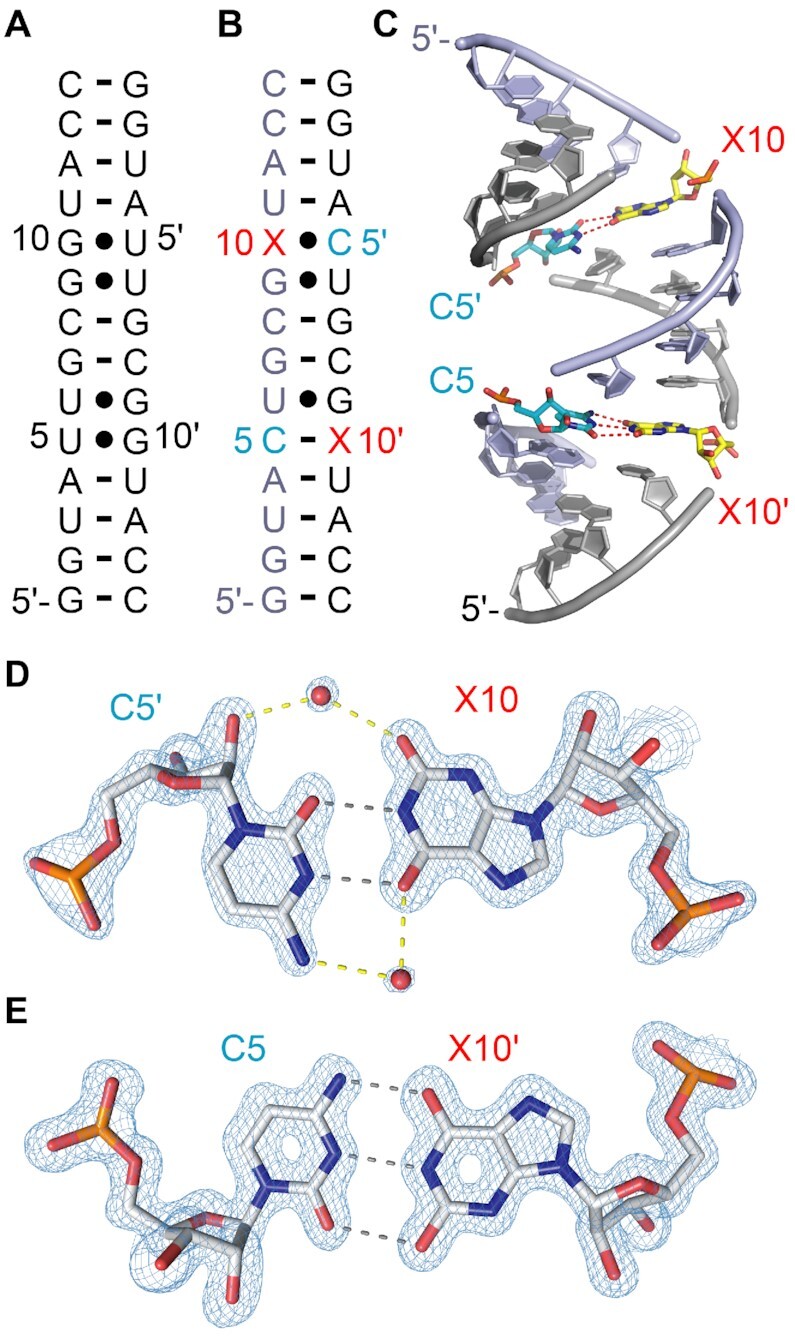
Crystal structure of a X–C modified 14 nt RNA at 1.2 Å resolution. (**A**) Secondary structure of the 14 bp RNA palindrome (22,51). (**B**) Secondary structure of the X and C-modified RNA counterpart used for crystallization. The X nucleotide is labeled in red. (**C**) Side view on the duplex highlighting the two X–C base pairs. (**D**) 2*F*_obs_ – *F*_calc_ electron density map contoured at 1.5 σ level showing the C5′/X10 base pair (PDB ID 7QTN). (**E**) 2*F*_obs_ – *F*_calc_ electron density map contoured at 1.5 σ level showing the C5/X10′ base pair (PDB ID 7QTN).

To maximize our chances to achieve a crystal structure also for the X–C base pair we tested a further RNA scaffold, namely 5′-GGUAUUGCGGUACC (23). Replacement of G10 and U5 by X10 and C5 (Figure 7A,B) indeed gave crystals that diffracted at 1.2 Å resolution. The structure of the self-complementary RNA shows a fully base-paired duplex with overall C2 symmetry (Figure 7C), but strikingly, the two individual C–X pairs adopt distinct conformations (Figure 7D, E). While C5′-X10 forms a wobble base pair (Figure 7D), C5-X10′ forms a tridendate base pair that is equivalent in shape to a canonical G–C Watson Crick pair (Figure 7E). The C5′–X10 wobble base pair is shape complementary to a typical G–U wooble pair (^w1^C–X in Figure 2D, F). Further, two ordered water molecules are observed, one bridging the the O2 of X10 and the 2′-OH of C5′ in the minor groove (Figure 7D) while the other one links the O6 of X10 and the C4-NH_2_ of C5′. Of note, for both X–C pairs, the riboses are all in C3′-endo conformation.

With respect to the Watson–Crick shaped C5–X10′ pair, a tautomeric form of xanthosine must be involved, however, at the current state we are not able to distinguish between two interaction possibilities involving neutral nucleobases (Figure 2E, top) versus charged nucleobases (Figure 2E, bottom). ^1^H-NMR spectroscopic investigations on the 14 nt unmodified RNA scaffold indicates competition between bimolecular (duplex) and the monomolecular (hairpin) conformations (Supporting Figure S23A). The equilibrium seems to be shifted towards the duplex if G10 and U5 are replaced by X10 and C5 (Supporting Figure S23B). Furthermore, the ^1^H,^1^H-NOESY spectrum shows the typical correlations for G–U and X–U wobble pairs (Supporting Figure S23C), however, the structural dynamics of the 14 nt RNA prohibited a more detailed NMR spectroscopic analysis of the nature of the two X–C base pairs in solution.

Conclusively, our X-ray studies on short X–U and X–C containing RNAs revealed that xanthosine can form a significantly larger diversity of base pair conformations that were implied by the original NMR spectroscopic investigations. Beside ^w1^U–X and ^w1^C–X wobble pairing, the X-ray structures show ^w2^U–X wobble and Watson–Crick-type pairing modes (C–X^taut^ or C^+^–X^taut–^) depending on the sequence context. We speculate that crystal packing influenced the conformations captured in the crystal. In particular, for the 12 and 14 nt RNAs, solution NMR spectroscopy indicated structural dynamics that we could not resolve further with standard NMR experiments applied. The alternative pairing modes might exchange on a fast time scale (below milliseconds) in solution. To verify this, more detaled NMR studies are planned in the future.

Finally, to the best of our knowledge, we mention that only one crystallographic study on DNA with 2′-deoxyxanthosine is found in the literature that sheds light on the pairing mode with one of the standard nucleosides. This crystal structure concerns human DNA polymerase η (polη) with a single 2′-deoxyxanthosine containing DNA template. In the catalytic site of polη, the xanthine base forms three Watson–Crick-like hydrogen bonds with an incoming dCTP, indicating the O2-enol tautomer of xanthine involves in the base pairing (58).

### Reverse transcription of xanthosine

**Figure 8.**
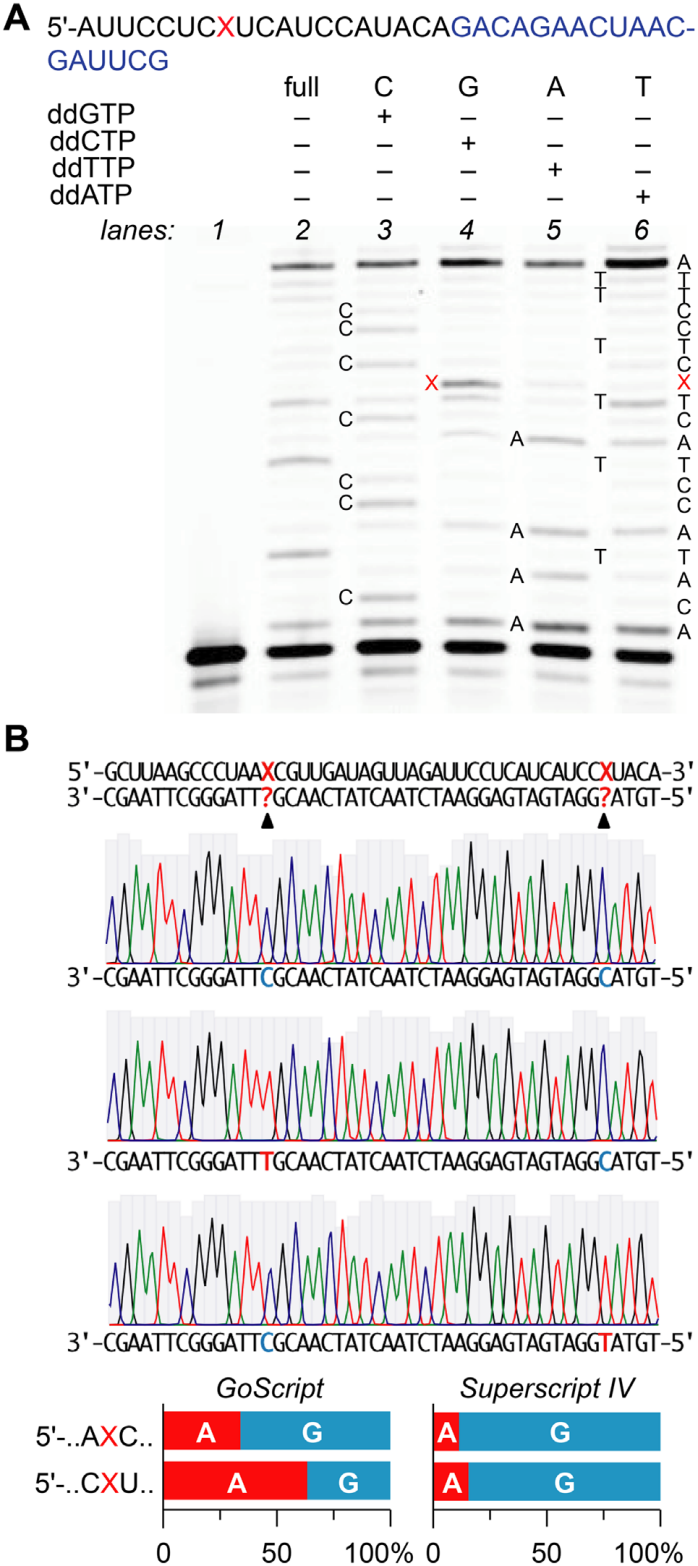
Differences in X recognition by different reverse transcriptases. (**A**) Typical gel of a primer extension assay using the RNA as indicated (primer sequence in blue) and Superscript IV reverse transcriptase; lanes from left to right are for the X-modified template (lane 2), and the corresponding C, G, A, T ladders (lanes 3 to 6); primer (lane 1). (**B**) Reverse transcription (RT)-PCR and sequencing analysis to estimate incorporation frequency of G or A opposite X. A 83 nt long RNA template containing two X positions in different sequence contexts (X1: AXC, X2: CXU) was used for reverse transcription with GoScript or Superscript IV reverse transcriptases and subsequent sequencing. *Top*: Example sequence traces of three independent clones. *Bottom*: Quantification of G and A recognition ( = X is read as G or A) at the indicated positions (*n* = 15 for GoScript, *n* = 30 for Superscript IV).

To shed light on the biological consequences of the diverse pairing modes of xanthosine-containing RNA, we tested the impact of this modification on reverse transcription in primer extension assays using Superscript IV reverse transcriptase (Figure 8A). In the sequence context 5′-…CXU…, the dominant product was the full-length RNA with X being decoded as G, as judged by the ddCTP sequencing lane. We then employed a different approach to examine incorporation frequency opposite X using reverse transcription followed by PCR amplification, amplicon subcloning and Sanger sequencing of individual clones. To this end, we synthesized a 83 nt RNA containing two xanthosines in the context of 5′-…CXU… and 5′-…AXC… (Figure 8B). Because we reasoned that different reverse transcriptases might behave differently towards X, we used Superscript IV as well as GoScript. Indeed, sequencing of individual clones revealed pronounced X-recognition differences between the two enzymes (Figure 8B). Whereas GoScript preferentially read X as A in the sequence context CXU, the opposite was true for the sequence context AXC. By contrast and consistent with the primer extension analysis, Superscript IV showed clear preference for reading X as G in both sequence contexts. Of note, a recent study on the behaviour of different reverse transcriptase enzymes with RNA templates containing *N*^1^-methyladenosine (m^1^A) found that GoScript showed lowest mismatch and highest arrest rates, while Superscript IV had the highest mismatch and lowest arrest rates (54). We find that the weak mismatch tolerance of GoScript is also apparent with X, since the thermodynamically most favorable base pair X–U (and by inference X–T) is preferred, at least in the sequence context CXU. This effect is less pronounced in the sequence context AXC, most likely due to the strong C–G pair that can be formed by the polymerase before it encounters X and that may help to accommodate/stabilize a thermodynamically less favorable X–C pairing. Superscript IV, on the other hand, exhibits no discernible context dependence for reading X, but generally favors the X–C base pair, which underscores its greater mismatch tolerance (54).

### Concluding remarks

Nucleobases in RNA are amenable to deamination by diverse mechanisms. Most prominent is RNA adenosine-to-inosine editing which is a conserved process for recoding of genetic information in nature. Nucleobase deamination is performed by deaminase enzymes but it can also occur spontaneously by hydrolysis or be induced by environmental factors e.g. nitrosive chemistry as has been utilized for recent RNA-sequencing approaches. When deamination affects guanosine, xanthosine (X) is obtained. The cellular consequences of this transformation in RNA are scarcely known, and the chemical and biophysical consequences on RNA have been unclear. Our comprehensive study now sheds light on the properties of X-modified RNA. As thorough foundation, we developed the synthesis of a novel phosphoramidite building block allowing efficient access to X-modified RNAs by solid-phase synthesis. Thermodynamic analyses of such RNAs revealed that compared to the fully Watson–Crick paired counterparts, pairing strength is reduced to an extent that is similar to that observed for a G•U replacement. Applying NMR spectroscopy and X-ray crystallography, we demonstrate that X can form two distinct wobble geometries with uridine depending on the sequence context. In contrast, X pairing with cytidine occurs either through wobble geometry involving protonated C or in Watson–Crick shape-like mode. Strikingly, we identified the two distinct X–C pairing modes in the X-ray structure of a palindromic RNA duplex, breaking C2 symmetry. This indicates that the two pairing modes are of comparable stability separated by low energetic barriers. We furthermore demonstrate that the flexible pairing of X directly affects the reading of X-modified RNA by reverse transcription enzymes. Using primer extension assays and PCR-based sequencing analysis, we show that X is preferentally read as G or A and that the ratio depends on the type of reverse transcriptase. Taken together, our results elucidate important properties of X-modified RNA paving the way for future studies on the biological significance of xanthosine-containing RNA.

## DATA AVAILABILITY

Atomic coordinates and structure factors for the reported crystal structures have been deposited with the Protein Data bank under accession numbers 7QSH, 7QUA and 7QTN.

## Supplementary Material

gkac477_Supplemental_File
