# Supplementary material for: Towards a comprehensive understanding of RNA deamination: synthesis and properties of xanthosine-modified RNA
Source: Nucleic Acids Res. 2022 Jun 10;50(11):6038–51. doi: 10.1093/nar/gkac477 (PMC9226506; doi:10.1093/nar/gkac477)
Supplement: gkac477_Supplemental_File [file gkac477_supplemental_file.pdf]

Towards a comprehensive understanding of RNA deamination – Synthesis and properties of xanthosine-modified RNA

Stefan Mair,[1] Kevin Erharter,[1] Eva Renard,[2] Karl Brillet,[2] Melanie Brunner,[3] Alexandra Lusser,[3] Christoph Kreutz,[1] Eric Ennifar[2] Ronald Micura[1]

Content

Supporting Methods

1.1. 2′-*O*-(*tert*-Butyldimethylsilyl)-3′,5′-*O*-(di-*tert*-butylsilanediyl)guanosine **1** 2
1.2. *N*2-(4,4′-Dimethoxytrityl)-2'-O-(tert-butyldimethylsilyl)-3′,5′-*O*-(di-*tert*-butylsilanediyl)guanosine **2** 5
1.3. *O*6-(4-Nitrophenyl)ethyl-*N*2-(4,4′-dimethoxytrityl)-2′-*O*-(*tert*-butyldimethylsilyl)-3′,5′-*O*-(di-*tert*-butylsilanediyl)guanosine **3** 8
1.4. *O*6-(4-Nitrophenyl)ethyl-2′-*O*-(*tert*-butyldimethylsilyl)-3′,5′-*O*-(di-*tert*-butylsilanediyl)guanosine **4** 11
1.5. *O*6-(4-Nitrophenyl)ethyl-2′-*O*-(*tert*-butyldimethylsilyl)-3′,5′-*O*-(di-*tert*-butylsilanediyl)xanthosine **5** 14
1.6. *O2,O6*-Bis(4-nitrophenyl)ethyl-2′-*O*-(*tert*-butyldimethylsilyl)-3′,5′-*O*-(di-*tert*-butylsilanediyl)xanthosine **6** 17
1.7. *O2,O6*-Bis(4-nitrophenyl)ethyl-2′-*O*-(*tert*-butyldimethylsilyl)xanthosine **7** 20
1.8. *O2,O6*-Bis(4-nitrophenyl)ethyl-5′-*O*-(4,4′-dimethoxytrityl)-2′-*O*-(*tert*-butyldimethylsilyl)xanthosine **8** 23
1.9. *O2,O6*-Bis(4-nitrophenyl)ethyl-5′-*O*-(4,4′-dimethoxytrityl)-2′-*O*-(*tert* butyldimethylsilyl)xanthosine-3′-*O*-(2-cyanoethyl) *N*,*N*-diisopropylphosphoramidite **9** 26
1.10. 1-(2-Iodoethyl)-4-nitrobenzene **10** 30

Supporting Tables

Supporting Table 1. Overview of synthesized RNAs and mass spectrometric analysis. 33
Supporting Table 2. Complete set of thermodynamic data of xanthosine modified RNA. 34
Supporting Table 3. X-ray data collection and crystallographic refinement statistics. 35

Supporting Figures

Supporting Figures S1 to **S16**. UV-melting profile analysis of X-modified RNAs. 36 – 51
Supporting Figures S17. 1H NMR imino proton spectra of 10 nt RNA palindromes **IIb**, **IId**, **IIe**, and **IIc** 52
Supporting Figures S18. NMR spectroscopic analysis of the 10 nt RNA palindrome **IIc** 53
Supporting Figures S19. NMR spectroscopic analysis of the 10 nt RNA palindrome **IIa** 54
Supporting Figures S20. NMR spectroscopic analysis of the 10 nt RNA palindrome **IIc** 55
Supporting Figures S21. Crystal structure of a X-U modified 12 nt RNA palindrome 56
Supporting Figures S22. NMR spectroscopic analysis of the 12 nt RNA palindrome 57
Supporting Figures S23. NMR spectroscopic analysis of the 14 nt RNA palindrome 58

1.1. 2′-*O*-(*tert*-Butyldimethylsilyl)-3′,5′-*O*-(di-*tert*-butylsilanediyl)guanosine 1

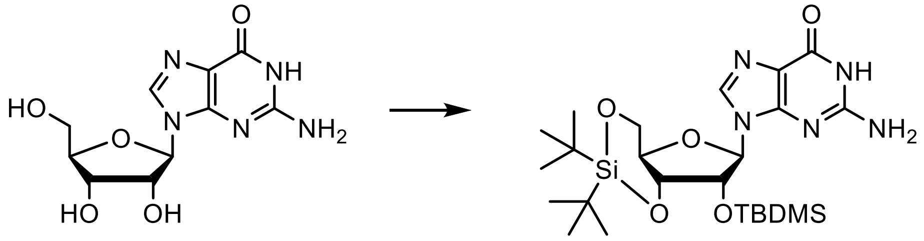

1

Guanosine (2.40 g, 8.47 mmol) was coevaporated three times each with pyridine, toluene and dichloromethane and dried overnight on the high vacuum. Subsequently the compound was suspended in *N,N*-dimethylformamide (30 mL) and di-tert-butylsilanediyl bis(trifluoro-methanesulfonate) (4.11 g, 9.32 mmol, 1.1 eq) was added over the course of 15 min at 0 °C. The suspension was then stirred for 45 min upon which a clear solution is formed. Imidazole (2.88 g, 42.4 mmol, 5 eq) was added and the solution was first stirred at 0 °C for 5 min and then at room temperature for 25 min. The reaction mixture was treated with *tert*-butyldimethylsilyl chloride (1.53 g, 10.2 mmol, 1.2 eq) and then heated to 60 °C for 2 h. The product precipitated upon cooling to room temperature, filtrated and washed with cold methanol. Yield: 3.23 g of compound **1** as a white solid (71%). TLC: 0.11 (4/96 methanol/dichloromethane). HR-ESI-MS (m/z): $[M+H]^+$ (calc): 538.2875; $[M+H]^+$ (meas): 538.2860. ^{1}H NMR (400 MHz, DMSO): δ 0.07 (s, 3H, H_3CSi), 0.09 (s, 3H, H_3CSi), 0.86 (s, 9H, $(H_3C)_3CSi$(TBDMS)), 1.00 (s, 9H, $(H_3C)_3CSi$), 1.06 (s, 9H, $(H_3C)_3CSi$), 3.93-3.97 (m, 2H, H(5'a) and H(4')), 4.27-4.35 (m, 2H, H(3') and H(5'b)), 4.57 (d, 1H, H(2')), 5.72 (s, 1H, HC(1')), 6.35 (brs, 2H, $H_2NC(2)$), 7.91 (s, 1H, HC(8)), 10.65 (s, 1H, HNC(1)), ppm. ^{13}C NMR (100 MHz, $CDCl_3$): δ -5.15 (s, 1C, CH_3Si(TBDMS)), -4.61 (s, 1C, CH_3Si(TBDMS)), 18.01 (s, 1C, $C(CH_3)_3$), 19.96 (s, 1C, $C(CH_3)_3$), 22.21 (s, 1C, $C(CH_3)_3$), 25.68 (s, 3C, $(CH_3)_3CSi$), 26.82 (s, 3C, $(CH_3)_3CSi$), 27.29 (s, 3C, $(CH_3)_3CSi$, 66.94 (s, 1C, C(5')), 73.88 (s, 1C, C(4')), 74.70 (s, 1C, C(2')), 75.65 (s, 1C, C(3')), 90.05 (s, 1C, C(1')), 116.55 (s, 1C, C(5)), 135.56 (s, 1C, C(8)), 150.75 (s, 1C, C(4)), 153.76 (s, 1C, C(2)), 156.66 (s, 1C, C(6)), ppm.

^{1}H-NMR (400 Mhz, DMSO-d6, 25 °C) of compound **1**

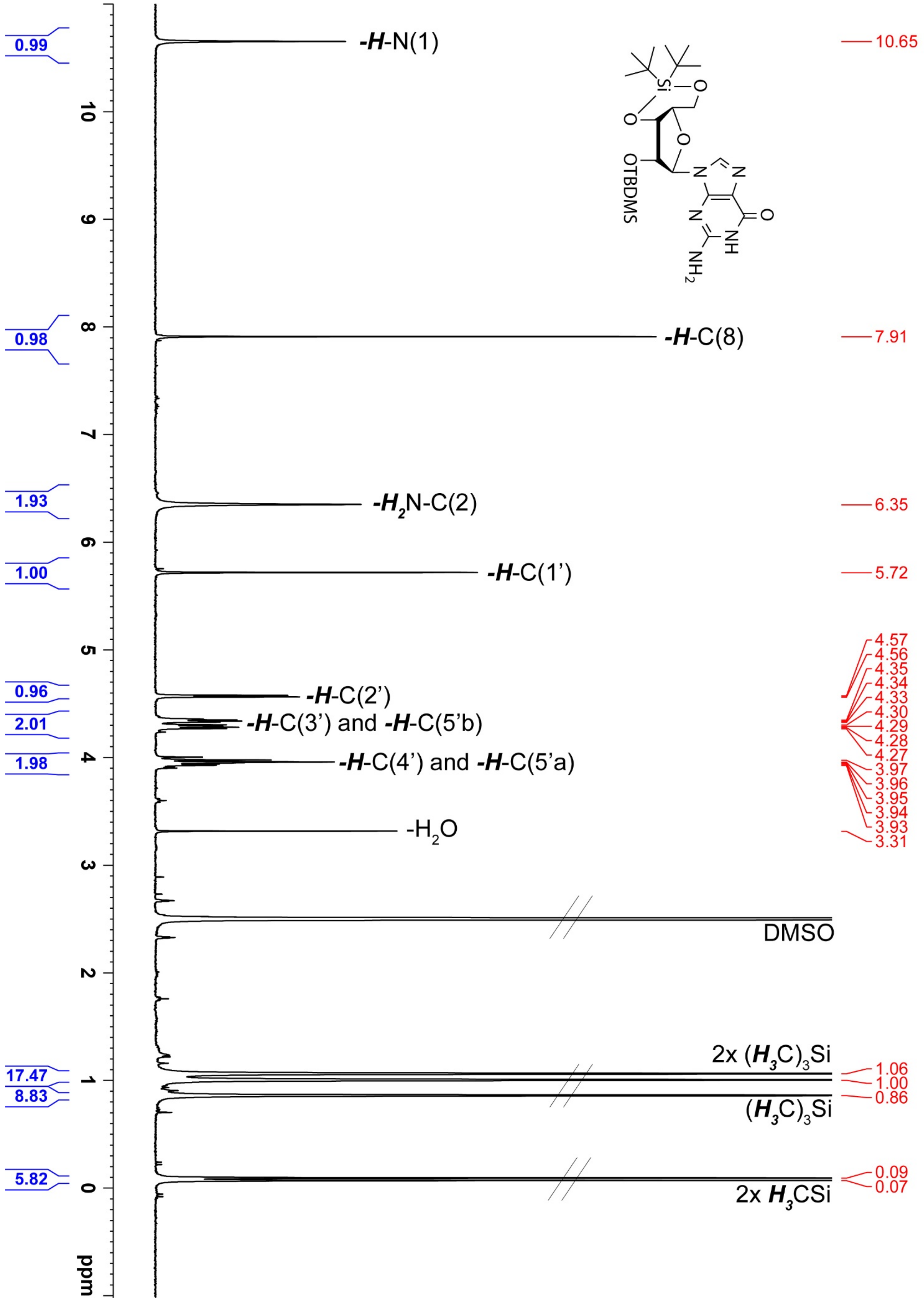

^{13}C-NMR (100 Mhz, DMSO-d$_6$, 25 °C) of compound **1**

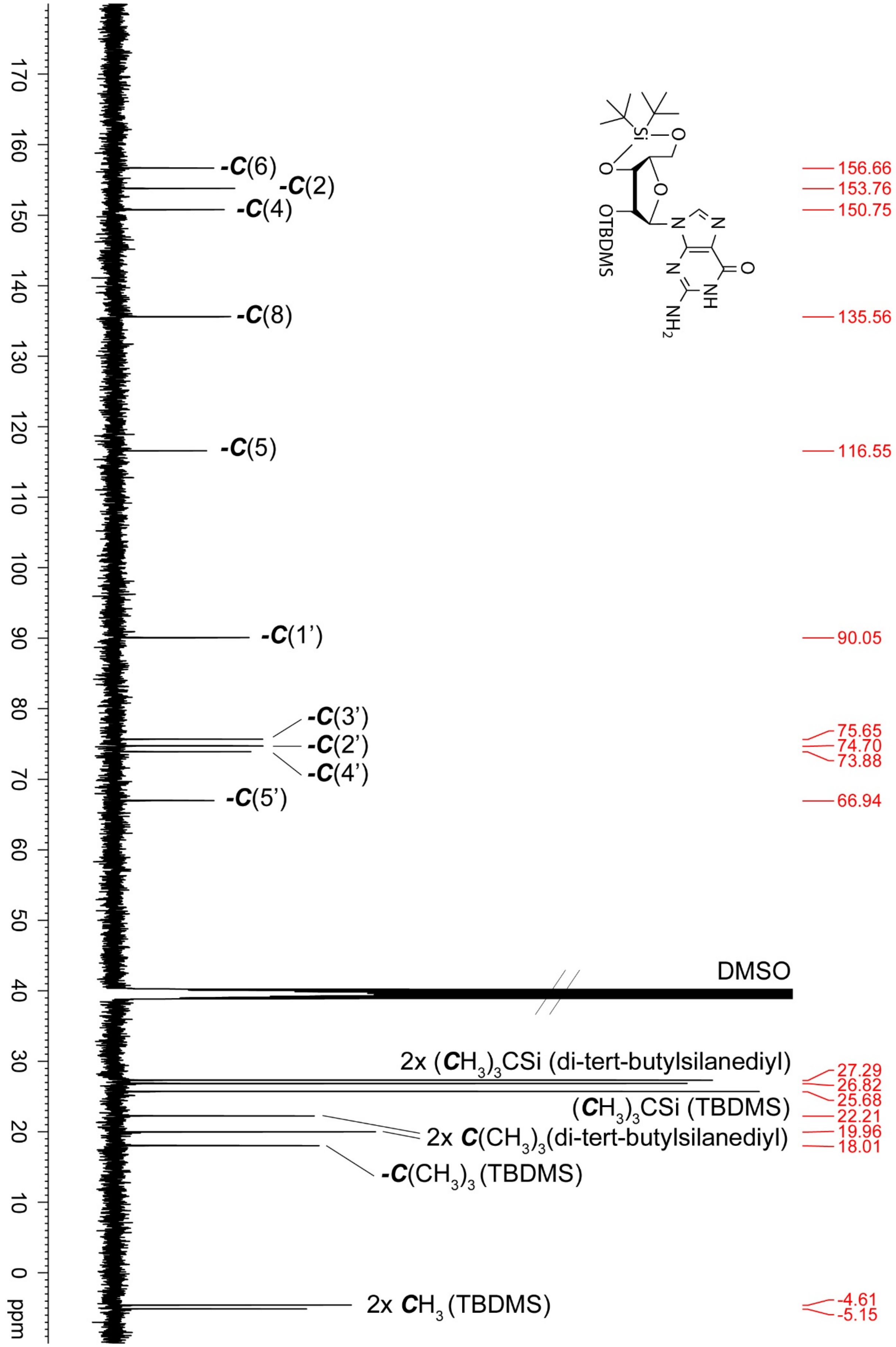

1.2. N^2-(4,4′-Dimethoxytrityl)-2'-*O*-(*tert*-butyldimethylsilyl)-3',5'-*O*-(di-*tert*-butylsilanediyl)guanosine 2

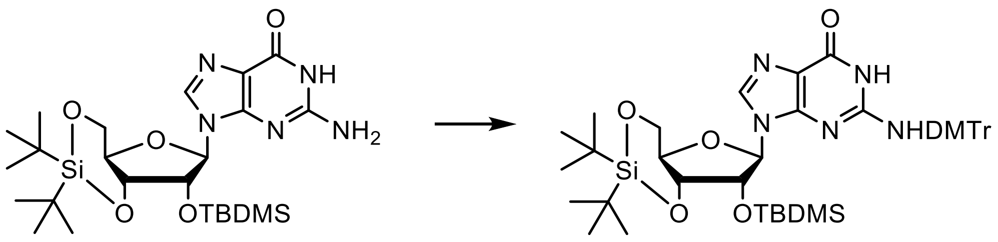

1 **2**

Compound **1** (3.23 g, 6.01 mmol) and 4,4′-dimethoxytrityl chloride (2.85 g, 8.41 mmol, 1.4 eq) were dissolved in anhydrous pyridine (50 mL) and the solution was stirred for 20 h at room temperature. The solvent was evaporated and the brown oil was coevaporated in ethyl acetate until a light yellow solid was obtained. The residue was taken up in ethyl acetate (50 mL) and extracted with 5% aqueous citric acid, water and brine. The organic phase was dried over $MgSO_4$ and the solvent was removed on the rotavapor. The crude product was further purified via column chromatography (SiO_2, 1-4% methanol in dichloromethane with 0.5% triethylamine). Yield: 4.80 g of compound **2** as a white foam (95%). TLC: 0.33 (4/96 methanol/dichloromethane). HR-ESI-MS (m/z): $[M+H]^+$ (calc): 840.4182; $[M+H]^+$ (meas): 840.4150. ^1H NMR (400 MHz, $CDCl_3$): δ 0.13 (s, 3H, H_3CSi), 0.13 (s, 3H, H_3CSi), 0.92 (s, 9H, $(H_3C)_3CSi$(TBDMS)), 1.02 (s, 9H, $(H_3C)_3CSi$), 1.03 (s, 9H, $(H_3C)_3CSi$), 3.80 (s, 6H, H_3CO(DMTr)), 3.99 (t, 1H, HC(5'a), $^2J_{HH}$ = 10 Hz), 4.13-4.17 (m, 1H, HC(4')), 4.21-4.24 (m, 1H H(3')), 4.38 (d, 1H, HC(2'), $^3J_{HH}$ = 5 Hz), 4.45-4.48 (m, 1H, HC(5'b)), 5.68 (s, 1H, H(1')), 6.22 (brs, 1H, HN(1)), 6.83-6.87 (m, 4H, HC(ar)), 7.19-7.34 (m, 9H, HC(ar)), 7.43 (s, 1H, HC(8)), ppm. ^{13}C NMR (100 MHz, $CDCl_3$): δ -4.83 (s, 1C, CH_3Si(TBDMS)), -4.08 (s, 1C, CH_3Si(TBDMS)), 18.51 (s, 1C, $C(CH_3)_3$), 20.45 (s, 1C, $C(CH_3)_3$), 22.87 (s, 1C, $C(CH_3)_3$), 26.05 (s, 3C, $(\mathit{C}H_3)_3CSi$), 27.13 (s, 3C, $(\mathit{C}H_3)_3CSi$), 27.59 (s, 3C, $(\mathit{C}H_3)_3CSi$), 55.41 (s, 2C, CH_3O(DMTr)), 67.99 (s, 1C, C(5')), 70.30 (s, 1C, C(central, DMTr)), 74.52 (s, 1C, C(4')), 75.83 (s, 1C, C(2')), 76.27 (s, 1C, C(3')), 91.70 (s, 1C, C(1')), 114.21 (s, 4C, CH(ar)), 118.50 (s, 1C, C(5)), 127.98-129.79 (m, 5C, CH(ar)), 135.16-135.24 (m, 2C, C(8) and C(ar)), 144.05 (s, 1C, C(ar)), 149.91 (s, 1C, C(4)), 151.98 (s, 1C, C(2)), 156.08 (s, 1C, C(6)), 159.21 (s, 1C, $COCH_3$(ar)), ppm.

^{1}H-NMR (400 Mhz, $CDCl_3$, 25 °C) of compound **2**

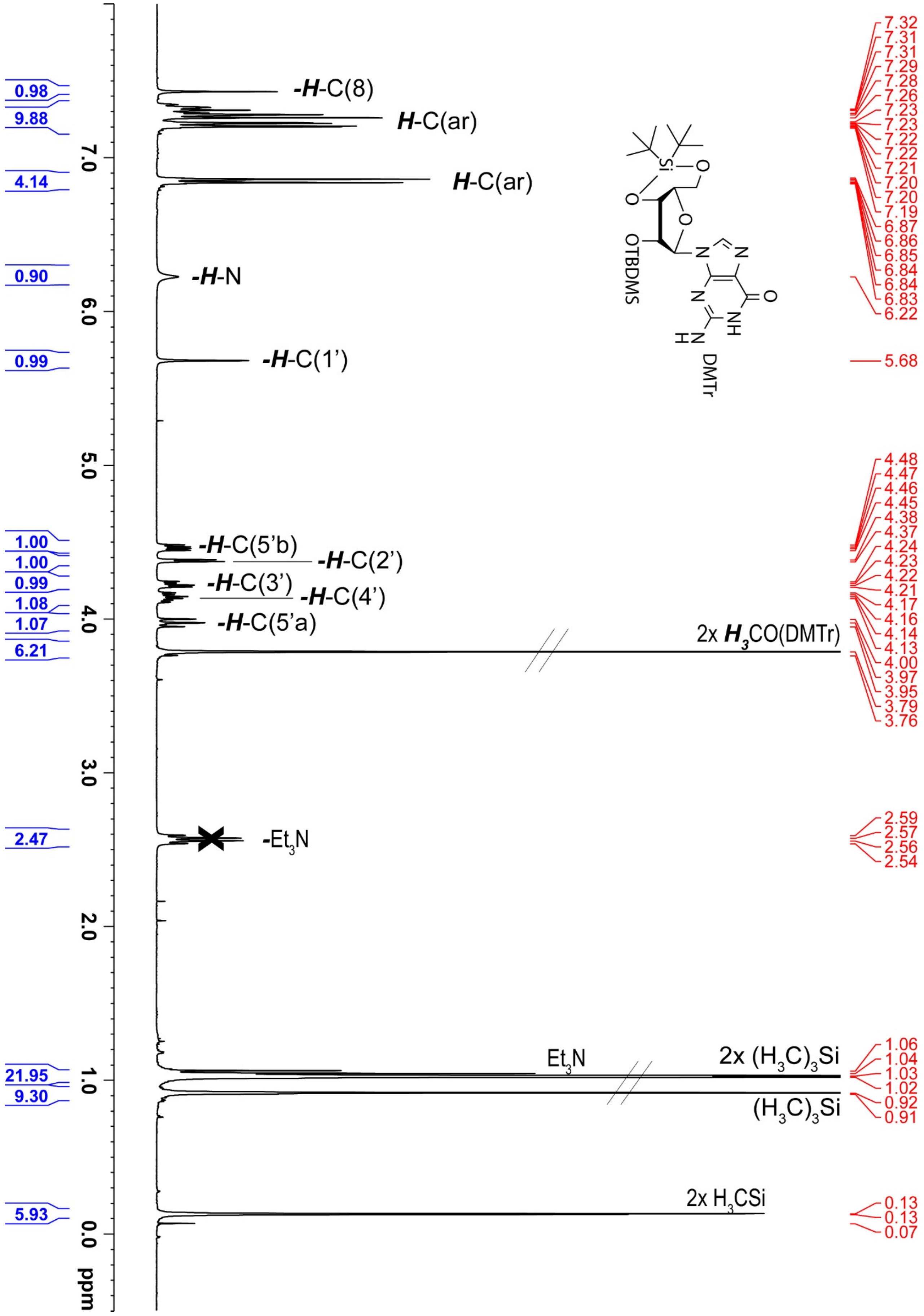

^{13}C-NMR (100 Mhz, $CDCl_3$, 25 °C) of compound **2**

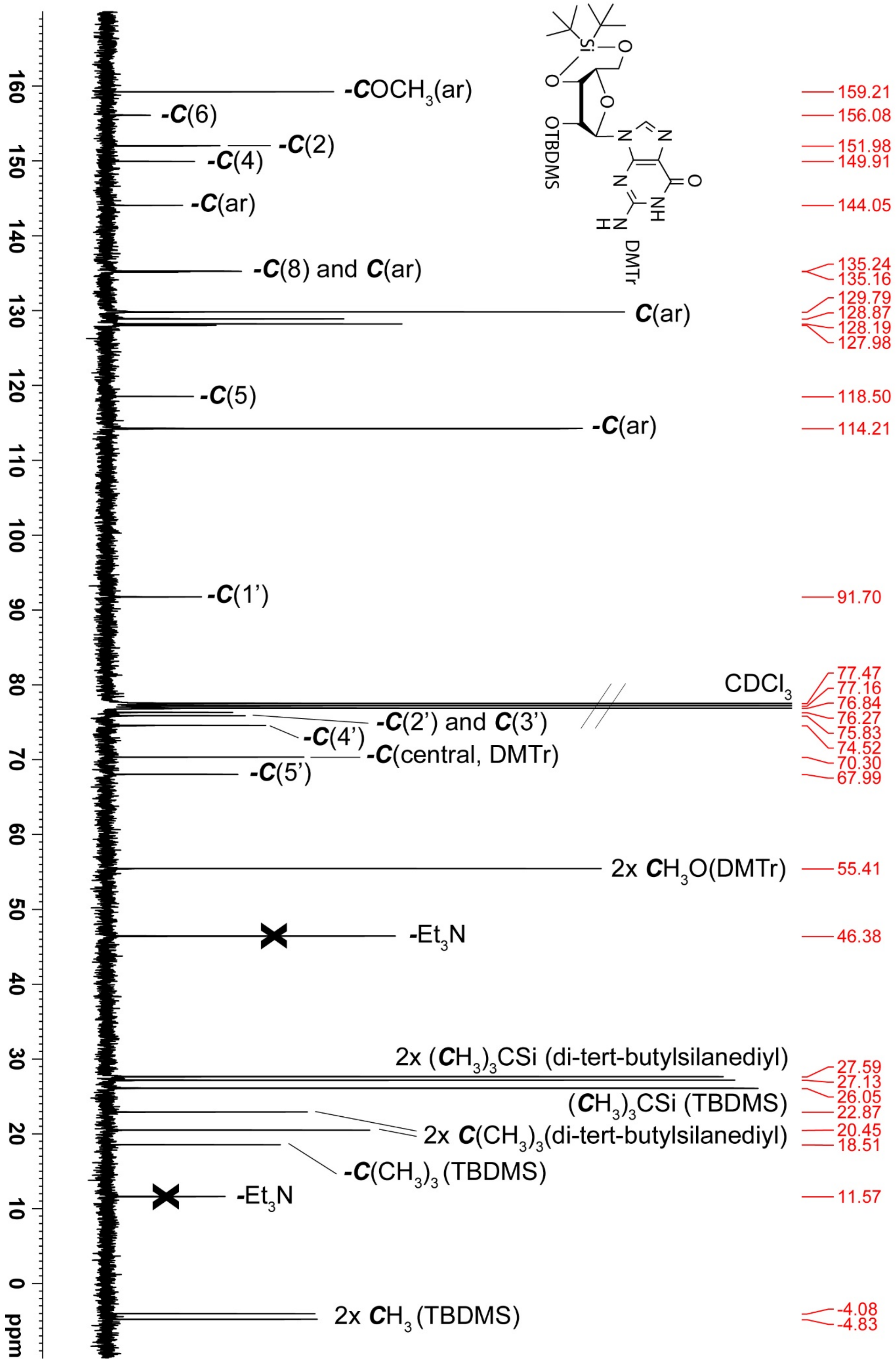

1.3. O^6-(4-Nitrophenyl)ethyl-N^2-(4,4′-dimethoxytrityl)-2'-*O*-(*tert*-butyldimethylsilyl)-3′,5′-*O*-(di-*tert*-butylsilanediyl)guanosine 3

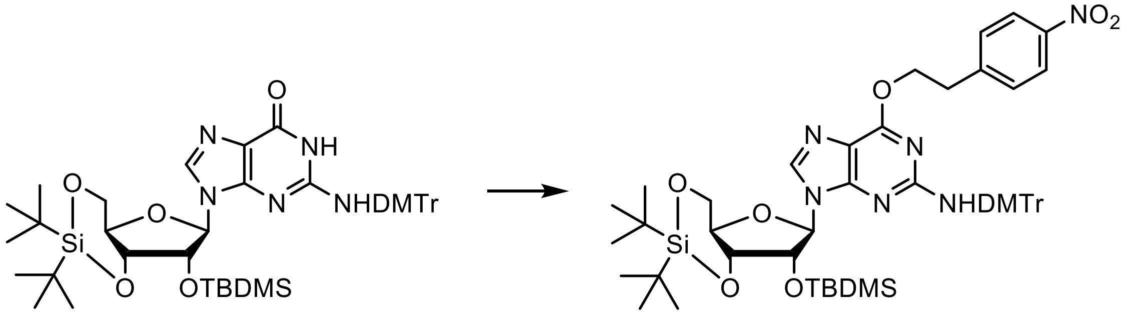

2 **3**

Triphenylphosphane (5.25 g, 20.0 mmol, 3.5 eq) and 2-(4-nitrophenyl)ethan-1-ol (3.34 g, 20.0 mmol, 3.5 eq) were dissolved in dioxane (50 mL). Compound **2** (4.80 g, 5.71 mmol) was added and the mixture was stirred for 10 minutes. Subsequently diisopropyl azodicarboxylate (4.04 g, 3.93 mL, 20.0 mmol, 3.5 eq) was added over the course of 5 min and the solution was stirred for 18 h at 60 °C. The solvent was evaporated and the crude product was purified via column chromatography (SiO_2, 0-50% dichloromethane in toluene). Yield: 5.65 g of compound **3** as a white foam (quantitative). TLC: 0.42 (3/7 ethyl acetate/cyclohexane). HR-ESI-MS (m/z): $[M+H]^+$ (calc): 989.4659; $[M+H]^+$ (meas): 989.4618. ^1H NMR (400 MHz, $CDCl_3$): δ 0.13 (s, 6H, 2x H_3CSi), 0.91 (s, 9H, $(H_3C)_3CSi$(TBDMS)), 0.99 (s, 9H, $(H_3C)_3CSi$), 1.01 (s, 9H, $(H_3C)_3CSi$), 2.81 (t, 2H, H_2CH_2CO (NPE), $^3J_{HH}$ = 7 Hz), 3.73 (s, 6H, H_3CO(DMTr)), 3.89 (brs, 2H, H_2CO (NPE)), 3.99 (t, 1H, H(4’), $^3J_{HH}$ = 10 Hz), 4.12-4.18 (m, 1H, H(5’a)), 4.43-4.49 (m, 3H, H(2’), H(3’) and H(5’b)), 5.74 (s, 1H, HNC(3)), 6.12 (s, 1H, HC(1’)), 6.72-6.74 (m, 4H, HC(ar, DMTr)), 7.04 (d, 2H, HC(ar, NPE), $^3J_{HH}$ = 8 Hz), 7.14-7.28 (m, 9H, HC(ar, DMTr)), 7.55 (s, 1H, HC(8)), 8.05 (d, 2H, HC(ar, NPE), $^3J_{HH}$ = 9 Hz), ppm. ^{13}C NMR (100 MHz, $CDCl_3$): δ -4.686 (s, 1C, CH_3Si(TBDMS)), -4.10 (s, 1C, CH_3Si(TBDMS)), 18.46 (s, 1C, $C(CH_3)_3$), 20.38 (s, 1C, $C(CH_3)_3$), 22.80 (s, 1C, $C(CH_3)_3$), 26.01 (s, 3C, $(CH_3)_3CSi$), 27.08 (s, 3C, $(CH_3)_3CSi$), 27.55 (s, 3C, $(CH_3)_3CSi$), 34.81 (s, 1C, CH_2CH_2O(NPE)), 55.26 (s, 2C, 2x CH_3O (DMTr)) 65.75 (s, 1C, CH_2O (NPE)), 67.95 (s, 1C, C(5’)), 70.25 (s, 1C, C(central DMTr)) 74.55 (s, 1C, C(4’)), 75.52 (s, 1C, C(2’)), 75.94 (s, 1C, C(3’)), 92.32 (s, 1C, C(1’)), 113.04 (s, 4C, C(ar), 115.79 (s, 1C, C(5)), 123.56-130.10 (m, 12C, CH(ar)), 137.65 (s, 1C, C(8)), 138.31 (s, 2C, C(ar)), 146.03 -146.70 (m, 4C, C(ar)), 153.01 (s, 1C, C(4)), 158.11-158.27 (m, 3C, C(2) and C(ar)), 159.56 (s, 1C, C(6)), ppm.

^{1}H-NMR (400 Mhz, $CDCl_3$, 25 °C) of compound **3**

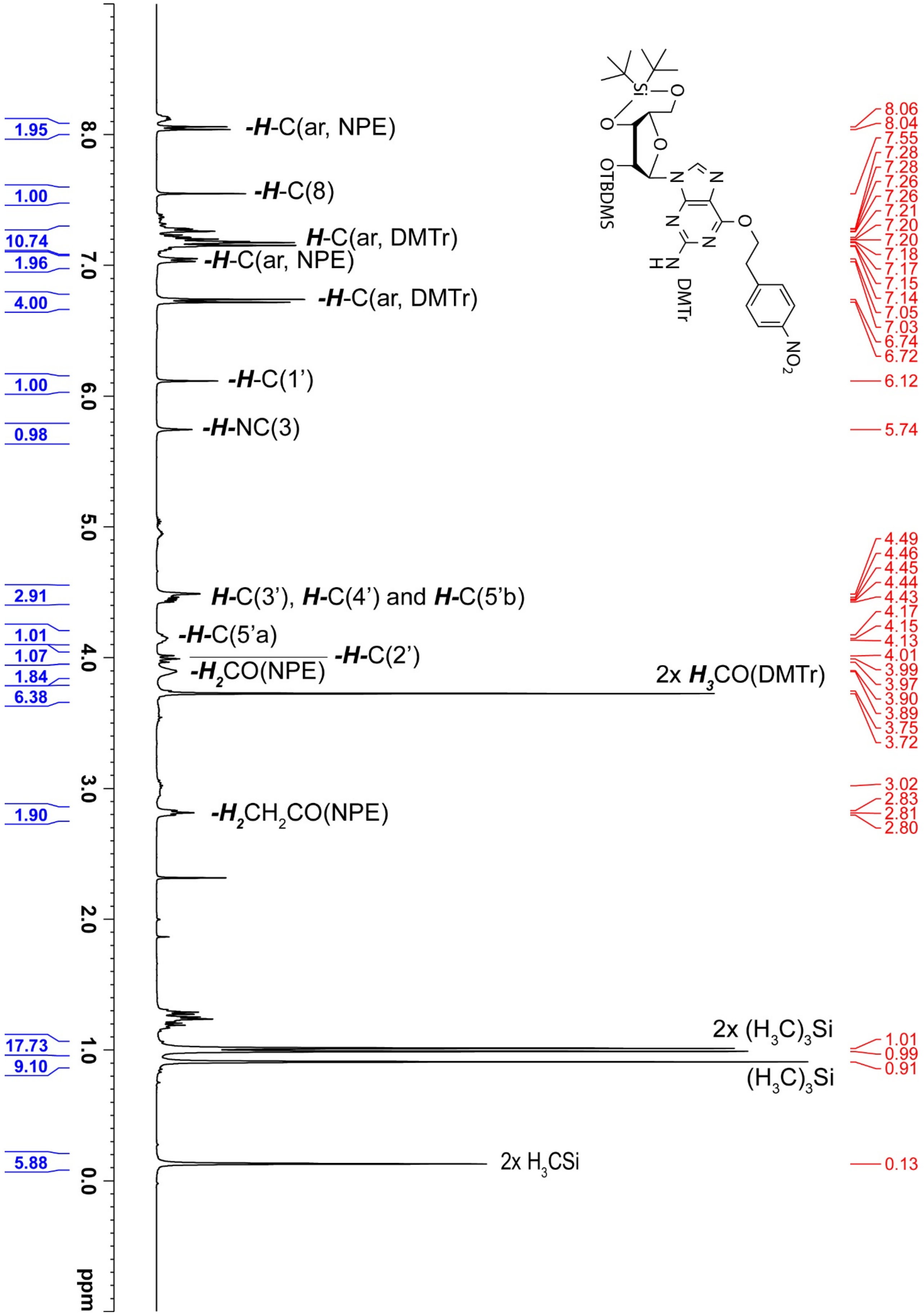

^{13}C-NMR (100 Mhz, $CDCl_3$, 25 °C) of compound **3**

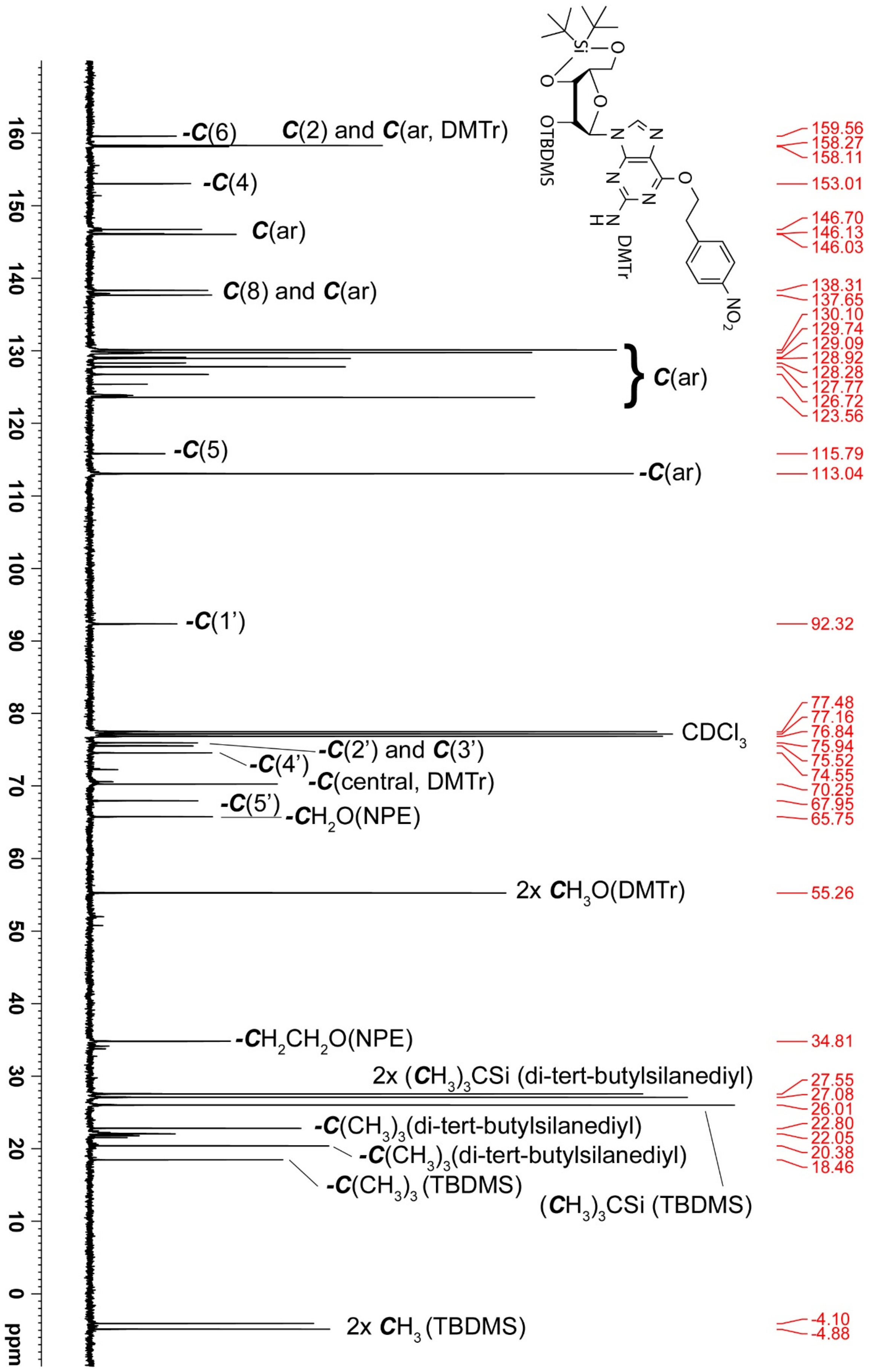

1.4. *O*[6]-(4-Nitrophenyl)ethyl-2′-*O*-(*tert*-butyldimethylsilyl)-3′,5′-*O*-(di-*tert*-butylsilanediyl)guanosine 4

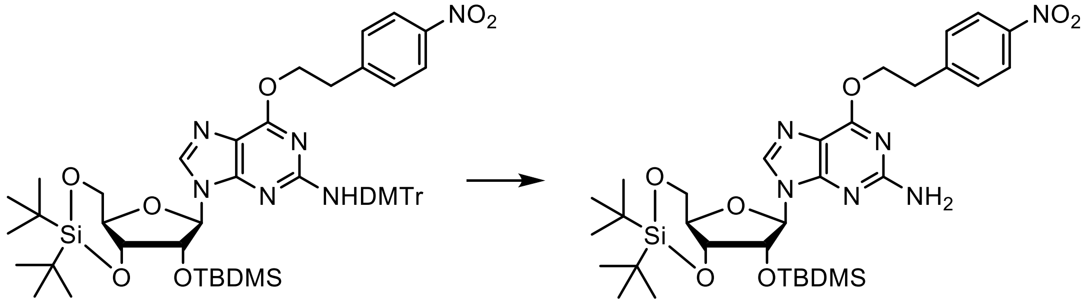

3 **4**

Compound **3** (5.65 g, 5.71 mmol) was dissolved in dichloromethane/methanol (9/1, 75 mL) at 0 °C and benzenesulfonic acid (3.00 g in dichloromethane methanol (9/1, 75 mL) was added. The solution went from colourless to orange immediately and the solution was stirred at 0 °C for 10 min. The solution was neutralized with sat. aqueous $NaHCO_3$ (100 mL) solution and stirred for 10 min. The layers were separated and the aqueous solution was extracted twice with dichloromethane (80 mL each). The combined organic phases were then washed with sat. aqueous $NaHCO_3$-solution and brine and dried over $MgSO_4$. The solvent was evaporated and the crude product was purified via column chromatography (SiO_2, 12-25% ethyl acetate in toluene). Yield: 3.65 g of compound **4** as a white solid (93%). TLC: 0.60 (4/96 methanol/dichloromethane). HR-ESI-MS (m/z): $[M+H]^+$ (calc): 687.3352; $[M+H]^+$ (meas): 687.3346. ^{1}H NMR (400 MHz, $CDCl_3$): δ 0.14 (s, 3H, 2x H_3CSi), 0.15 (s, 3H, 2x H_3CSi), 0.93 (s, 9H, $(H_3C)_3CSi$(TBDMS)), 1.03 (s, 9H, $(H_3C)_3CSi$), 1.07 (s, 9H, $(H_3C)_3CSi$), 3.27 (t, 2H, H_2CH_2CO (NPE)), $^3J_{HH}$ = 7 Hz), 4.01 (t, 1H, H(5'a), $^3J_{HH}$ = 10 Hz), 4.15-4.21 (m, 1H, H(4')), 4.44-4.51 (m, 3H, H(2'), H(3') and H(5'b)), 4.70-4.74 (m, 2H, H_2CO (NPE)), 5.78 (s, 1H, HC(1')), 7.48 (d, 2H, HC(ar, NPE), 9 Hz), 7.62 (s, 1H, HC(8)), 8.16 (d, 2H, HC(ar, NPE) 9 Hz), ppm. ^{13}C NMR (100 MHz, $CDCl_3$): δ -4.86 (s, 1C, CH_3Si(TBDMS)), -4.14 (s, 1C, CH_3Si(TBDMS)), 18.47 (s, 1C, $C(CH_3)_3$), 20.46 (s, 1C, $C(CH_3)_3$), 22.92 (s, 1C, $C(CH_3)_3$), 26.03 (s, 3C, $(CH_3)_3CSi$), 27.14 (s, 3C, $(CH_3)_3CSi$), 27.61 (s, 3C, $(CH_3)_3CSi$), 35.33 (s, 1C, CH_2CH_2O(NPE)), 66.34 (s, 1C, CH_2O (NPE)), 68.01 (s, 1C, C(5')), 74.68 (s, 1C, C(4')), 75.55 (s, 1C, C(2')), 75.97 (s, 1C, C(3')), 92.33 (s, 1C, C(1')), 116.30 (s, 1C, C(5)), 123.87 (s, 2C, CH(ar)), 130.05 (s, 2C, CH(ar)), 137.74 (s, 1C, C(8)), 146.10 (s, 1C, C(ar)), 146.99 (s, 1C, C(ar)), 153.30 (s, 1C, C(4)), 159.21 (s, 1C, C(2)), 161.02 (s, 1C, C(6)), ppm.

^{1}H-NMR (400 Mhz, $CDCl_3$, 25 °C) of compound **4**

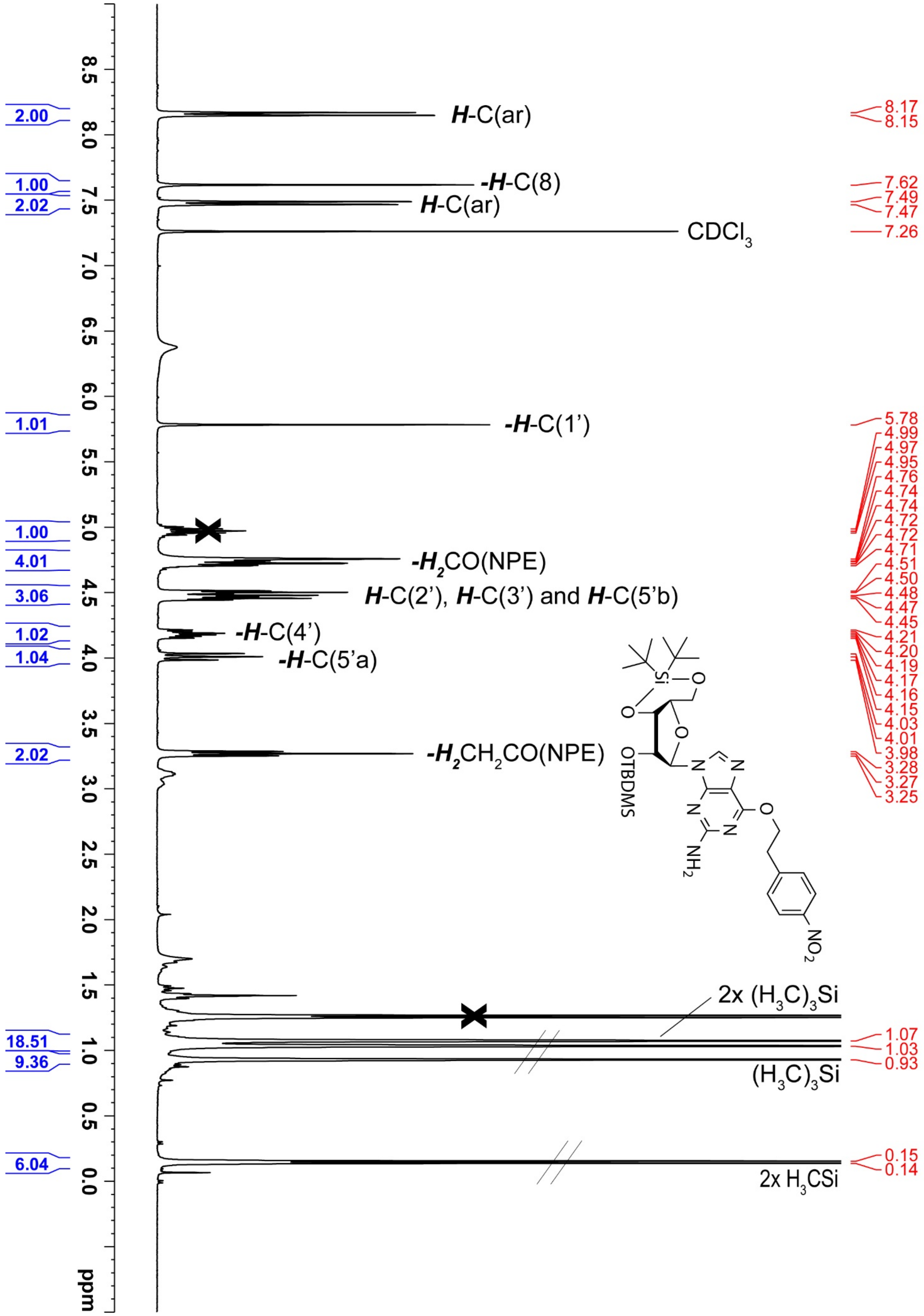

^{13}C-NMR (100 Mhz, $CDCl_3$, 25 °C) of compound **4**

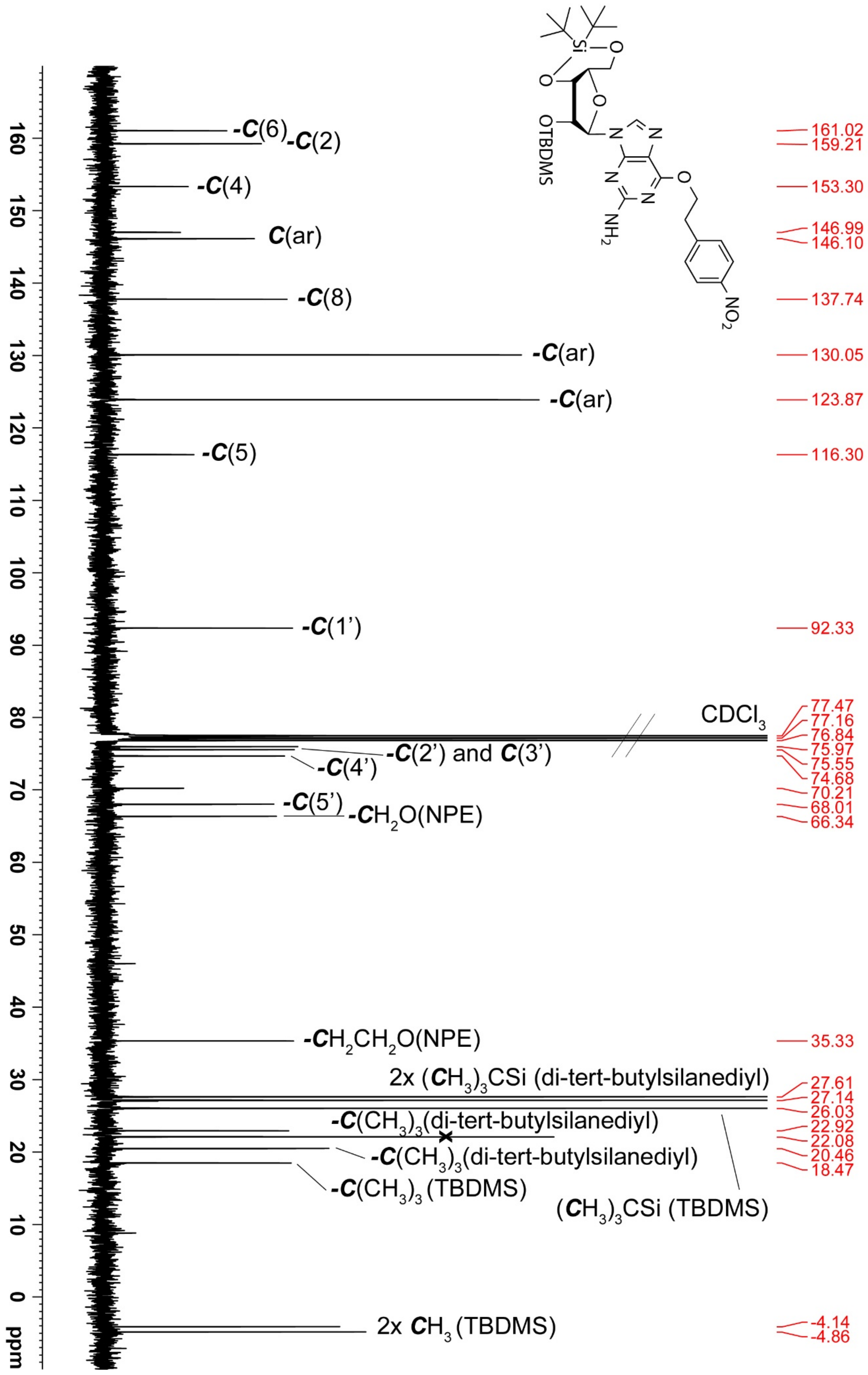

1.5. *O*[6]-(4-Nitrophenyl)ethyl-2′-*O*-(*tert*-butyldimethylsilyl)-3′,5′-*O*-(di-*tert*-butylsilanediyl)xanthosine 5

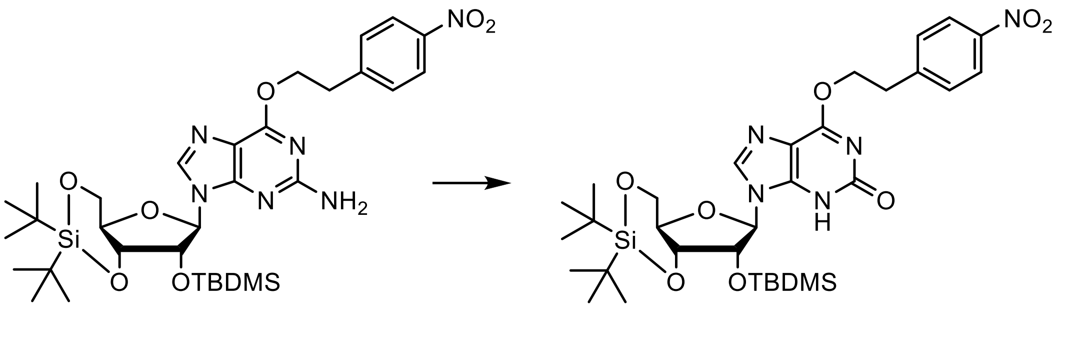

4 **5**

Compound **4** (3.65 g, 5.31 mmol) and sodium nitrite (12.0 g, 174 mmol, 33 eq) were dissolved in water/acetone (1/2, 60 mL). Acetic acid (18.4 g, 17.5 mL, 306 mmol, 58 eq) was added under vigorous stirring followed by intensive gas evolution. The solution was stirred for 24 h at room temperature. Subsequently the mixture was heated to 45 °C and stirred for another 2h. The mixture was neutralized with sat. $NaHCO_3$ solution (50 mL) and extracted three time with ethyl acetate (50 mL each). The combined organic phases were washed with sat. $NaHCO_3$ solution and brine and the solvent was removed. The crude product was further purified via column chromatography (SiO_2, 12-20% ethyl acetate in toluene). Yield: 2.35 g of compound **5** as a white solid (64%). TLC: 0.36 (4/96 methanol/dichloromethane). HR-ESI-MS (m/z): $[M+H]^+$ (calc): 688.3192; $[M+H]^+$ (meas): 688.3187. ^1H NMR (400 MHz, $CDCl_3$): δ 0.13 (s, 3H, H_3CSi), 0.14 (s, 3H, H_3CSi), 0.91 (s, 9H, $(H_3C)_3CSi$(TBDMS)), 1.04 (s, 9H, $(H_3C)_3CSi$), 1.07 (s, 9H, $(H_3C)_3CSi$), 3.30 (t, 2H, H_2CH_2CO (NPE)), $^3J_{HH}$ =7 Hz), 4.01 (t, 1H, H(5'a), $^3J_{HH}$ =10 Hz), 4.18-4.24 (m, 1H, H(4')), 4.28-4.31 (s, 1H, H(3')), 4.46-4.50 (m, 2H, H(2') and H(5'b)), 4.88 (t, 2H, H_2CO (NPE), $^3J_{HH}$ = 7 Hz), 5.90 (s, 1H, HC(1')), 7.50 (d, 2H, HC(ar, NPE), 9 Hz), 7.70 (s, 1H, HC(8)), 8.16-8.18 (m, 2H, HC(ar, NPE)), ppm. ^{13}C NMR (100 MHz, $CDCl_3$): δ -5.00 (s, 1C, CH_3Si(TBDMS)), -4.33 (s, 1C, CH_3Si(TBDMS)), 18.28 (s, 1C, $C(CH_3)_3$), 20.35 (s, 1C, $C(CH_3)_3$), 22.78 (s, 1C, $C(CH_3)_3$), 25.86 (s, 3C, $(CH_3)_3CSi$), 27.01 (s, 3C, $(CH_3)_3CSi$), 27.47 (s, 3C, $(CH_3)_3CSi$), 35.14 (s, 1C, CH_2CH_2O(NPE)), 67.77 (s, 1C, C(5')), 68.12 (s, 1C, CH_2O(NPE), 74.61 (s, 1C, C(4')), 75.76 (s, 1C, C(2')), 76.21 (s, 1C, C(3')), 91.75 (s, 1C, C(1')), 116.00 (s, 1C, C(5)), 123.77 (s, 2C, CH(ar)), 130.03 (s, 2C, CH(ar)), 138.18 (s, 1C, C(8)), 145.38 (s, 1C, C(ar)), 146.95 (s, 1C, C(ar)), 152.08 (s, 1C, C(4)), 159.87 (s, 1C, C(2)), 161.52 (s, 1C, C(6)), ppm.

^{1}H-NMR (400 Mhz, $CDCl_3$, 25 °C) of compound **5**

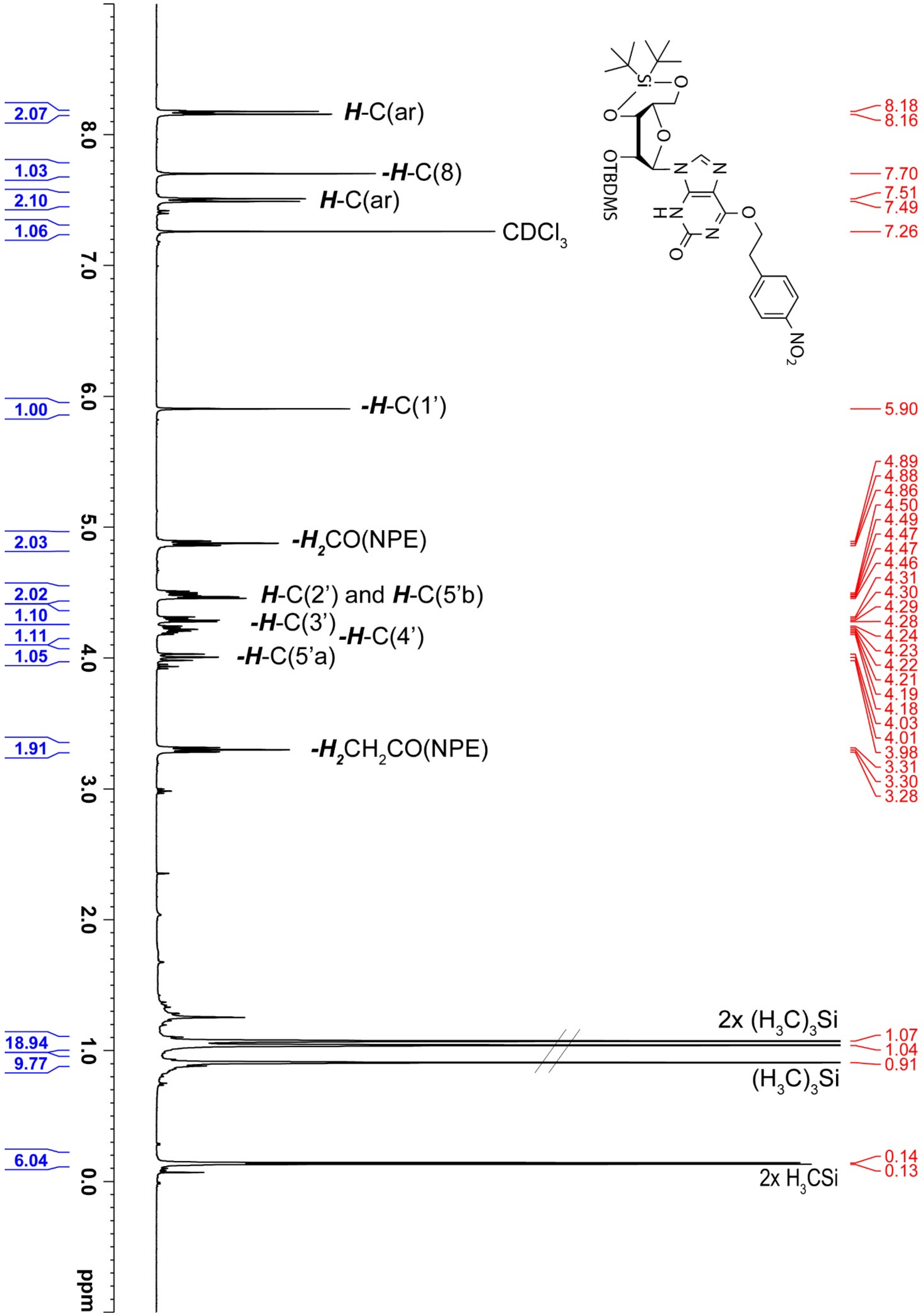

^{13}C-NMR (100 Mhz, $CDCl_3$, 25 °C) of compound **5**

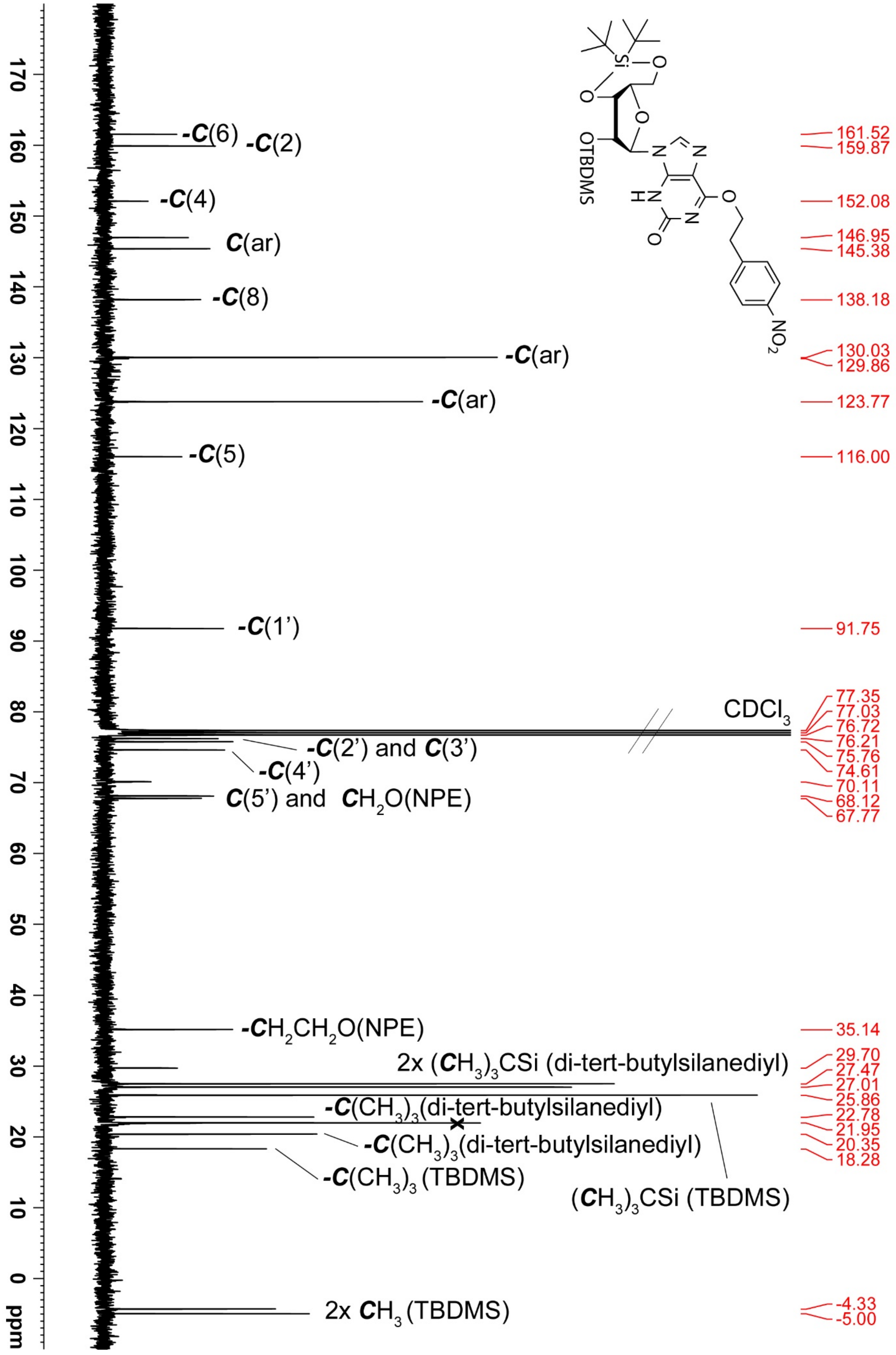

1.6. *O²,O⁶*-Bis(4-nitrophenyl)ethyl-2′-*O*-(*tert*-butyldimethylsilyl)-3′,5′-*O*-(di-*tert*-butylsilanediyl)xanthosine 6

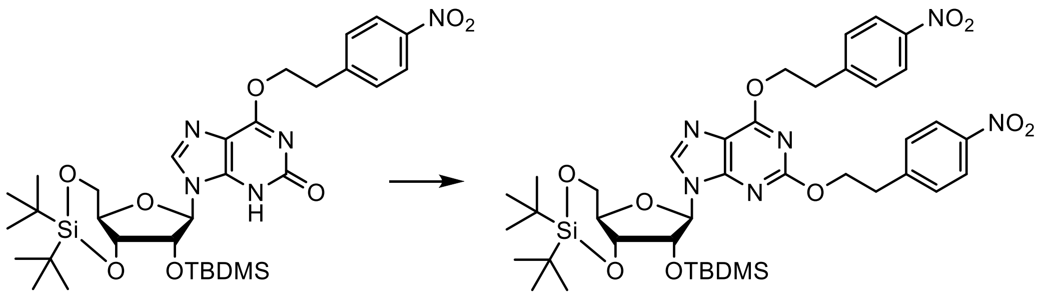

5 **6**

Compound **5** (2.35 mg, 3.42 mmol) was dissolved in anhydrous benzene (120 mL) and silver carbonate (1.41 g, 5.12 mmol, 1.5 eq) was added in one portion. The mixture was heated to reflux for one hour and subsequently *p*-nitrophenylethyl iodide (1.89 g, 6.83 mmol, 2 eq) was added. The reaction solution was stirred at 50 °C for 15 h and filtrated. The solvent was evaporated and the crude product purified via column chromatography (SiO_2, 10-20% ethyl acetate in toluene). Yield: 2.46 g of compound **6** as a white foam (86%). TLC: 0.26 (3/7 ethyl acetate/cyclohexane). HR-ESI-MS (m/z): $[M+H]^+$ (calc): 837.3669; $[M+H]^+$ (meas): 837.3639. ^1H NMR (400 MHz, $CDCl_3$): δ 0.08 (s, 3H, H_3CSi), 0.11 (s, 3H, H_3CSi), 0.87 (s, 9H, $(H_3C)_3CSi$(TBDMS)), 1.04 (s, 9H, $(H_3C)_3CSi$), 1.04 (s, 9H, $(H_3C)_3CSi$), 3.23 (t, 2H, H_2CH_2CO (NPE), $^3J_{HH}$ =7 Hz), 3.29 (t, 2H, H_2CH_2CO (NPE), 7 Hz), 3.99 (t, 1H, H(5’a), 10 Hz), 4.16-4.21 (m, 1H, H(4’)), 4.28-4.31 (m, 1H, H(3’)), 4.46-4.50 (m, 1H, H(5’b)), 4.52 -4.55 (m, 1H, HC(2’)), 4.59-4.64 (m, 2H, H_2CO (NPE)), 4.79 (t, 2H, H_2CO (NPE), $^3J_{HH}$ =7 Hz), 5.89 (s, 1H, HC(1’)), 7.47 (d, 2H, HC(ar, NPE), $^3J_{HH}$ =8 Hz), 7.76 (s, 1H, HC(8)), 8.16 (t, 2H, HC(ar, NPE), $^3J_{HH}$ =9 Hz), ppm. ^{13}C NMR (100 MHz, $CDCl_3$): δ -4.90 (s, 1C, CH_3Si(TBDMS)), -4.13 (s, 1C, CH_3Si(TBDMS)), 18.43 (s, 1C, $C(CH_3)_3$), 20.49 (s, 1C, $C(CH_3)_3$), 22.82 (s, 1C, $C(CH_3)_3$), 25.95 (s, 3C, $(CH_3)_3CSi$), 27.13 (s, 3C, $(CH_3)_3CSi$), 27.56 (s, 3C, $(CH_3)_3CSi$), 35.21 (s, 1C, CH_2CH_2O(NPE)), 35.28 (s, 1C, CH_2CH_2O(NPE)), 66.96 (s, 1C, CH_2O(NPE), 67.55 (s, 1C, C(5’)), 67.83 (s, 1C, CH_2O(NPE), 74.58 (s, 1C, C(4’)), 75.55 (s, 1C, C(2’)), 76.41 (s, 1C, C(3’)), 92.02 (s, 1C, C(1’)), 118.02 (s, 1C, C(5)), 123.88 (s, 4C, CH(ar)), 129.99 (s, 2C, CH(ar)), 130.08 (s, 2C, CH(ar)), 139.30 (s, 1C, C(8)), 145.77-147.04 (m, 4C, C(ar)), 152.68 (s, 1C, C(4)), 161.09 (s, 1C, C(2)), 161.68 (s, 1C, C(6)), ppm;

^{1}H-NMR (400 Mhz, $CDCl_3$, 25 °C) of compound **6**

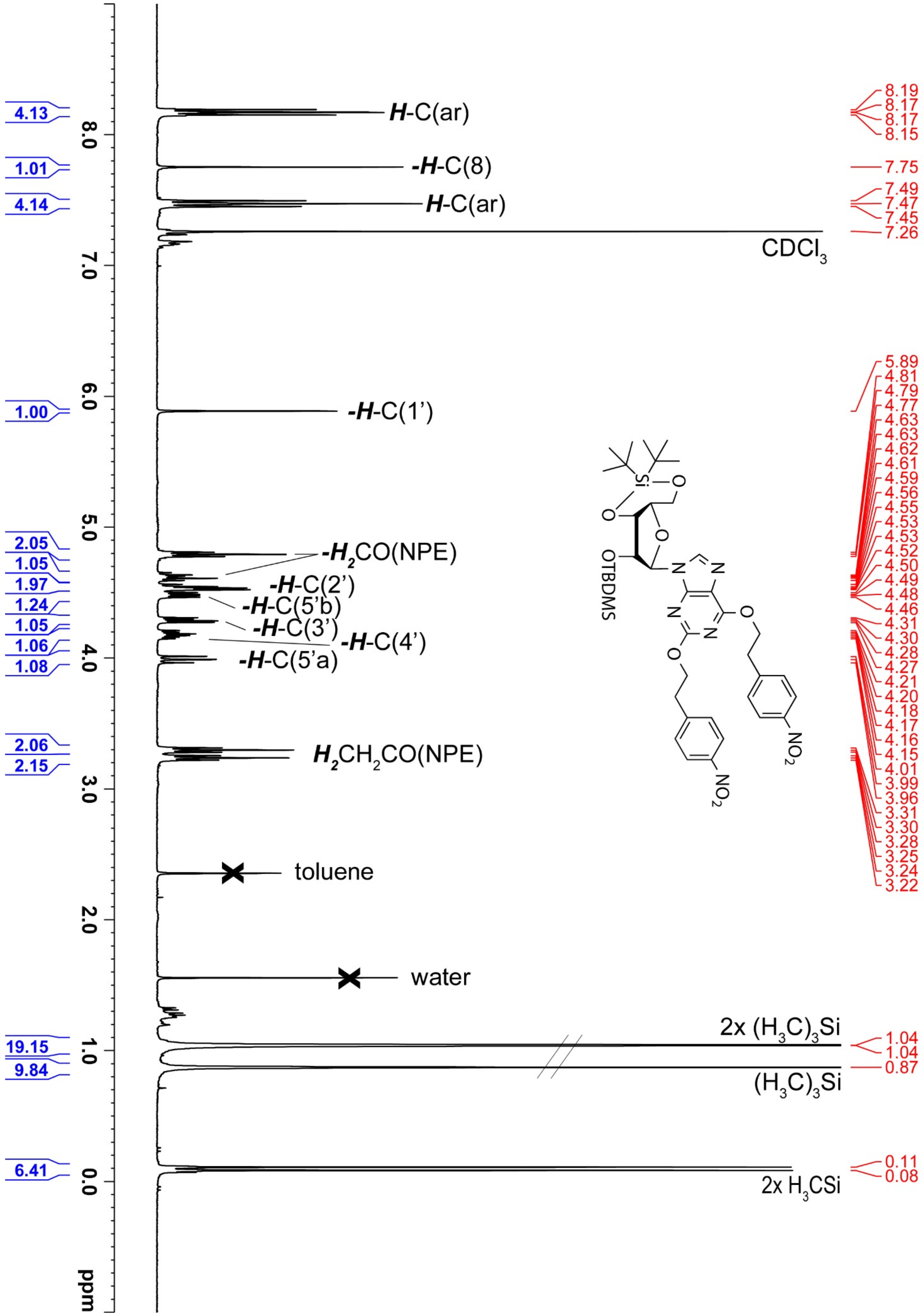

^{13}C-NMR (100 Mhz, $CDCl_3$, 25 °C) of compound **6**

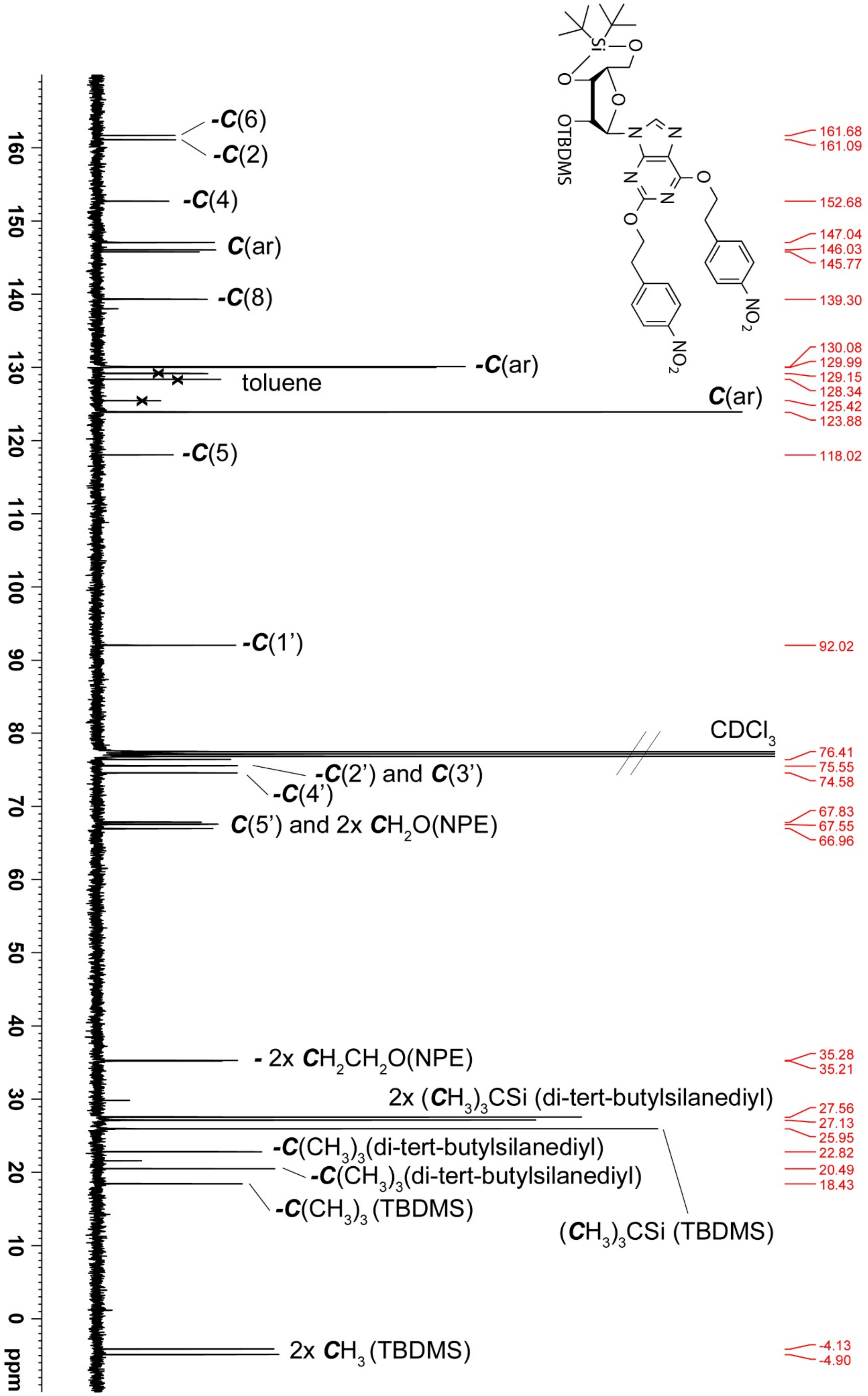

1.7. O^2,O^6-Bis(4-nitrophenyl)ethyl-2′-*O*-(*tert*-butyldimethylsilyl)xanthosine 7

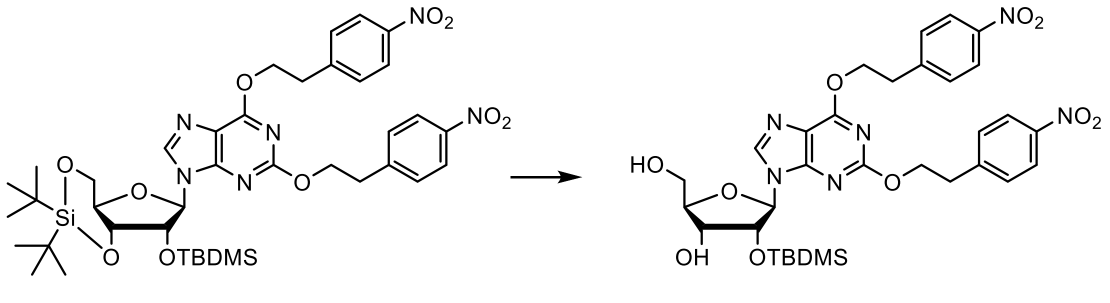

6 **7**

Compound **6** (2.46 g, 2.90 mmol) was dissolved in dichloromethane (15 mL) and cooled to 0 °C. HF pyridine (0.726 mL of a 8 M solution diluted with 5 mL pyridine) was added and the mixture was stirred for 2 h at 0 °C. Subsequently the reaction solution was quenched with sat. $NaHCO_3$ solution (20 mL) and diluted with dichloromethane (20 mL). The organic layer was washed with brine, dried over $MgSO_4$ and evaporated to dryness. The crude product was purified via column chromatography (SiO_2, 0-3% methanol in dichloromethane). Yield: 1.84 g of compound **7** as a white solid (91%). TLC: 0.22 (4/96 methanol/dichloromethane). HR-ESI-MS (m/z): $[M+H]^+$ (calc): 697.2648; $[M+H]^+$ (meas): 697.2618. ^1H NMR (400 MHz, DMSO): δ -0.22 (s, 3H, H_3CSi), -0.09 (s, 3H, H_3CSi), 0.69 (s, 9H, $(H_3C)_3CSi$(TBDMS)), 3.22 (t, 2H, H_2CH_2CO (NPE), $^3J_{HH}$ =7 Hz), 3.27 (t, 2H, H_2CH_2CO (NPE), $^3J_{HH}$ = 7 Hz), 3.57-3.72 (m, 2H, H(5’)), 3.94-3.97 (m, 1H, H(4’)), 4.11-4.15 (m, 1H, H(3’)), 4.56-4.59 (m, 3H, H(2’) and H_2CO(NPE)), 4.77 (t, 2H, H_2CO(NPE), $^3J_{HH}$ =7 Hz), 5.11-5.12 (m, 2H, HOC(3’) and HOC(5’)), 5.88 (d, 1H, HC(1’), $^3J_{HH}$ = 6 Hz), 7.59-7.62 (m, 4H, HC(ar, NPE)), 8.15-8.18 (m, 4H, HC(ar, NPE)), 8.42 (s, 1H, HC(8)), ppm. ^{13}C NMR (100 MHz, $CDCl_3$): δ -5.42 (s, 1C, CH_3Si(TBDMS)), -4.92 (s, 1C, CH_3Si(TBDMS)), 17.71 (s, 1C, $C(CH_3)_3$), 25.44 (s, 3C, $(CH_3)_3CSi$), 34.20 (s, 1C, CH_2CH_2O(NPE)), 34.24 (s, 1C, CH_2CH_2O(NPE)), 60.94 (s, 1C, C(5’)), 66.40 (s, 1C, CH_2O(NPE), 67.08 (s, 1C, CH_2O(NPE), 69.96 (s, 1C, C(3’)), 75.74 (s, 1C, C(2’)), 85.53 (s, 1C, C(4’)), 87.51 (s, 1C, C(1’)), 118.73 (s, 1C, C(5)), 123.39 (s, 2C, CH(ar)), 123.41 (s, 2C, CH(ar)), 130.25 (s, 2C, CH(ar)), 130.27 (s, 2C, CH(ar)), 140.62 (s, 1C, C(8)), 146.24-146.82 (m, 4C, C(ar)), 153.19 (s, 1C, C(4)), 160.16 (s, 1C, C(2)), 160.72 (s, 1C, C(6)), ppm;

^{1}H-NMR (400 Mhz, DMSO-d6, 25 °C) of compound **7**

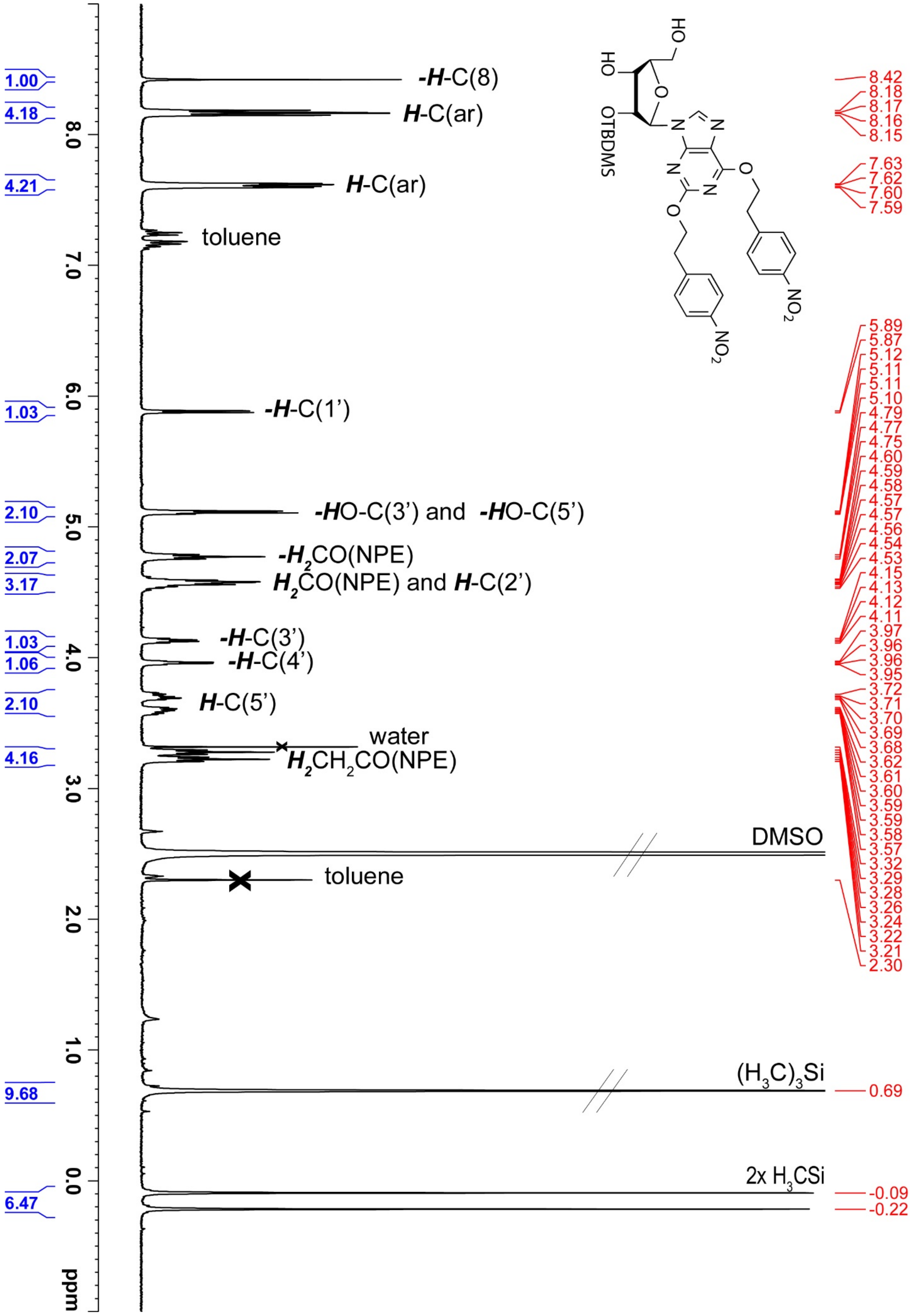

^{13}C-NMR (100 Mhz, DMSO-d6, 25 °C) of compound **7**

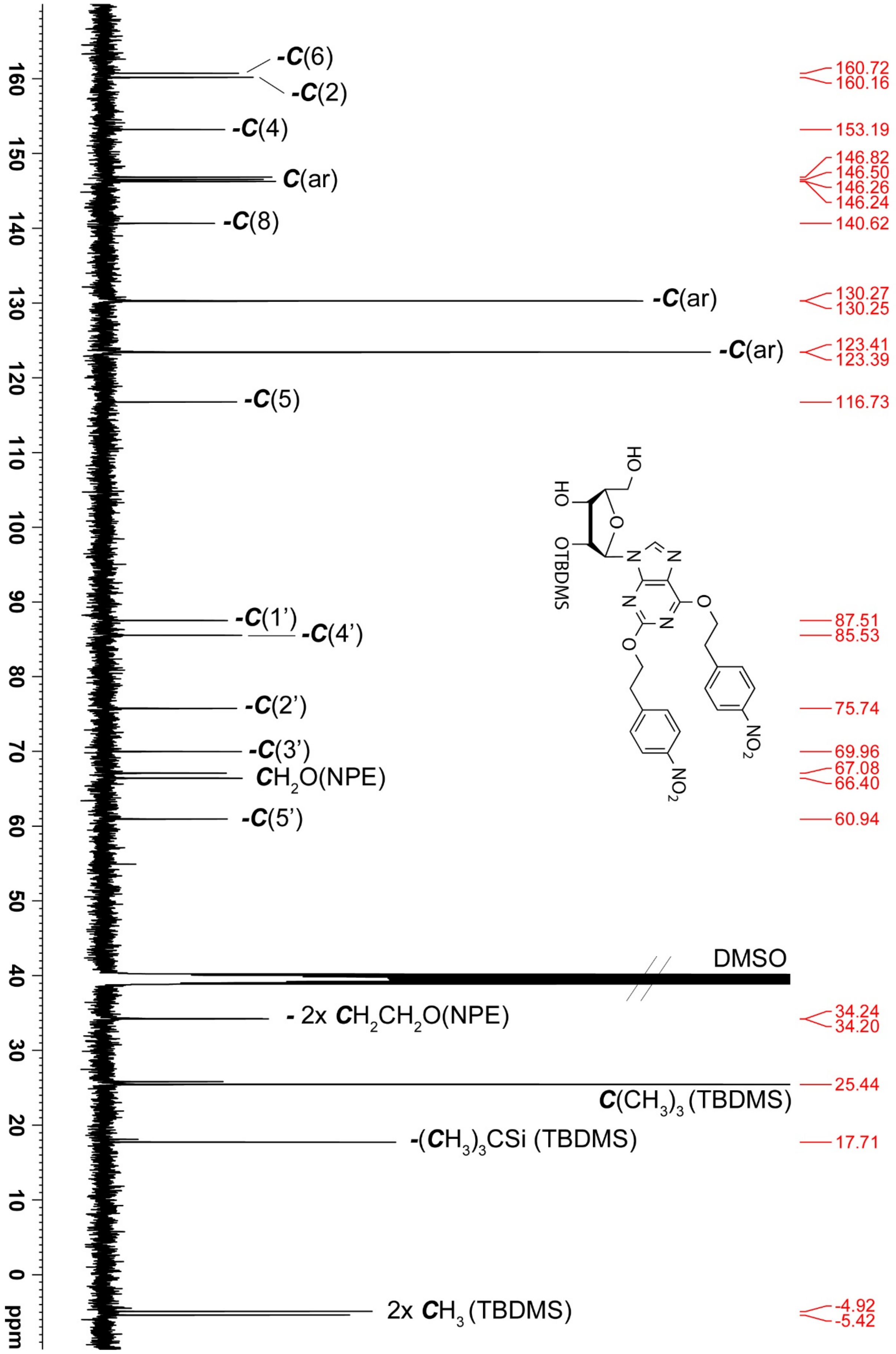

1.8. O^2,O^6-Bis(4-nitrophenyl)ethyl-5′-*O*-(4,4′-dimethoxytrityl)-2′-*O*-(*tert*-butyldimethylsilyl)xanthosine 8

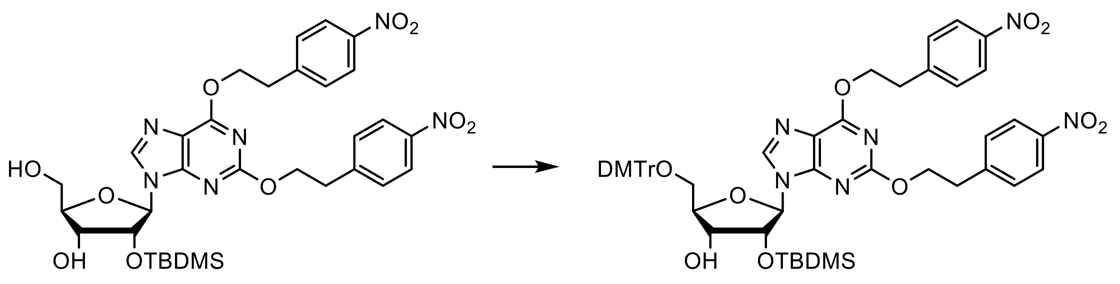

7 **8**

Compound **7** (1.84 g, 2.64 mmol) was dissolved in anhydrous pyridine (20 mL) and DMTrCl (1.16 g, 3.43 mmol, 1.3 eq) was added in two portions over the course of 30 min. The reaction solution was stirred at room temperature for 3 h. The solvent was evaporated and the residue was taken up in ethyl acetate. The solution was washed twice with 5% citric acid, sat. $NaHCO_3$, brine and then dried over $MgSO_4$. The solvent was removed and the crude product purified via column chromatography (SiO_2, 0-2% methanol in dichloromethane). Yield: 2.51 g of compound **8** as a white foam (95%). TLC: 0.75 (4/96 methanol/dichloromethane). HR-ESI-MS (m/z): $[M+H]^+$ (calc): 999.3955; $[M+H]^+$ (meas): 999.3910. ^{1}H NMR (400 MHz, DMSO): δ -0.19 (s, 3H, H_3CSi), 0.00 (s, 3H, H_3CSi), 0.83 (s, 9H, $(H_3C)_3CSi$(TBDMS)), 3.17 (t, 2H, H_2CH_2CO (NPE), $^3J_{HH}$ = 7 Hz), 3.31 (t, 2H, H_2CH_2CO (NPE), $^3J_{HH}$ =7 Hz), 3.37-3.50 (m, 2H, H(5')), 3.78 (s, 3H, H_3CO(DMT)), 3.79 (s, 3H, H_3CO(DMT)), 4.22-4.24 (m, 1H, H(4')), 4.35-4.38 (m, 1H, H(3')), 4.47-4.58 (m, 2H, H_2CO(NPE)), 4.78-4.84 (m, 3H, H(2') and H_2CO(NPE)), 6.02 (d, 1H, HC(1'), $^3J_{HH}$ =6 Hz), 6.79-6.82 (m, 4H, HC(ar)), 7.16-7.50 (m, 8H, HC(ar)), 8.01 (s, 1H, HC(8)), 8.14-8.18 (m, 8H, HC(ar)), 8.61-8.64 (m, 4H, HC(ar)), ppm. ^{13}C NMR (100 MHz, $CDCl_3$): δ -5.00 (s, 1C, CH_3Si(TBDMS)), -4.84 (s, 1C, CH_3Si(TBDMS)), 18.00 (s, 1C, $C(CH_3)_3$), 25.64 (s, 3C, $(CH_3)_3CSi$), 35.20 (s, 1C, CH_2CH_2O(NPE)), 35.25 (s, 1C, CH_2CH_2O(NPE)), 55.36 (s, 2C, CH_3O(DMTr)), 63.67 (s, 1C, C(5')), 66.92 (s, 1C, CH_2O(NPE), 67.50 (s, 1C, CH_2O(NPE), 71.80 (s, 1C, C(3')), 76.40 (s, 1C, C(2')), 84.20 (s, 1C, C(4')), 86.89 (s, 1C, C(central, DMTr)). 87.43 (s, 1C, C(1')), 113.31 (s, 2C, CH(ar)), 113.40 (s, 2C, CH(ar)), 117.65 (s, 1C, C(5)), 123.87-136.06 (s, 19C, CH(ar)), 139.48 (s, 1C, C(8)), 144.57-150.02 (m, 5C, C(ar)), 153.67 (s, 1C, C(4)), 158.77 (s, 2C, CH(ar)), 161.07 (s, 1C, C(2)), 161.64 (s, 1C, C(6)), ppm;

^{1}H-NMR (400 Mhz, $CDCl_3$, 25 °C) of compound **8**

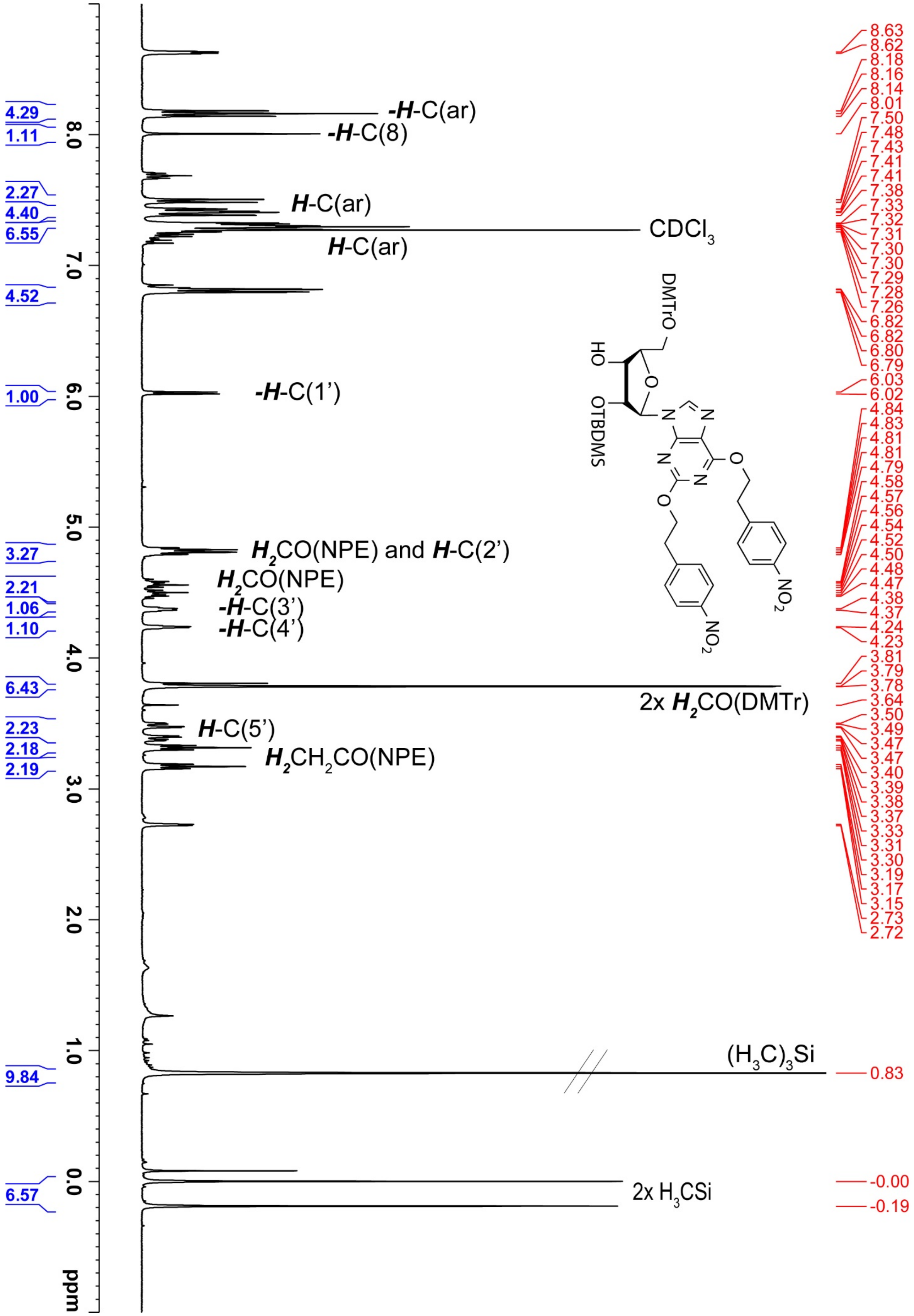

^{13}C-NMR (100 Mhz, CDCl3, 25 °C) of compound **8**

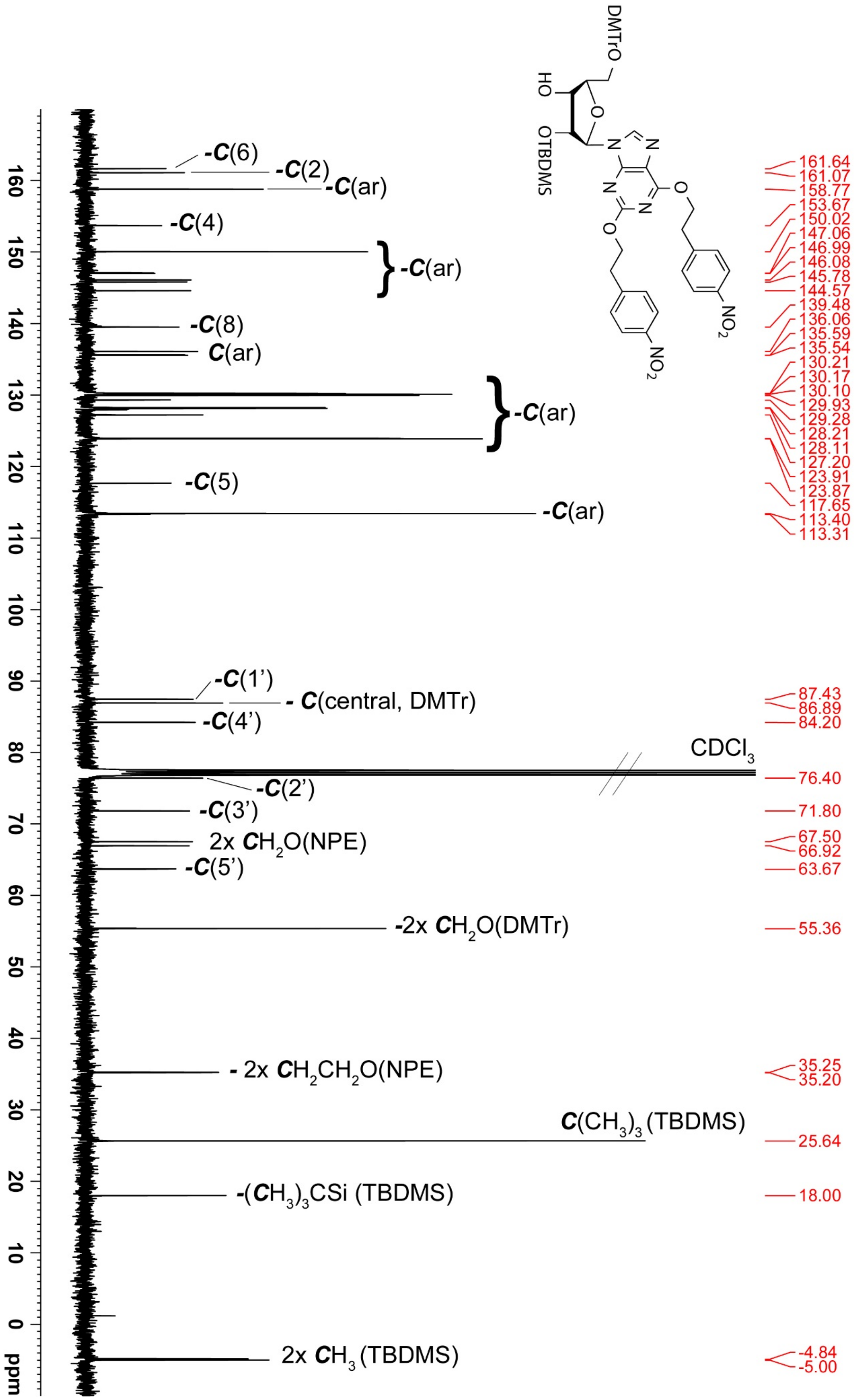

1.9. *O*[2],*O*[6]-Bis(4-nitrophenyl)ethyl-5′-*O*-(4,4′-dimethoxytrityl)-2′-*O*-(*tert* butyldimethylsilyl)xanthosine-3′-*O*-(2-cyanoethyl) *N*,*N*-diisopropylphosphoramidite

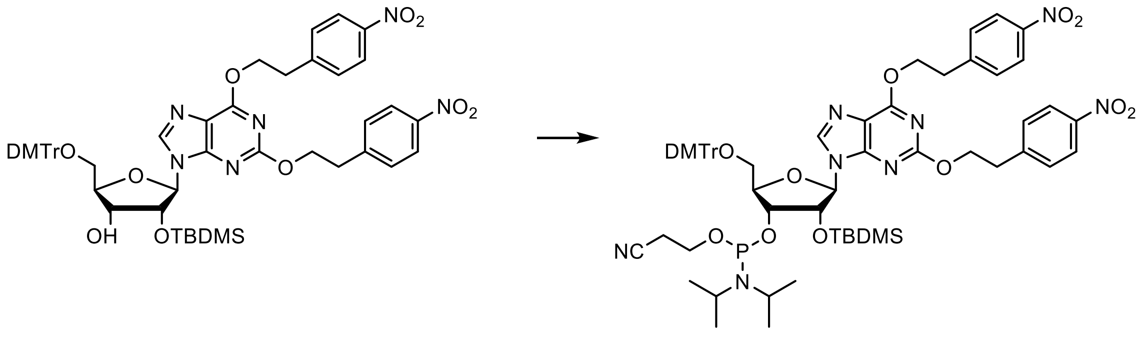

8 **9**

Compound **8** (1.00 g, 1.00 mmol) was coevaporated three times each with pyridine, toluene and dichloromethane. It was then dissolved in tetrahydrofurane (10 mL) and 1-methylimidazole (49 mg, 0.60 mmol, 0.6 eq) and 2,4,6-trimethylpyridine (849 mg, 7.01 mmol, 7 eq) was added. 2-cyanoethyl N,N-diisopropylchlorophosphoramidite (592 mg, 2.50 mmol, 2.5 eq) was added dropwise and the solution was stirred at room temperature for 2 h. The mixture was diluted with dichloromethane and washed with sat. $NaHCO_3$ solution and brine and dried over $MgSO_4$. The solvent was evaporated and the crude product was purified via column chromatography (SiO_2, 2-5% methanol in dichloromethane). Yield: 850 mg of compound **9** as a white foam (71%). HR-ESI-MS (mz): $[M+H]^+$ (calc): 1199.5033; $[M+H]^+$ (meas): 1199.4992. ^{1}H NMR (400 MHz, $CDCl_3$): δ -0.23 and -0.21 (s, 3H, H_3CSi), -0.03 (s, 3H, H_3CSi), 0.73 and 0.75 (s, 9H, $(H_3C)_3CSi$(TBDMS)), 1.14-1.16 (m, 12H, $(H_3C)_2CH$(*N,N*-diisopropyl)), 2.26-2.28 and 2.60-2.61 (m, 2H,H_2C(cyanoethyl)), 3.11-3.16 (m, 2H, H_2CH_2CO (NPE)), 3.29-3.32 (m, 3H, H_2CH_2CO(NPE) and Hb-C(5')), 3.55-3.59 (m, 4H, Ha-C(5'), Hb-C(Cyanoethyl) and 2x *H*C$(CH_3)_2$(*N,N*-diisopropyl)), 3.83-3.95 (m, 1H, Ha-C(cyanoethyl)), 4.30-4.59 (m, 4H, HC(3'), HC(4') and H_2CO(NPE)), 4.79-4.80 (m, 3H, HC(2') and H_2CO(NPE)), 5.99 - 6.01 and 6.06 - 6.07 (m, 1H HC(1')), 6.79-6.82 (m, 4H, HC(ar)), 7.20-7.50 (m, 8H, HC(ar), 8.03 and 8.06 (m, 1H, HC(8)), 8.12-8.17 (m, 4H, HC(ar)), ppm. ^{13}C NMR (100 MHz, $CDCl_3$): δ -5.03 and -4.53 (s, 2C, CH_3Si(TBDMS)), 17.97 and 18.05 (s, 1C, $C(CH_3)_3$), 20.17 and 20.24 (s, 1C, C(cyanoethyl)), 24.73 (s, 4C, 4x CH_3(*N,N*-diisopropyl) 25.66 and 25.70 (s, 3C, $(CH_3)_3CSi$), 35.15 (s, 1C, CH_2CH_2O(NPE)), 35.25 (s, 1C, CH_2CH_2O(NPE)), 43.48 and 43.61 (s, 2C, 2x $CH(CH_3)$ (*N,N*-diisopropyl)), 55.37 (s, 2C, CH_3O(DMTr)), 55.51 and 55.71 (s, 1C, C(cyanoethyl)), 63.47 (s, 1C, C(5')), 66.85 and 67.35 (s, 1C, CH_2O(NPE), 67.50 (s, 1C, CH_2O(NPE), 73.68 and 73.78 (s, 1C, C(3')), 75.39 and 75.77 (s, 1C, C(2')), 84.26 (s, 1C, C(4')), 86.80 (s, 1C, C(central, DMTr)). 87.18 (s, 1C, C(1')), 113.38 (s, 2C, CH(ar)), 113.42 (s, 2C, CH(ar)), 117.36 and 117.74 (s, 1C, C(5)), 123.82-135.70 (s, 19C, CH(ar)), 139.59 (s, 1C, C(8)), 144.46-147.03 (m, 5C, C(ar)), 153.79 (s, 1C, C(4)), 158.77 (s, 2C, CH(ar)), 160.93 (s, 1C, C(2)), 161.59 (s, 1C, C(6)), ppm. ^{31}P NMR (162 MHz, $CDCl_3$): δ 149.29 (s, 1P), 151.13 (s, 1P), ppm;

^{1}H-NMR (400 MHz, CDCl3, 25 °C) of compound **9**

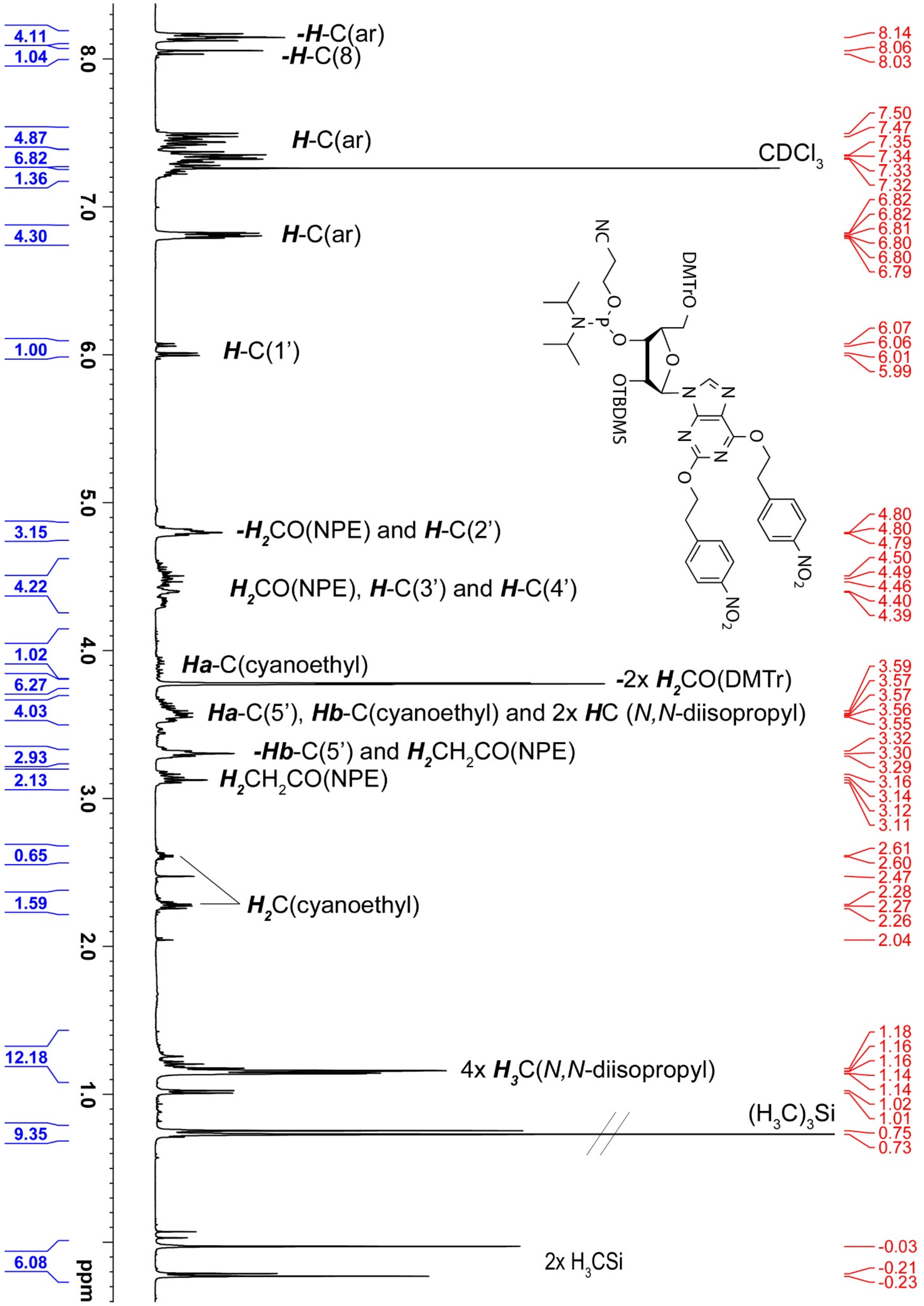

^{13}C-NMR (100 MHz, $CDCl_3$, 25 °C) of compound **9**

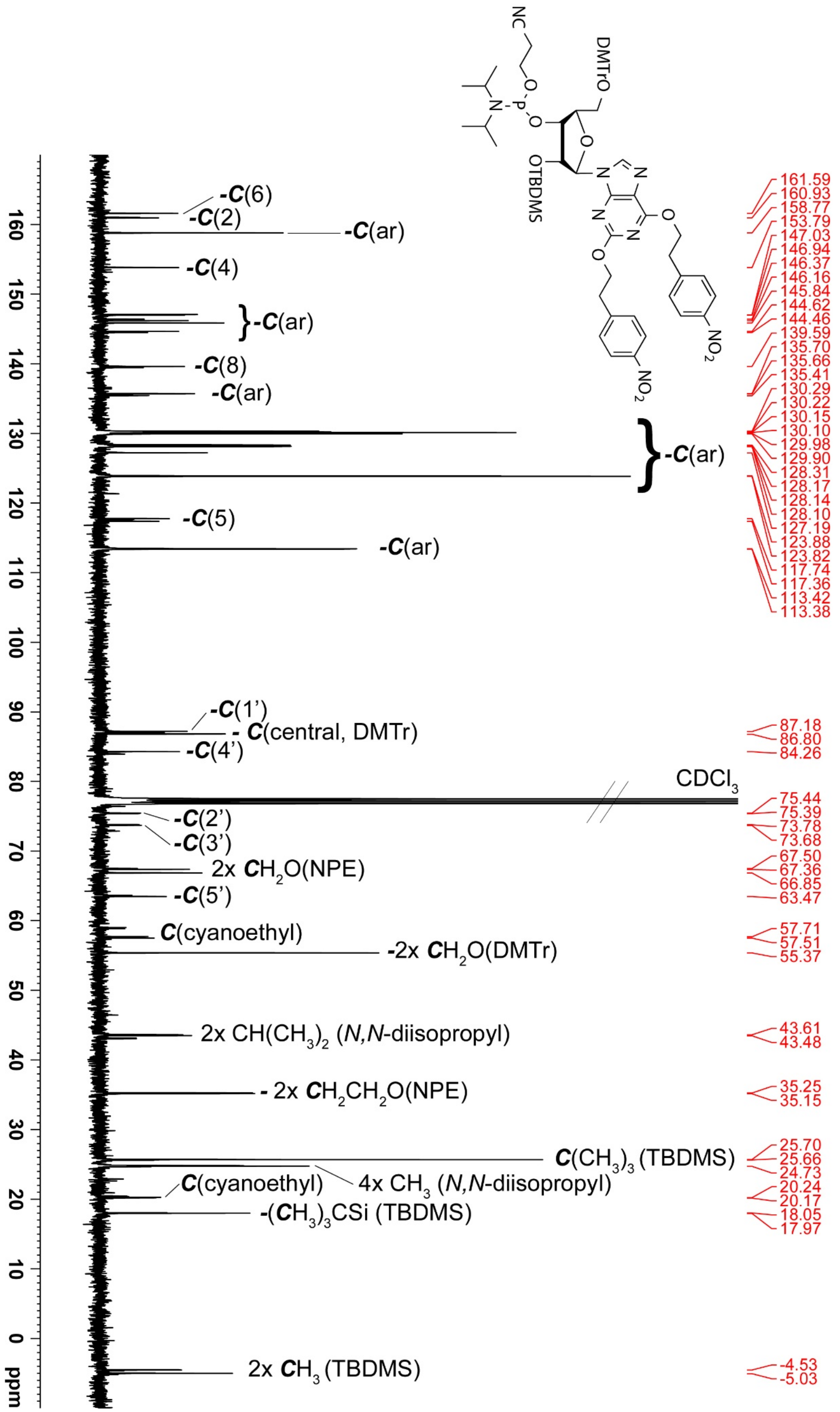

^{31}P-NMR (162 MHz, $CDCl_3$, 25 °C) of compound **1**

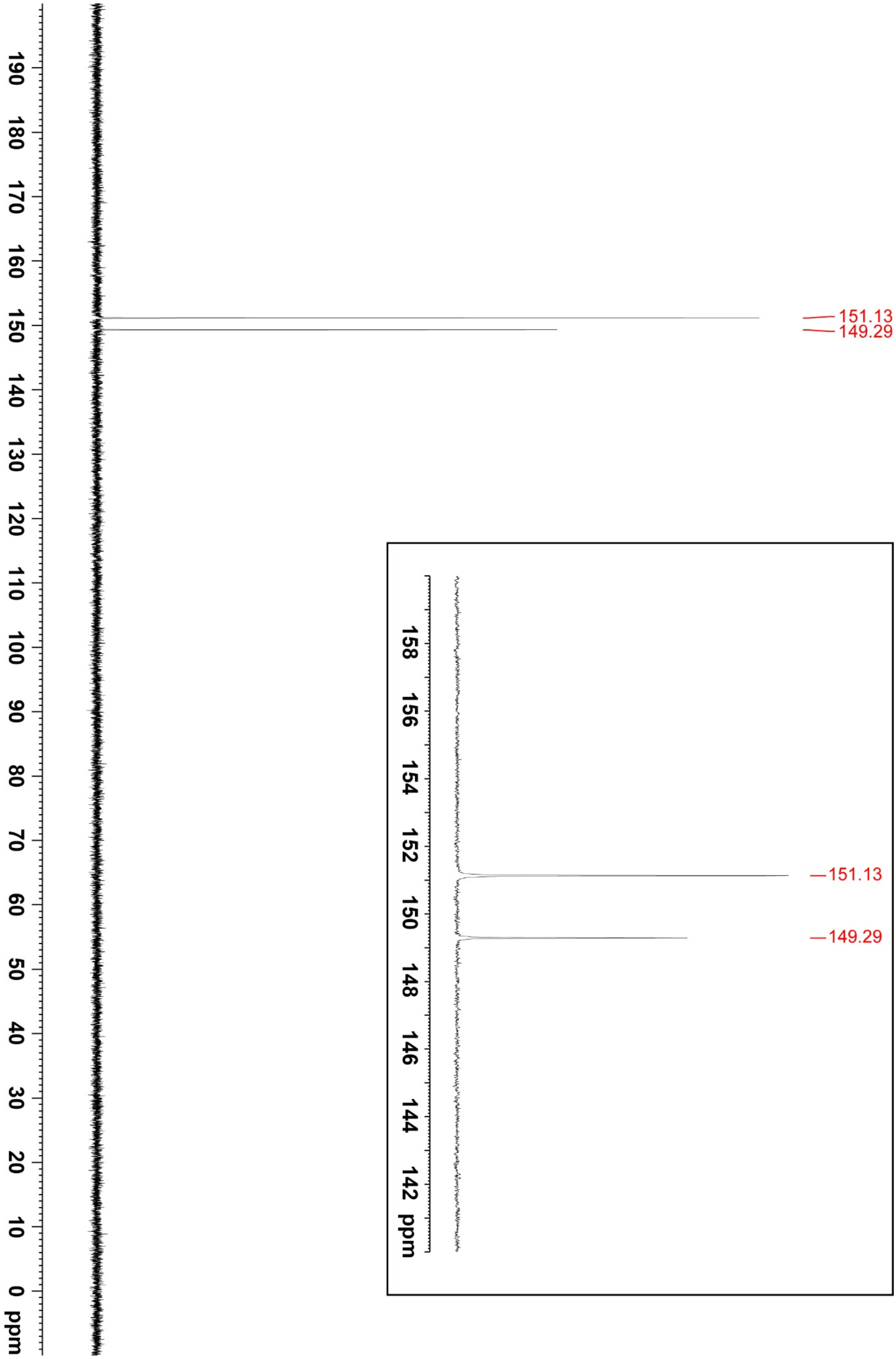

1.10. 1-(2-Iodoethyl)-4-nitrobenzene

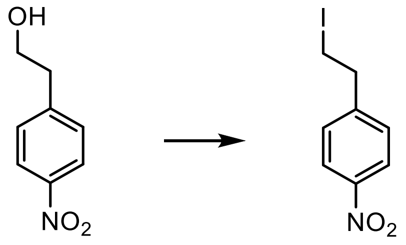

10

Triphenylphosphane (2.09 g, 7.96 mmol, 1.33 eq) and imidazole (0.542 g, 7.96 mmol, 1.33 eq) were dissolved in anhydrous toluene. A solution of iodine (2.02 g, 7.96 mmol, 1.33) eq in toluene (20 mL) was added dropwise and the solution was stirred for 30 minutes. 2-(4-Nitrophenyl)-ethanol (1.00 g, 5.98 mmol, 1 eq) in toluene (20 mL) was added and the reaction mixture was stirred for 3 hours at room temperature. Sat. aqu. sodium bicarbonate solution (20 mL) was added and stirring was continued for 10 minutes. The organic phase was separated and stirred with LiI (1.06 g, 7.96 mmol, 1.33 eq) for another 10 minutes. The solution was extracted once with sat. aqu. sodium thiosulfate and twice with water. The triphenylphosphinoxide can then be directly precipitated from the solution with petroleum ether and filtrated. The solvent is evaporated to dryness and the crude product is purifies via column chromatography (SiO_2, 10-15% ethyl acetate in cyclohexane). Yield: 1.40 g of compound **10** as a brown oil (85%). TLC: 0.62 (3/7 ethyl acetate/cyclohexane). ^{1}H NMR (400 MHz, $CDCl_3$): δ 3.27 - 3.31 (m, 2H, H_2C), 3.37-3.41 (m, 2H, H_2C), 7.35-7.38 (m, 2H, HC(ar)), 8.16-8.19 (m, 2H, HC(ar)), ppm. ^{13}C NMR (100 MHz, $CDCl_3$): δ 3.79 (s, 1C, CH_2), 39.58 (s, 1C, CH_2), 124.03 (s, 1C, C(ar)), 129.41 (s, 1C, C(ar)), 147.10 (s, 1C, C(ar)), 147.79 (s, 1C, C(ar)), ppm;

^{1}H-NMR (400 Mhz, $CDCl_3$, 25 °C) of compound **10**

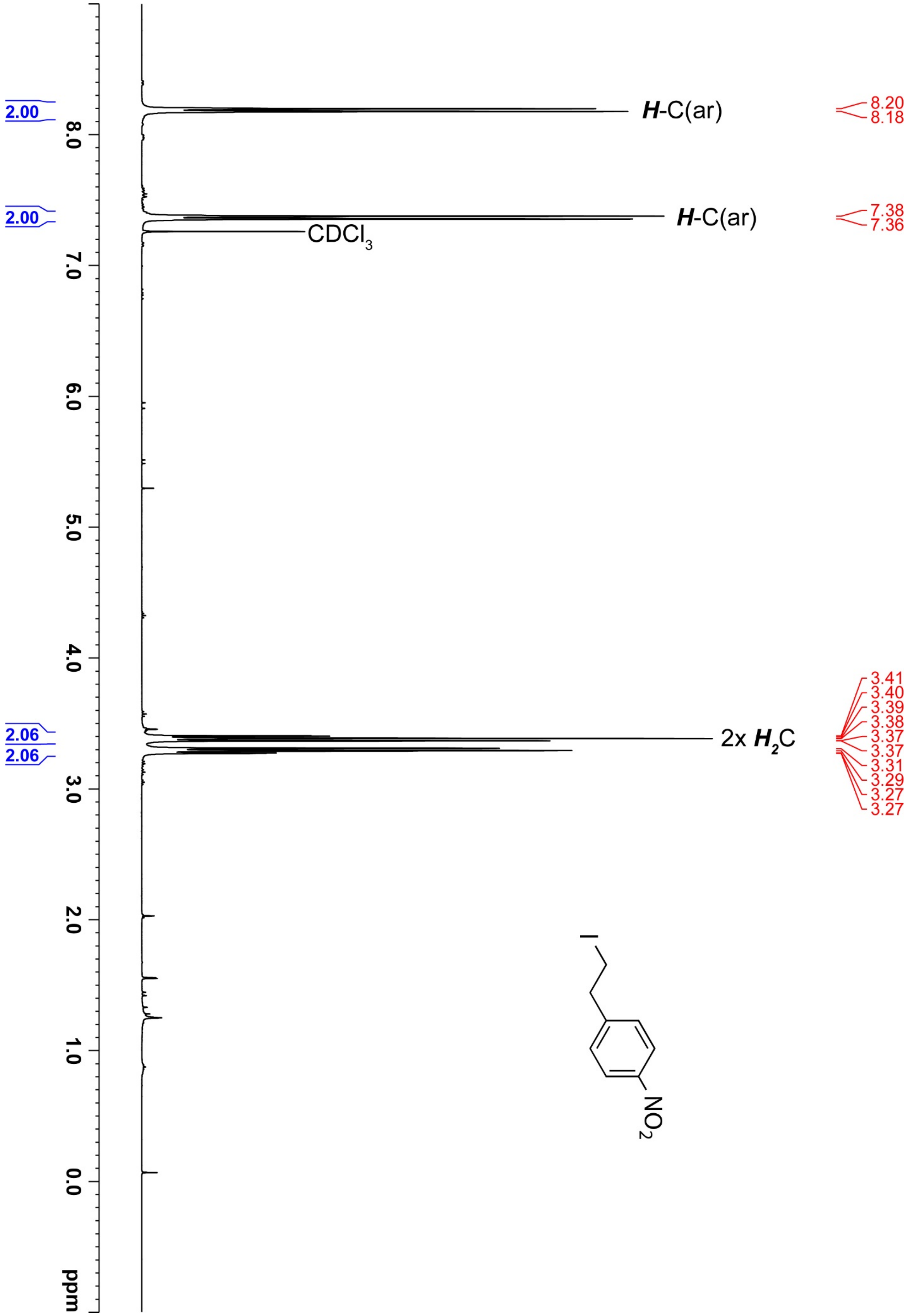

13C-NMR (100 Mhz, CDCl3, 25 °C) of compound **10**

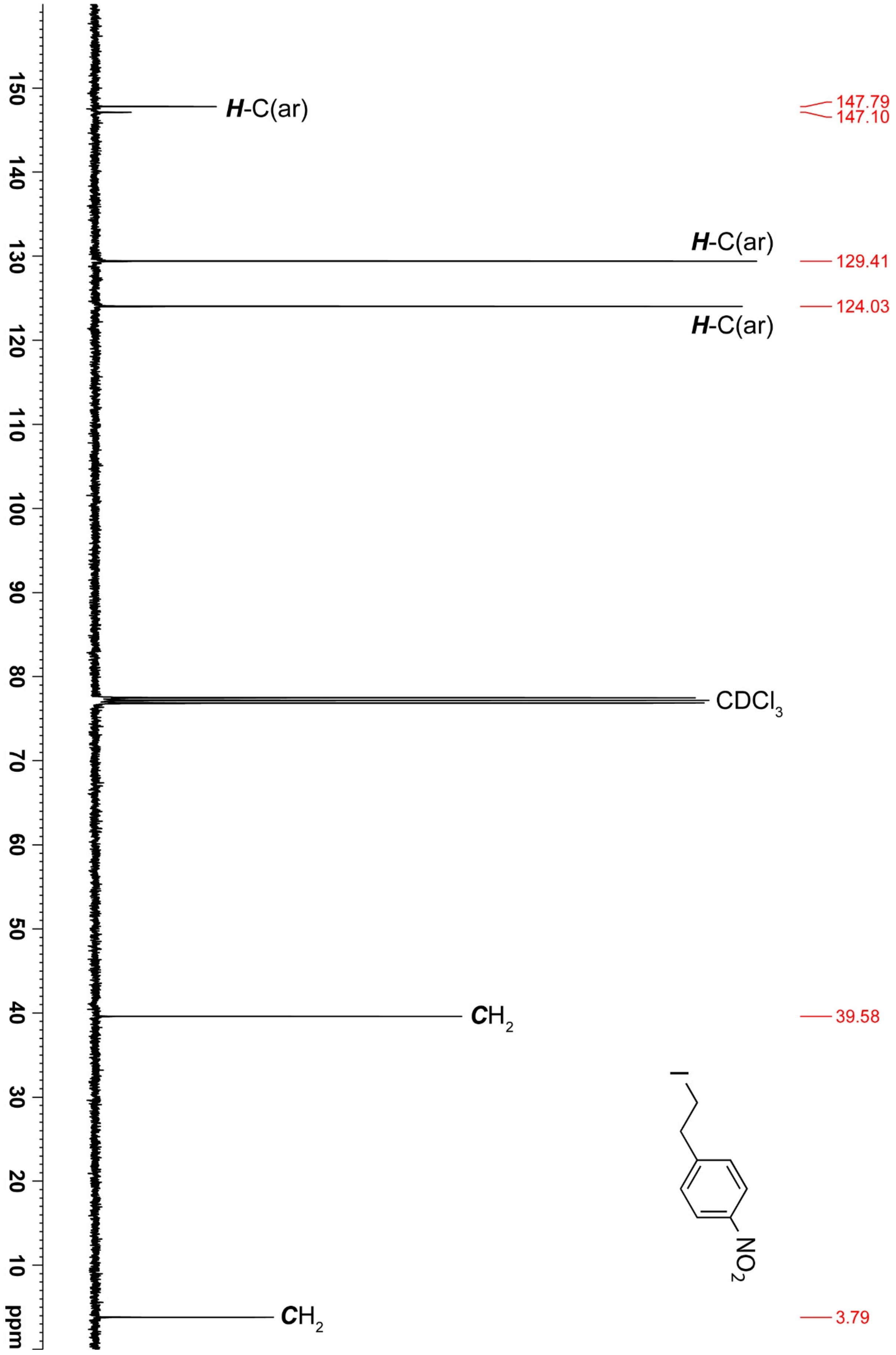

Supporting Table 1. Overview of synthesized RNAs and mass spectrometric analysis.

	Sequence (5' to 3')	nt	m.w. [Da] (calculated)	m.w. [Da] (measured)
**Ia**	GGCAGAGGC	9	2932.87	2932.97
**Ib**	GGCAAAGGC	9	2916.87	2916.70
**Ic**	GGCAXAGGC	9	2933.85	2934.00
**Id**	GCCUCUGCC	9	2766.70	2766.78
**Ie**	GCCUUUGCC	9	2767.69	2767.95
**If**	GCCUGUGCC	9	2806.73	2806.89
**Ig**	GCCUAUGCC	9	2790.73	2791.02
**IIa**	GGACCGGUCC	10	3174.98	3174.79
**IIb**	GGAUCGAUCC	10	3159.97	3159.98
**IIc**	GGACCGXUCC	10	3175.96	3175.89
**IId**	GGAUCGXUCC	10	3176.95	3176.51
**IIe**	GGAUCGGUCC	10	3175.97	3175.99
**IIIa**	GAAGGGCAACCUUCG	15	4813.99	4814.08
**IIIb**	GAAXGGCAACCUUCG	15	4814.97	4814.96
**IIIc**	GAXGGGCAACCUUCG	15	4830.97	4830.72
**IIId**	GAGGGGCAACCUUCG	15	4829.99	4830.01
**IVa**	AAUGUAAAACGACGGCCAGGCU-<br>UAAGCCCUAAXCGUUGAUAGUUAG	46	14828.01	14827.81
**IVb**	p-AUUCCUCAUCAUCCXUACA-<br>GACAGAACUAACGAUUCG	37	11809.09	11808.61
**IVc**	AAUGUAAAACGACGGCCAGGCU-<br>UAAGCCCUAAXCGUUGAUAGUUAG-<br>AUUCCUCAUCAUCCXUACA-<br>GACAGAACUAACGAUUCG	83	26618.07	26617.89
**V**	AUUCCUCXUCAUCCAUACAGACA-<br>GAACUAACGAUUCG	37	11728.10	11728.31

Supporting Table 2. Complete set of thermodynamic data of xanthosine modified RNA.

No.	RNA sequences 5' to 3'	T_m [°C] [b]	ΔT_m [°C] vs GC / AU	$\Delta G°298$ [kcal mol^{-1}] [c]	$\Delta H°298$ [kcal mol^{-1}] [c]	$\Delta S°$ [cal mol^{-1} K^{-1}] [c]
**Ia/Id**	GGCAGAGGC / GCCUCUGCC	**67.7 ± 0.2**	-	-17.2±0.3	-84.0±2.0	-224±6
**Ib/Ie**	GGCAAAGGC / GCCUUUGCC	**59.5 ± 0.1**	-8.2	-15.4±0.1	-84.0±0.7	-230±2
**Ic/If**	GGCAXAGGC / GCCUGUGCC	**45.7 ± 0.4**	-22.0 / -13.8	-11.4±0.1	-71.9±1.0	-203±4
**Ic/Ig**	GGCAXAGGC / GCCUAUGCC	**45.1 ± 0.3**	-22.6 / -14.4	-11.3±0.3	-72.4±3.6	-205±11
**Ic/Id**	GGCAXAGGC / GCCUCUGCC	**46.8 ± 0.2**	-20.9 / -12.7	-12.2±0.2	-80.9±2.7	-230±8
**Ic/Ie**	GGCAXAGGC / GCCUUUGCC	**55.1 ± 0.2**	-12.6 / -4.4	-14.9±0.2	-89.4±1.7	-250±5
**Ia/Ie**	GGCAGAGGC / GCCUUUGCC	**54.3 ± 0.1**	-13.4 / -5.2	-14.0±0.3	-81.3±2.8	-226±9
**IIa** pH 7	GGACCGGUCC (Palindrome)	**74.5 ± 0.2**	-	-18.7±0.2	-83.9±1.9	-219±6
**IIa** pH 6		**73.5 ± 0.1**	-1.0	-19.6±0.5	-91.9±3.3	-243±9
**IIa** pH 5		**71.3 ± 0.1**	-3.2	-20.4±0.3	-102.1±1.9	-274±5
**IIb** pH 7	GGAUCGAUCC (Palindrome)	**59.7 ± 0.3**	-14.8	-15.8±0.1	-87.0±0.3	-239±1
**IIb** pH 6		**60.9 ± 0.1**	-13.6	-16.1±0.1	-87.5±0.2	-240±1
**IIb** pH 5		**58.9 ± 0.2**	-15.6	-15.6±0.3	-86.5±2.5	-238±8
**IIc** pH7	GGACCGXUCC (Palindrome)	**42.0 ± 0.2**	-32.5 / -17.7	–11.6±0.1	-89.9±1.7	-263±5
**IIc** pH 6		**48.9 ± 0.3**	-25.6 / -10.9	-13.5±0.3	-91.6±4.4	-262±14
**IIc** pH 5		**51.1 ± 0.4**	-23.4 / -8.6	-13.5±0.2	-83.7±2.9	-236±9
**IId** pH 7	GGAUCGXUCC (Palindrome)	**55.5 ± 0.2**	-19.0 / -4.2	–15.1±0.1	–90.7±1.2	–254±4
**IId** pH 6		**54.5 ± 0.2**	-20.0 / -5.2	-15.2±0.1	-94.1±0.1	-264.6±1
**IId** pH 5		**51.2 ± 0.2**	-23.3 / -8.5	-14.9±0.2	-101.6±1.7	-291±5
**IIe** pH 7	GGAUCGGUCC (Palindrome)	**56.0 ± 0.2**	-18.5 / -3.7	–15.6±0.1	–94.0±0.7	–263±2
**IIe** pH 6		**55.6 ± 0.2**	-18.9 / -4.1	-15.6±0.5	-95.2±4.8	-270±11
**IIe** pH 5		**53.5 ± 0.1**	-21.0 / -6.2	-15.0±0.3	-91.7±1.5	-267.6±9
**IIIa**	GAAGGGCAACCUUCG (Hairpin)	**71.1 ± 0.1**	-	–7.6±0.1	–57.7±0.3	–168±1
**IIIb**	GAAXGGCAACCUUCG (Hairpin)	**39.4 ± 0.1**	-31.7	–1.8±0.1	–37.2±1.3	–120±4
**IIIc**	GAXGGGCAACCUUCG (Hairpin)	**65.8 ± 0.4**	-5.3	–6.1±0.1	–51.6±1.7	–153±5
**IIId**	GAGGGGCAACCUUCG (Hairpin)	**64.2 ± 0.1**	-6.9	-4.9±0.1	-43.7±0.7	-130±2

[a] Buffer: 10 mM Na_2HPO_4, 150 mM NaCl, pH 7.0. ΔH and ΔS values were obtained by van't Hoff analysis or based on RNA concentration dependent measurements according to references 18 and 19. [b] The estimated errors of UV-spectroscopically determined T_m values are ±0.3 °C. [c] Errors for ΔH and ΔS were determined from at least three independent measurements; in general, errors arising from noninfinite cooperativity of two-state transitions and from the assumption of a temperature-independent enthalpy, are typically 10−15%. Additional error is introduced when free energies are extrapolated far from melting transitions; errors for ΔG are typically 3−5%.

Supporting Table 3. X-ray data collection and crystallographic refinement statistics.

	Xan-2648-SRL	Xan-12-mer	Xan-14-mer
PDB ID	7QSH	7QUA	7QTN
Space group	$P4_3$	C2	$P6_322$
a (Å)	29.50	41.07	44.88
b (Å)	29.50	35.15	44.88
c (Å)	76.51	31.93	149.70
β (°)	90	129.05	90
Beamline	SLS PX III - X06DA	SLS PX III - X06DA	SLS PX III - X06DA
Resolution range (Å)	50 – 0.86	50 – 1.00	50 – 1.20
Number of frames	9000	3600	5400
Oscillation angle	0.2°	0.2°	0.2°
Wavelength	0.9	0.9	1.0
Average redundancy	55.8	11.2	65.3
Completeness [1]	98.9% (86.9%)	98.8% (81.8%)	100.0% (100.0%)
$CC_{1/2}$ [1]	100% (49.0%)	100% (24.6%)	100% (20.2%)
Average I/σ [1]	56.0 (1.4)	18.9 (0.9)	32.3 (0.7)
ISa0	22	20	22
R/R_{free}	14.6 / 16.8	13.8 / 15.8	18.3 / 20.9
Coordinate error (Å)	0.10	0.18	0.23
Wilson B (Å²)	7.9	9.3	18.8
Number of molecules			
RNA strands	1	1	2
Water	147	108	122
Glycerol	1	0	0
Sulfate	1	0	0
Magnesium	0	2	0
Sodium	0	2	0

[1] Values for last resolution shell are shown in parenthesis

Ia

5‘ 3‘
G C
G C
C G
A U
G C
A U
G C
G C
C G
3‘ 5‘
Id

concentration / µM	T_m(duplex) / °C
2.60	63.1
5.16	65.2
12.3	67.5
37.0	70.7
92.4	73.2

ΔG^0 kcal mol^{-1}	ΔH^0 kcal mol^{-1}	ΔS^0 cal mol^{-1}
-17.0	-82.4	-220

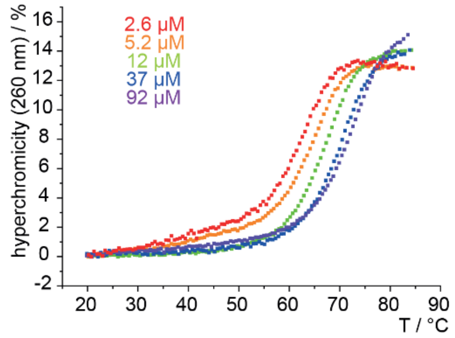

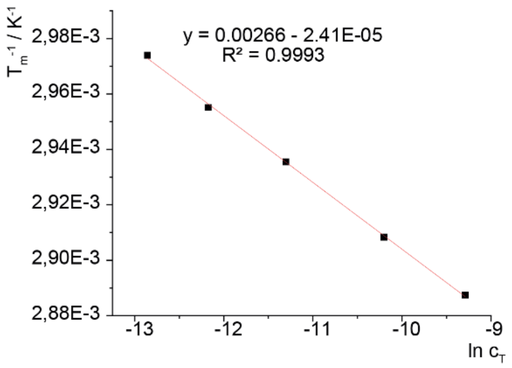

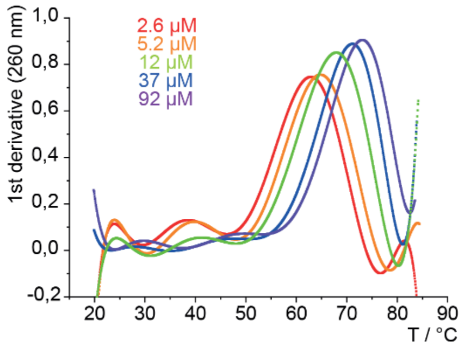

Ib

5‘ 3‘
G C
G C
C G
A U
A U
A U
G C
G C
C G
3‘ 5‘
Ie

concentration / µM	T_m(duplex) / °C
2.62	55.1
5.54	57.5
12.5	59.6
39.1	62.4
97.4	64.8

ΔG^0 kcal mol^{-1}	ΔH^0 kcal mol^{-1}	ΔS^0 cal mol^{-1}
-15.3	-83.2	-228

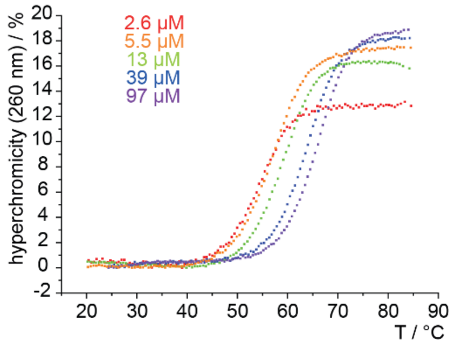

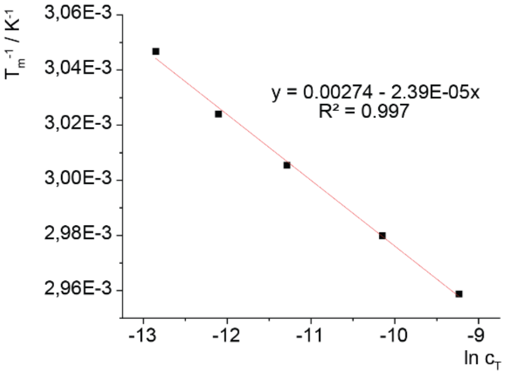

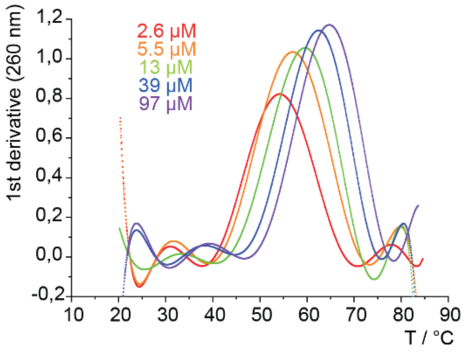

Supporting Figure 1. UV-melting profile analysis of RNA duplexes **Ia/Ib** and **Ib/Ie**.

Ic

5'	3'
G	C
G	C
C	G
A	U
X	G
A	U
G	C
G	C
C	G
3'	5'

If

concentration / µM	T_m(duplex) / °C
2.25	40.7
7.10	43.8
16.2	46.0
50.1	49.7
125	51.7

ΔG^0 kcal mol^{-1}	ΔH^0 kcal mol^{-1}	ΔS^0 cal mol^{-1}
-11.3	-72.1	-204

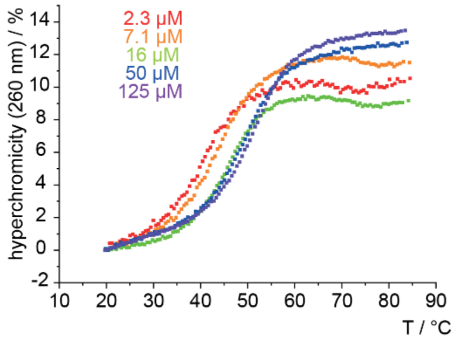

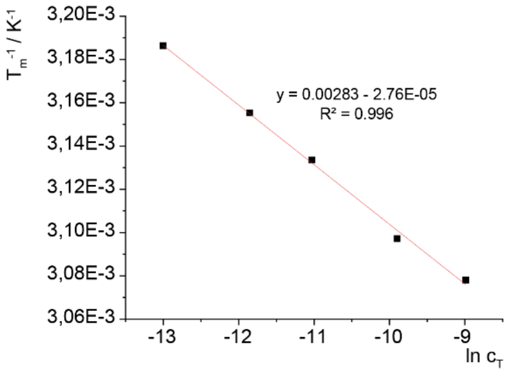

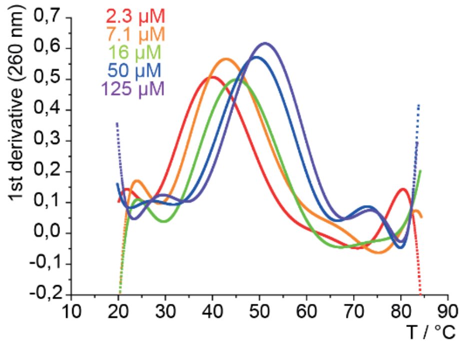

Ic

5'	3'
G	C
G	C
C	G
A	U
X	A
A	U
G	C
G	C
C	G
3'	5'

Ig

concentration / µM	T_m(duplex) / °C
1.93	39.8
4.40	42.5
9.84	44.5
30.2	48.0
75.8	50.1

ΔG^0 kcal mol^{-1}	ΔH^0 kcal mol^{-1}	ΔS^0 cal mol^{-1}
-11.2	-72.3	-201

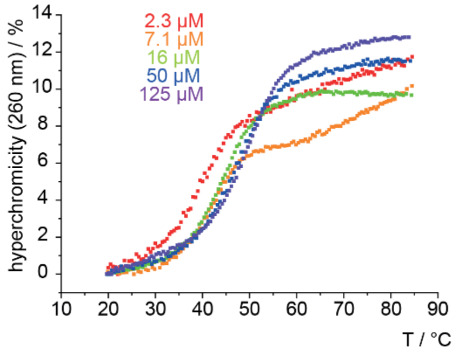

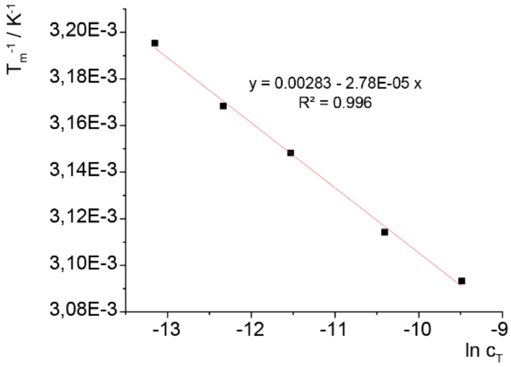

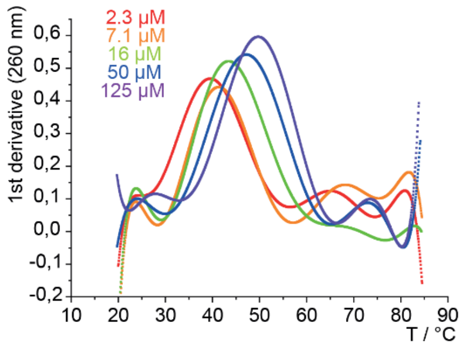

Supporting Figure 2. UV-melting profile analysis of RNA duplexes **Ic/If** and **Ic/Ig**.

Ic

5‘ 3‘
G C
G C
C G
A U
X C
A U
G C
G C
C G
3‘ 5‘

Id

concentration / µM	T_m(duplex) / °C
2.41	42.5
5.48	44.3
12.6	46.7
37.3	49.9
94.4	51.9

ΔG^0 kcal mol^{-1}	ΔH^0 kcal mol^{-1}	ΔS^0 cal mol^{-1}
-17.0	-82.4	-220

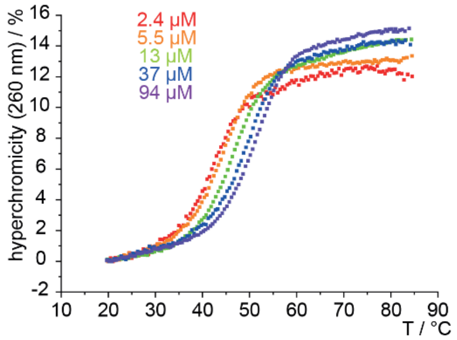

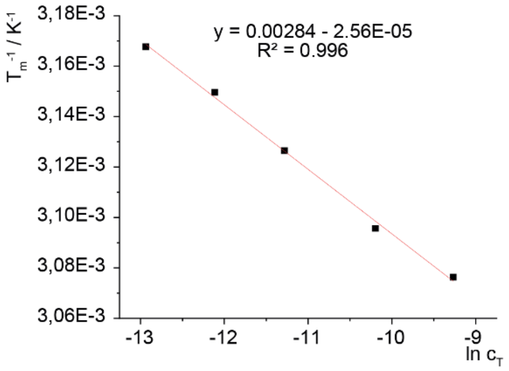

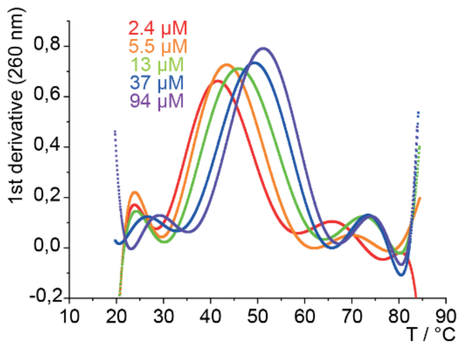

Ic

5‘ 3‘
G C
G C
C G
A U
X U
A U
G C
G C
C G
3‘ 5‘

Ie

concentration / µM	T_m(duplex) / °C
2.69	51.1
5.84	53.5
12.9	55.2
39.6	57.8
99.4	60.2

ΔG^0 kcal mol^{-1}	ΔH^0 kcal mol^{-1}	ΔS^0 cal mol^{-1}
-14.7	-87.2	-243

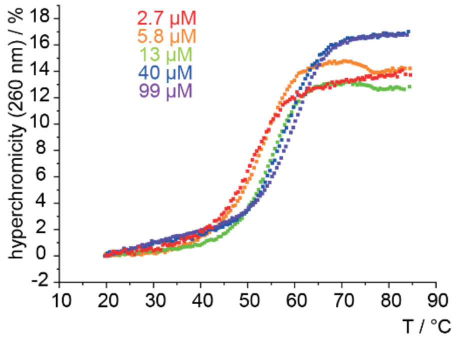

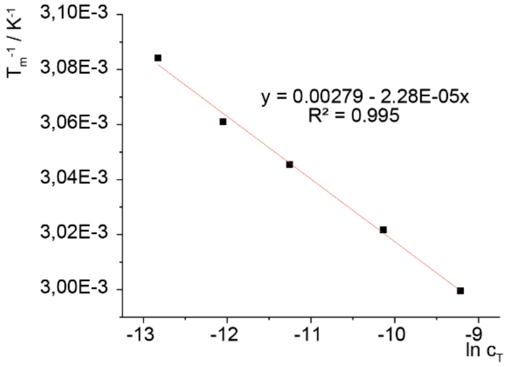

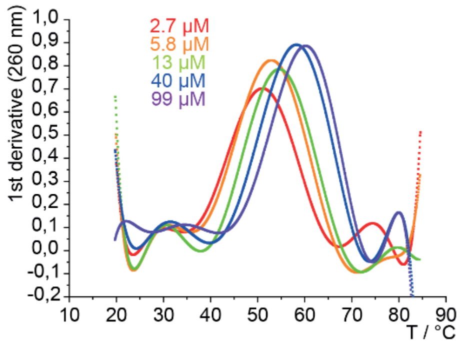

Supporting Figure 3. UV-melting profile analysis of RNA duplexes **Ic/Id** and **Ic/Ie**.

Ia

5‘ 3‘
G C
G C
C G
A U
G U
A U
G C
G C
C G
3‘ 5‘
Ie

concentration / µM	T_m(duplex) / °C
2.01	49.7
5.10	52.1
12.0	54.1
37.3	57.2
93.1	59.6

ΔG^0 kcal mol^{-1}	ΔH^0 kcal mol^{-1}	ΔS^0 cal mol^{-1}
-14.1	-82.8	-230

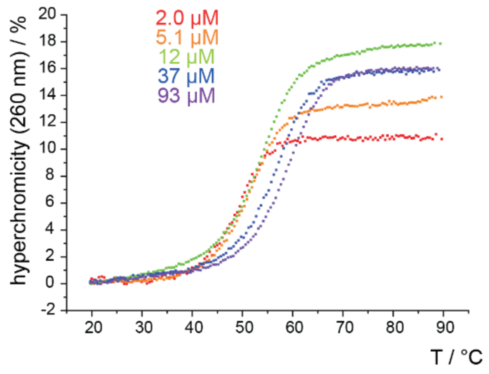

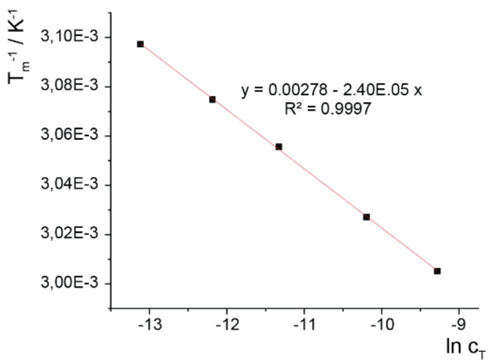

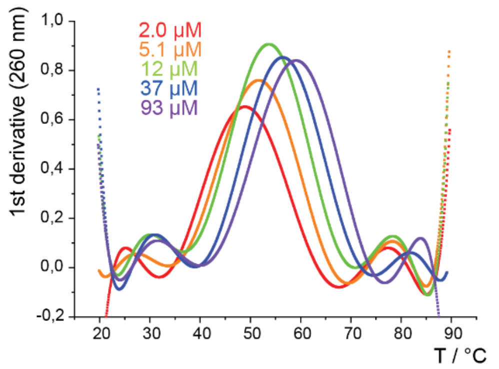

Supporting Figure 4. UV-melting profile analysis of RNA duplex **Ia/Ie**.

IIa **pH = 7.00**

5‘ 3‘
G C
G C
A U
C G
C G
G C
G C
U A
C G
C G
3‘ 5‘
IIa

concentration / µM	T_m(duplex) / °C
2.55	70.2
6.01	72.6
13.9	74.4
40.1	77.5
77.9	79.9

ΔG^0 kcal mol^{-1}	ΔH^0 kcal mol^{-1}	ΔS^0 cal mol^{-1}
-19.0	-86.6	-227

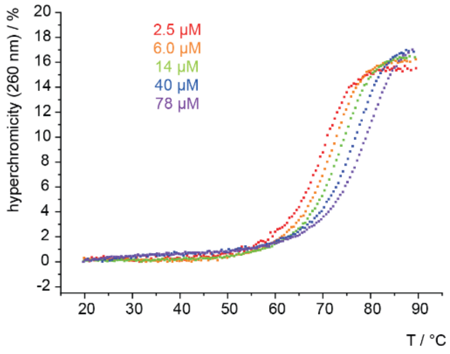

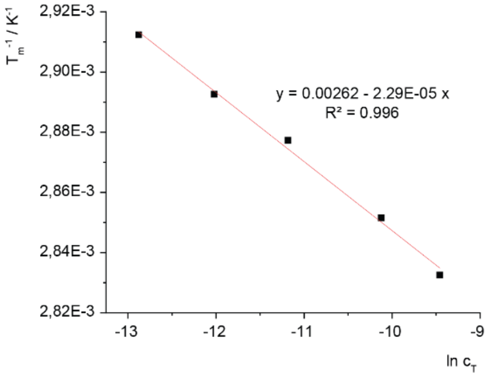

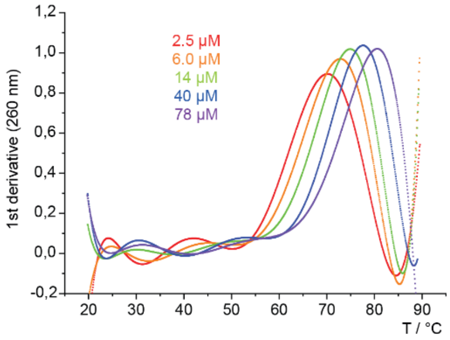

IIa **pH = 5.99**

5‘ 3‘
G C
G C
A U
C G
C G
G C
G C
U A
C G
C G
3‘ 5‘
IIa

concentration / µM	T_m(duplex) / °C
1.24	67.2
3.52	70.1
8.01	72.6
24.8	75.6
60.1	77.4

ΔG^0 kcal mol^{-1}	ΔH^0 kcal mol^{-1}	ΔS^0 cal mol^{-1}
-19.2	-89.2	-235

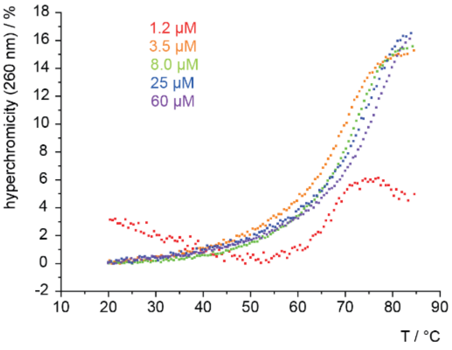

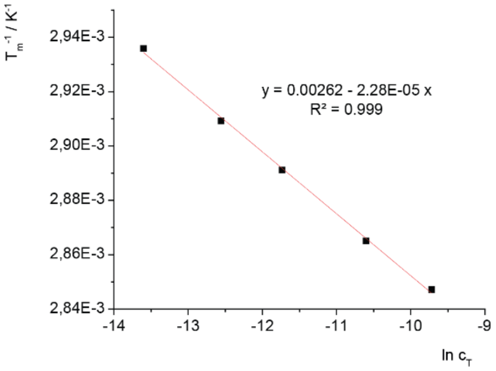

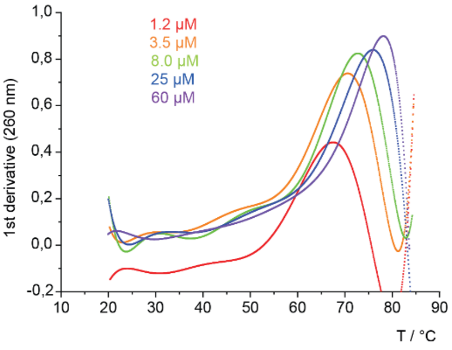

Supporting Figure 5. UV-melting profile analysis of RNA duplexes **IIa** at different pH 7 and pH 6.

IIa **pH = 5.00**

5‘ 3‘
G C
G C
A U
C G
C G
G C
G C
U A
C G
C G
3‘ 5‘
IIa

concentration / µM	T_m(duplex) / °C
1.79	66.7
5.18	69.4
11.8	71.4
36.4	73.7
89.7	70.0

ΔG^0 kcal mol^{-1}	ΔH^0 kcal mol^{-1}	ΔS^0 cal mol^{-1}
-20.2	-100.5	-269

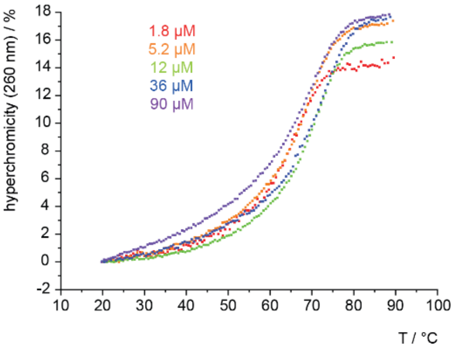

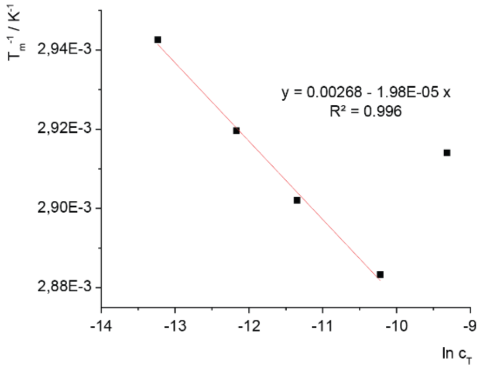

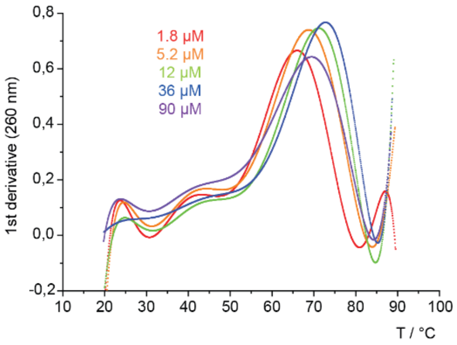

Ib **pH = 7.00**

5‘ 3‘
G C
G C
A U
U A
C G
G C
A U
U A
C G
C G
3‘ 5‘
Ib

concentration / µM	T_m(duplex) / °C
2.28	55.2
5.41	57.8
12.5	59.6
37.1	62.1
94.4	65.0

ΔG^0 kcal mol^{-1}	ΔH^0 kcal mol^{-1}	ΔS^0 cal mol^{-1}
-15.7	-86.7	-238

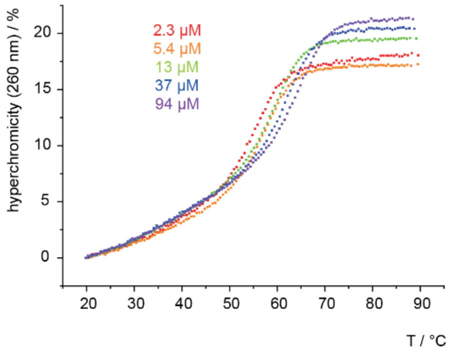

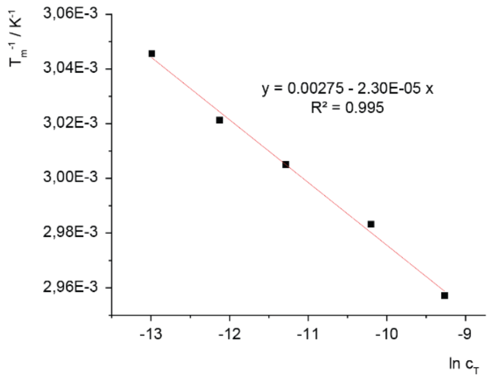

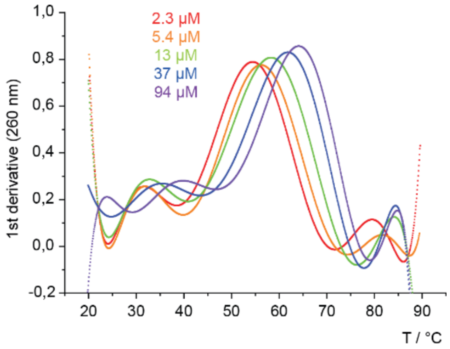

Supporting Figure 6. UV-melting profile analysis of RNA duplexes **IIa** at pH 5 and **Ib** and pH 7.

Ib **pH = 5.99**

5‘ 3‘
G C
G C
A U
U A
C G
G C
A U
U A
C G
C G
3‘ 5‘
Ib

concentration / µM	T_m(duplex) / °C
1.65	55.5
3.83	58.1
8.75	60.0
29.0	63.0
69.4	65.1

ΔG^0 kcal mol^{-1}	ΔH^0 kcal mol^{-1}	ΔS^0 cal mol^{-1}
-16.1	-87.5	-240

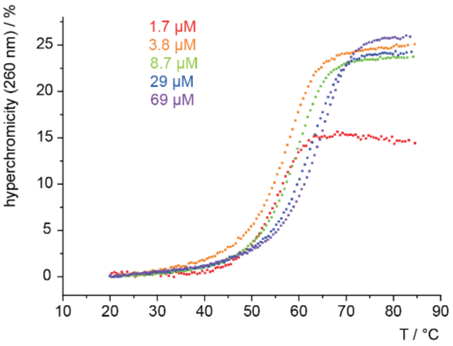

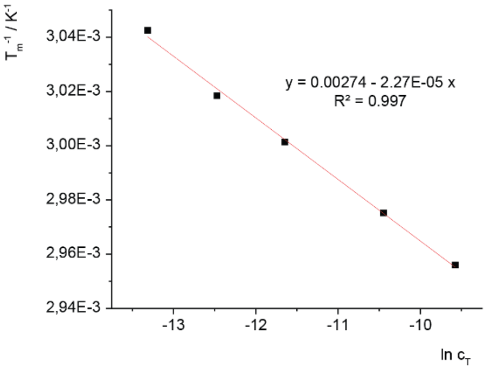

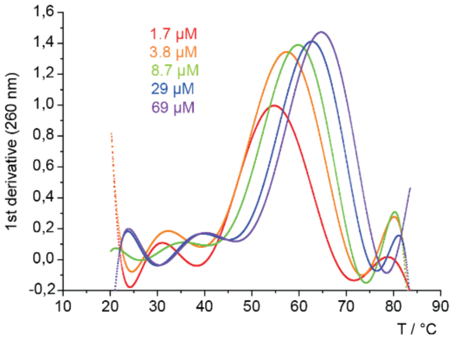

Ib **pH = 5.00**

5‘ 3‘
G C
G C
A U
U A
C G
G C
A U
U A
C G
C G
3‘ 5‘
Ib

concentration / µM	T_m(duplex) / °C
1.48	53.0
3.82	55.9
8.73	58.3
27.7	61.0
69.0	63.1

ΔG^0 kcal mol^{-1}	ΔH^0 kcal mol^{-1}	ΔS^0 cal mol^{-1}
-15.1	-82.9	-227

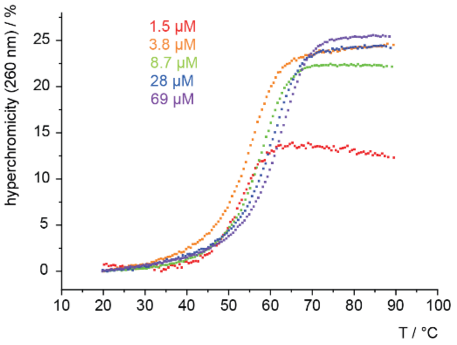

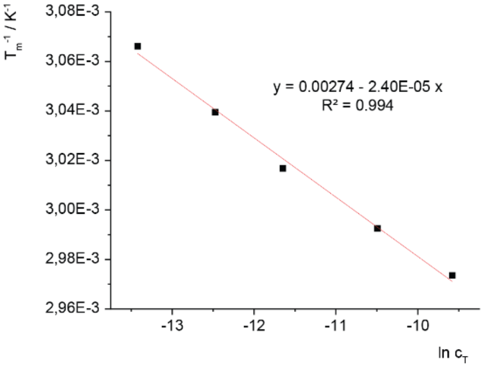

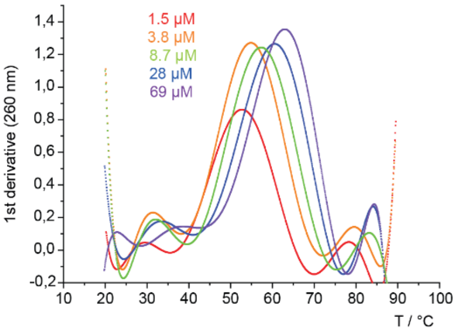

Supporting Figure 7. UV-melting profile analysis of RNA duplex **Ib** at pH 6 and pH 5.

IIc

5‘ 3‘
G C
G C
A U
C X
C G
G C
X C
U A
C G
C G
3‘ 5‘
IIc

pH = 7.00

concentration / µM	T_m(duplex) / °C
2.16	37.9
5.81	40.2
13.1	42.0
38.8	44.6
97.9	46.5

ΔG^0 kcal mol^{-1}	ΔH^0 kcal mol^{-1}	ΔS^0 cal mol^{-1}
-11.4	-87.6	-256

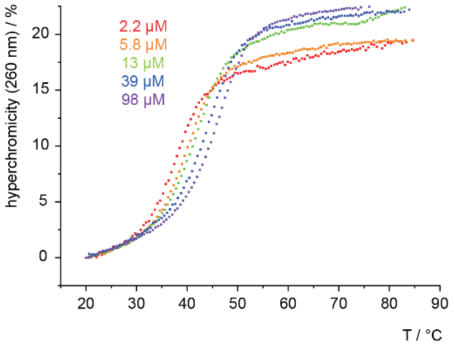

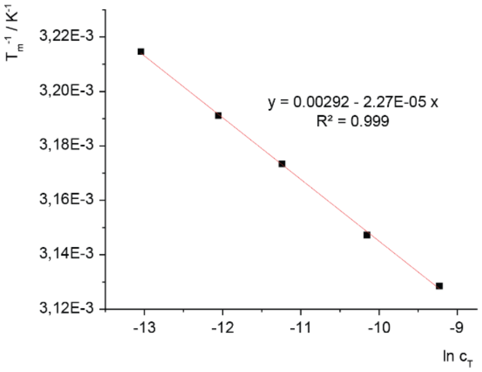

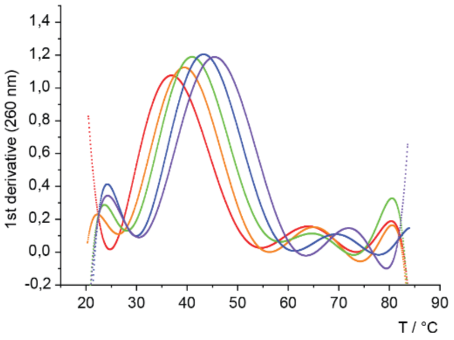

IIc

5‘ 3‘
G C
G C
A U
C X
C G
G C
X C
U A
C G
C G
3‘ 5‘
IIc

pH = 5.99

concentration / µM	T_m(duplex) / °C
2.10	44.9
5.56	47.0
12.8	48.8
37.5	51.0
97.6	53.3

ΔG^0 kcal mol^{-1}	ΔH^0 kcal mol^{-1}	ΔS^0 cal mol^{-1}
-13.7	-95.4	-274

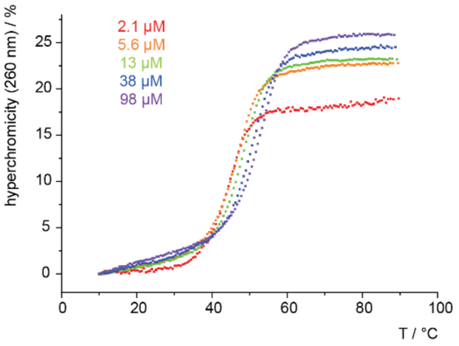

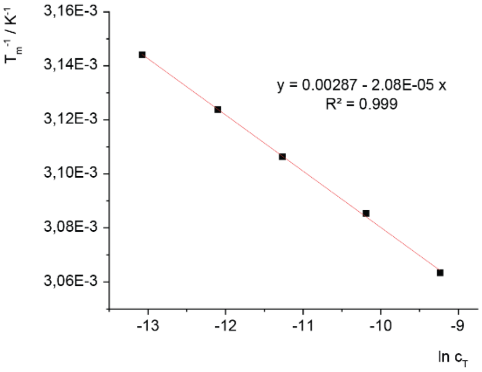

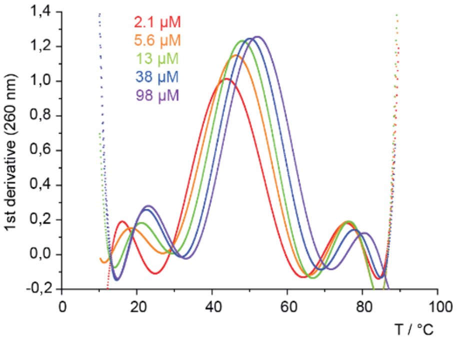

Supporting Figure 8. UV-melting profile analysis of RNA duplex **IIc** at pH 7 and pH 6.

IIc

5‘ 3‘
G C
G C
A U
C X
C G
G C
X C
U A
C G
C G
3‘ 5‘
IIc

pH = 5.00

concentration / µM	T_m(duplex) / °C
2.21	46.7
5.87	49.1
12.4	51.0
36.8	53.7
95.6	55.8

ΔG^0 kcal mol^{-1}	ΔH^0 kcal mol^{-1}	ΔS^0 cal mol^{-1}
-13.6	-86.2	-244

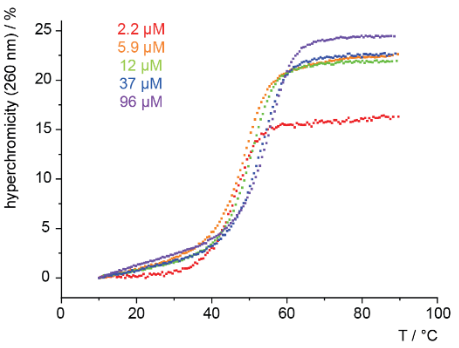

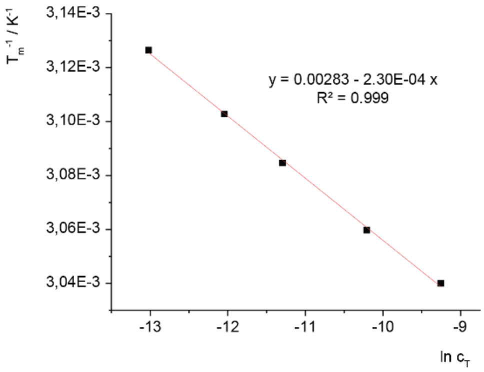

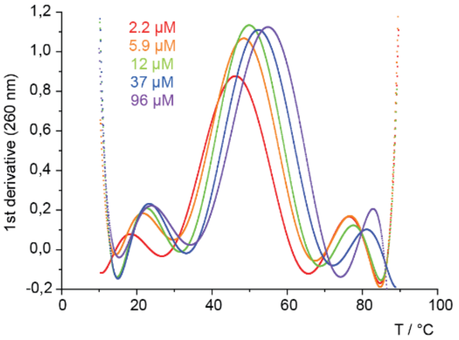

IId

5‘ 3‘
G C
G C
A U
U X
C G
G C
X U
U A
C G
C G
3‘ 5‘
IId

pH = 7.00

concentration / µM	T_m(duplex) / °C
2.26	51.6
5.34	53.7
12.4	55.4
37.1	58.2
94.3	60.2

ΔG^0 kcal mol^{-1}	ΔH^0 kcal mol^{-1}	ΔS^0 cal mol^{-1}
-15.3	-92.5	-258

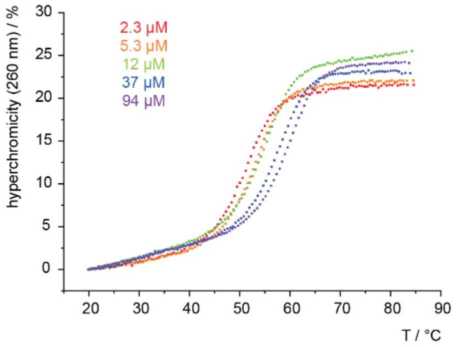

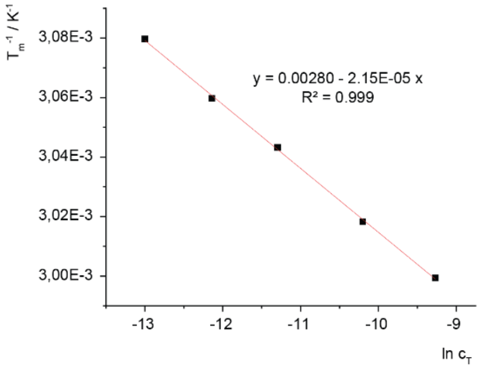

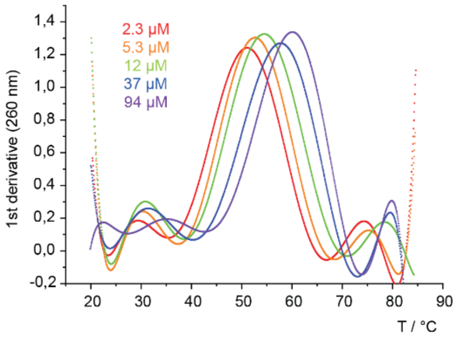

Supporting Figure 9. UV-melting profile analysis of RNA duplexes **IIc** at pH 5 and **IId** at pH 7.

IId **pH = 5.99**

5‘ 3‘
G C
G C
A U
U X
C G
G C
X U
U A
C G
C G
3‘ 5‘
IId

concentration / µM	T_m(duplex) / °C
2.21	49.6
5.57	51.7
13.2	53.4
38.9	55.7
98.5	68.0

ΔG^0 kcal mol^{-1}	ΔH^0 kcal mol^{-1}	ΔS^0 cal mol^{-1}
-15.1	-90.2	-252

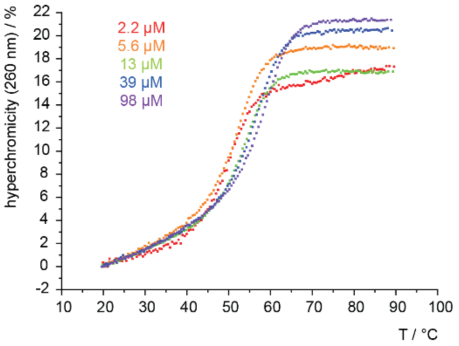

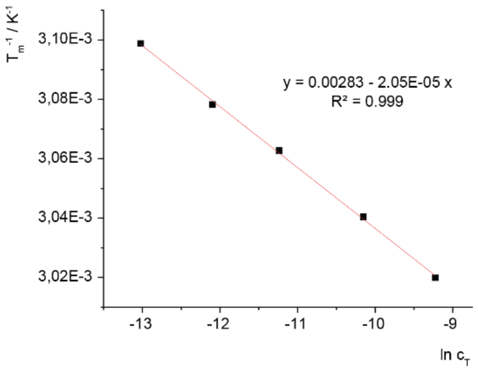

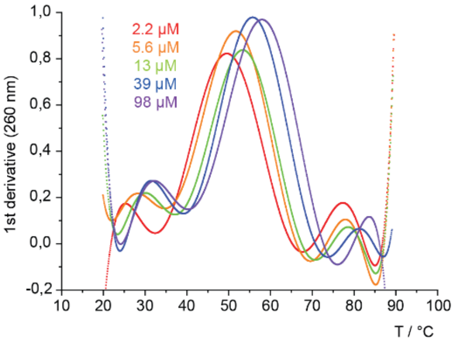

IId **pH = 5.00**

5‘ 3‘
G C
G C
A U
U X
C G
G C
X U
U A
C G
C G
3‘ 5‘
IId

concentration / µM	T_m(duplex) / °C
2.05	45.6
5.28	48.1
12.4	50.2
38.6	52.4
99.3	51.2

ΔG^0 kcal mol^{-1}	ΔH^0 kcal mol^{-1}	ΔS^0 cal mol^{-1}
-13.5	-88.8	-253

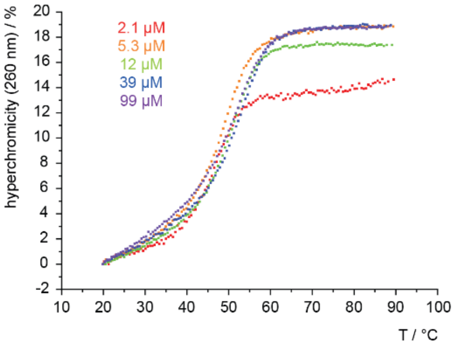

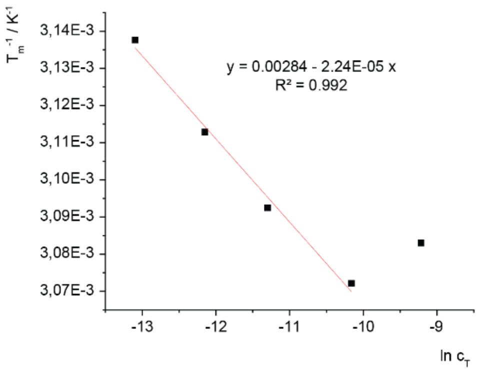

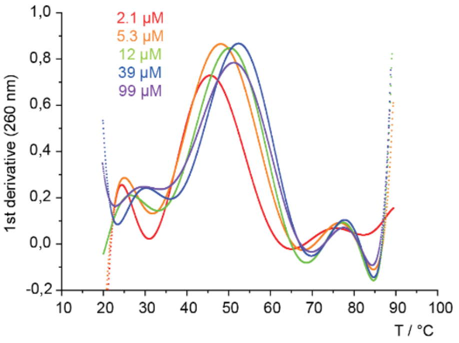

Supporting Figure 10. UV-melting profile analysis of RNA duplex **IId** at pH 6 and pH 5.

Ile **pH = 7.00**

5‘ 3‘
G C
G C
A U
U G
C G
G C
G U
U A
C G
C G
3‘ 5‘
Ile

concentration / µM	T_m(duplex) / °C
1.27	50.2
3.30	52.3
8.05	54.0
25.5	56.4
61.4	58.8

ΔG^0 kcal mol^{-1}	ΔH^0 kcal mol^{-1}	ΔS^0 cal mol^{-1}
-15.6	-94.3	-264

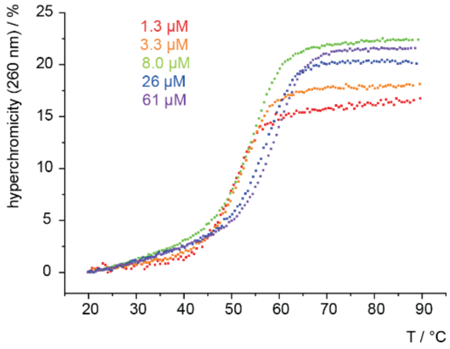

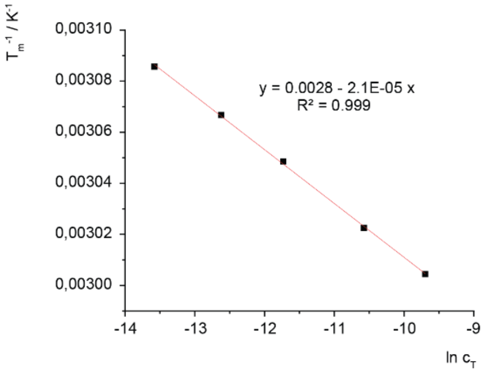

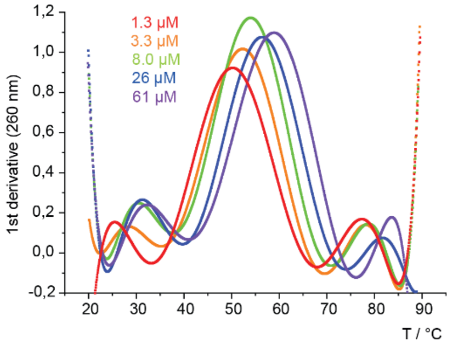

Ile **pH = 5.99**

5‘ 3‘
G C
G C
A U
U G
C G
G C
G U
U A
C G
C G
3‘ 5‘
Ile

concentration / µM	T_m(duplex) / °C
2.07	51.8
3.69	53.0
8.43	54.7
28.1	57.8
71.6	59.7

ΔG^0 kcal mol^{-1}	ΔH^0 kcal mol^{-1}	ΔS^0 cal mol^{-1}
-15.5	-94.6	-265

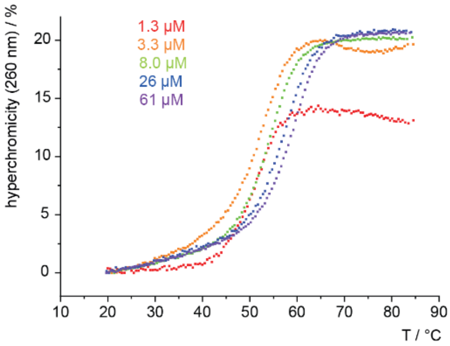

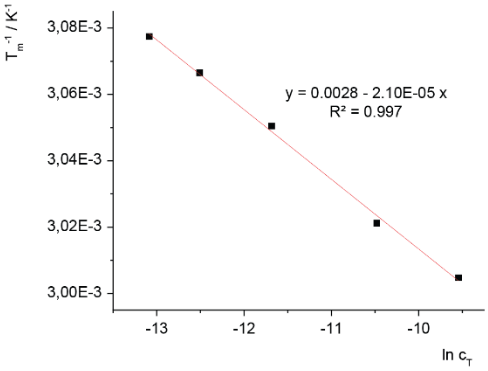

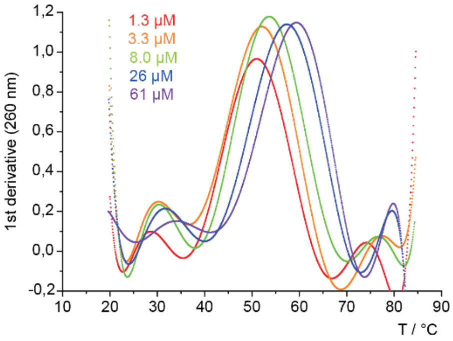

Supporting Figure 11. UV-melting profile analysis of RNA duplex **Ile** at pH 7 and pH 6.

IIe

5' 3'
G C
G C
A U
U G
C G
G C
G U
U A
C G
C G
3' 5'
IIe

pH = 5.00

concentration / µM	T_m(duplex) / °C
2.00	49.0
3.70	50.8
9.28	53.2
28.6	55.1
69.6	55.3

ΔG^0 kcal mol^{-1}	ΔH^0 kcal mol^{-1}	ΔS^0 cal mol^{-1}
-14.7	-92.2	-260

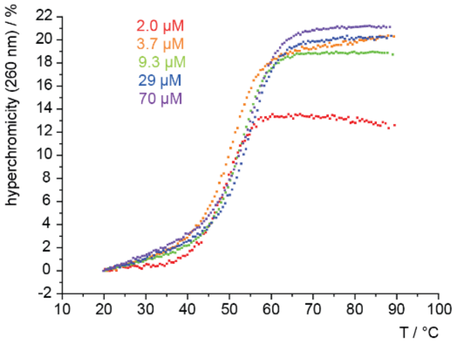

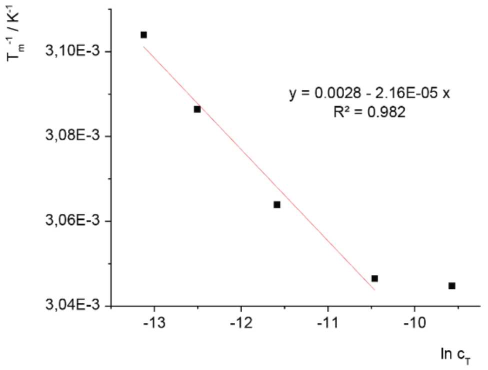

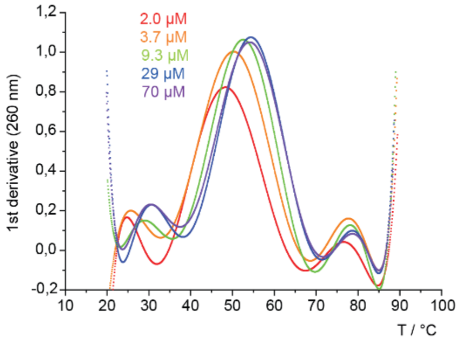

Supporting Figure 12. UV-melting profile analysis of RNA duplex **IIe** at pH 5.

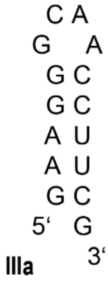

concentration / µM	T_m(hairpin) / °C
2.04	71.2
15.9	70.6

ΔG^0 kcal mol^{-1}	ΔH^0 kcal mol^{-1}	ΔS^0 cal mol^{-1}
-7.49	-57.2	-167

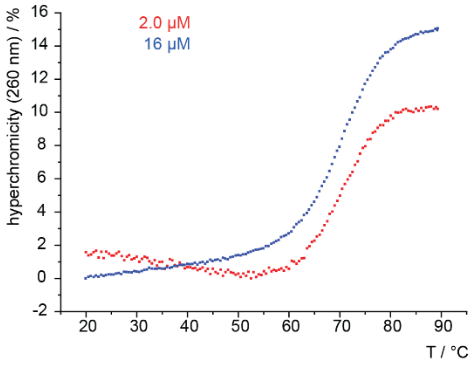

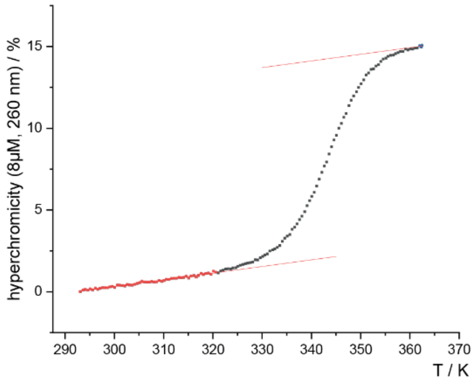

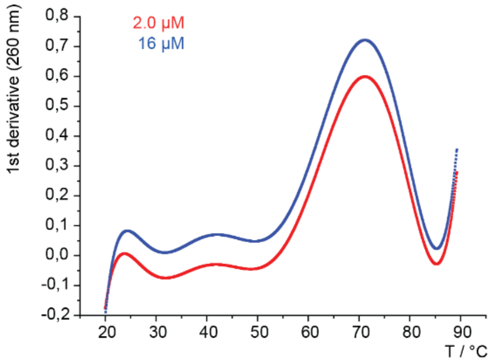

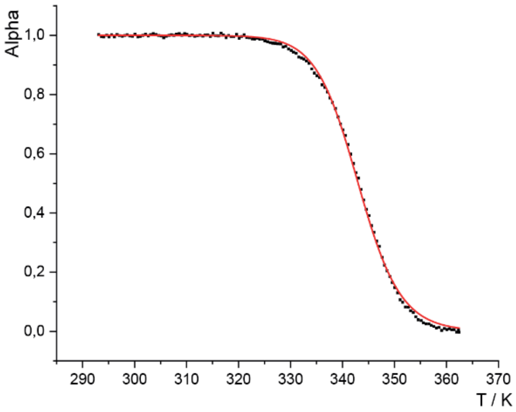

Supporting Figure 13. UV-melting profile analysis of RNA hairpin **IIIa** at pH 7.

C A
G A
G C
X C
A U
A U
G C
5‘ G
3‘
IIIb

concentration / µM	T_m(hairpin) / °C
2.49	39.7
8.62	39.6

ΔG^0 kcal mol^{-1}	ΔH^0 kcal mol^{-1}	ΔS^0 cal mol^{-1}
-1.80	-37.2	-119

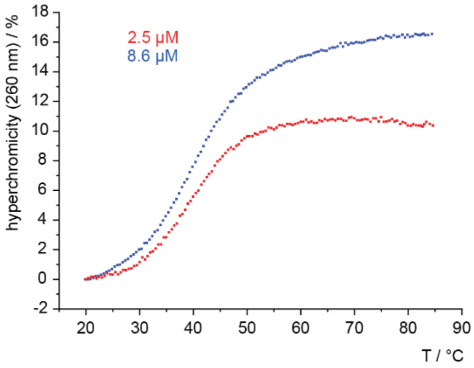

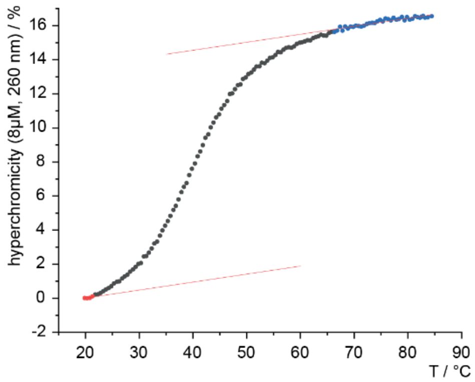

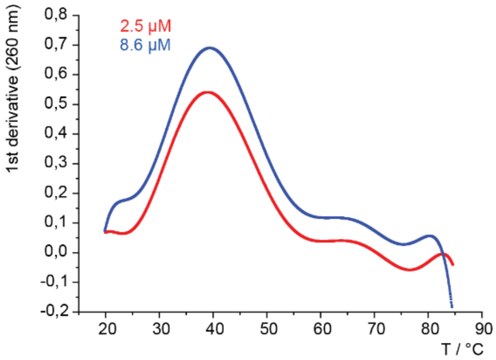

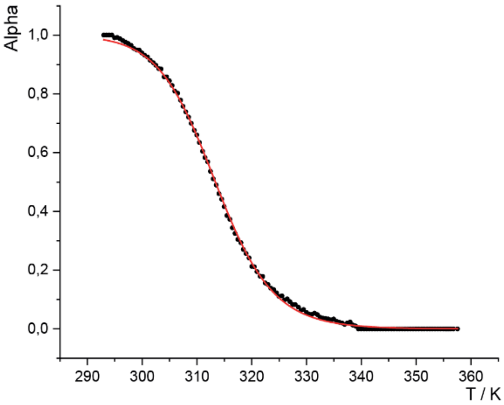

Supporting Figure 14. UV-melting profile analysis of RNA hairpin **IIIb** at pH 7.

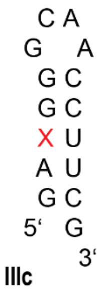

concentration / µM	T_m(hairpin) / °C
2.23	65.6
8.50	65.6

ΔG^0 kcal mol^{-1}	ΔH^0 kcal mol^{-1}	ΔS^0 cal mol^{-1}
-1.80	-37.2	-119

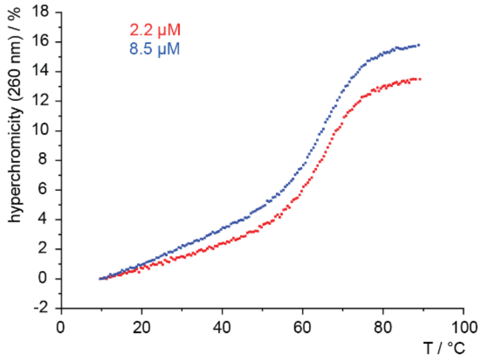

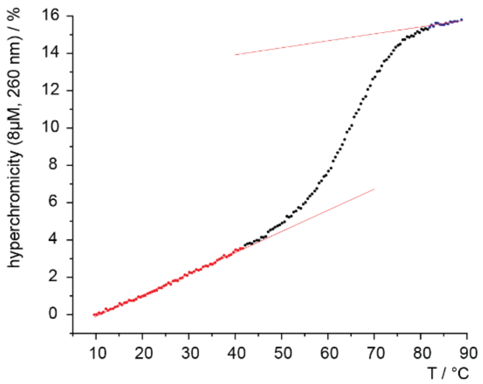

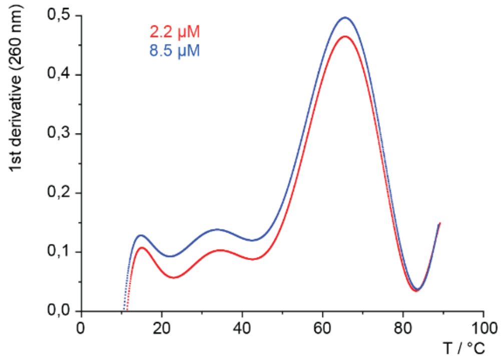

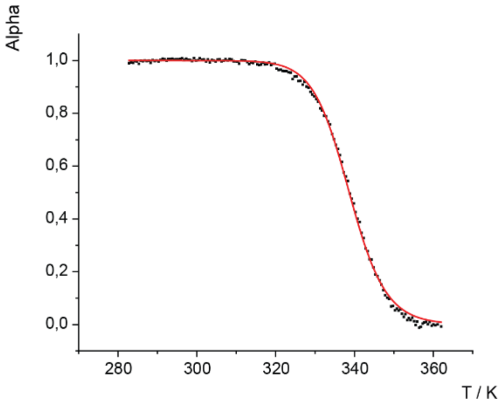

Supporting Figure 15. UV-melting profile analysis of RNA hairpin **IIIc** at pH 7.

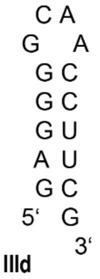

concentration / µM	T_m(hairpin) / °C
2.23	64.2
8.33	64.3

ΔG^0 kcal mol^{-1}	ΔH^0 kcal mol^{-1}	ΔS^0 cal mol^{-1}
-4.83	-43.3	-129

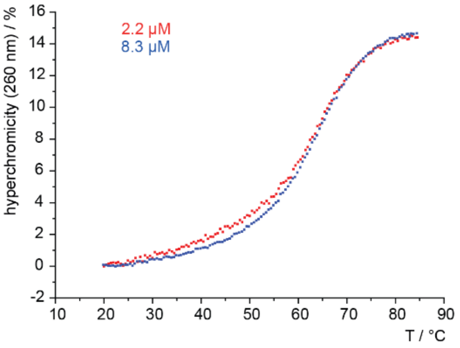

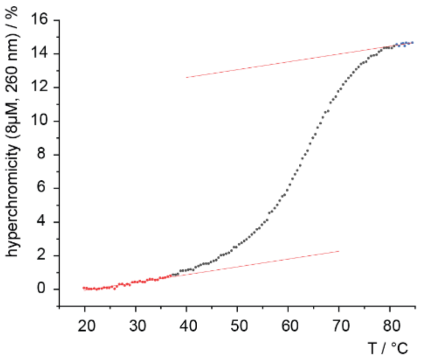

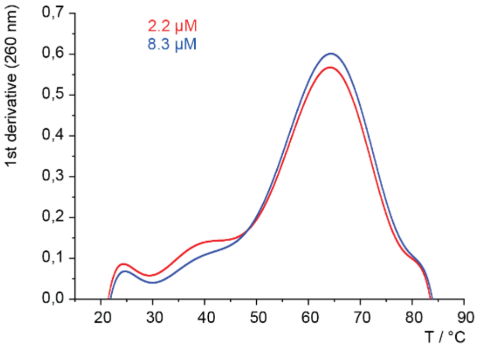

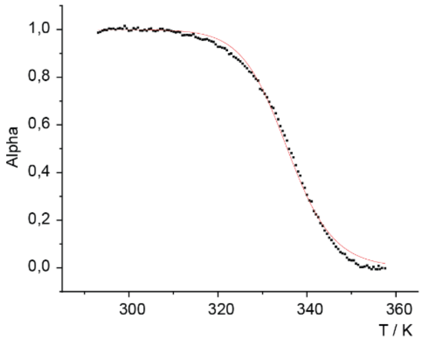

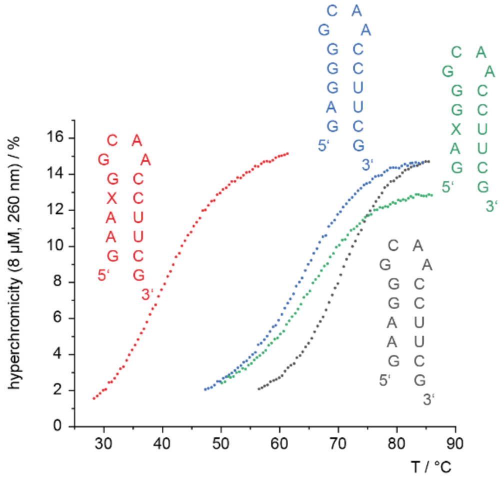

Supporting Figure 16. UV-melting profile analysis of RNA hairpin **IIId** at pH 7; and overview of melting profiles of **IIIa-d** (bottom right).

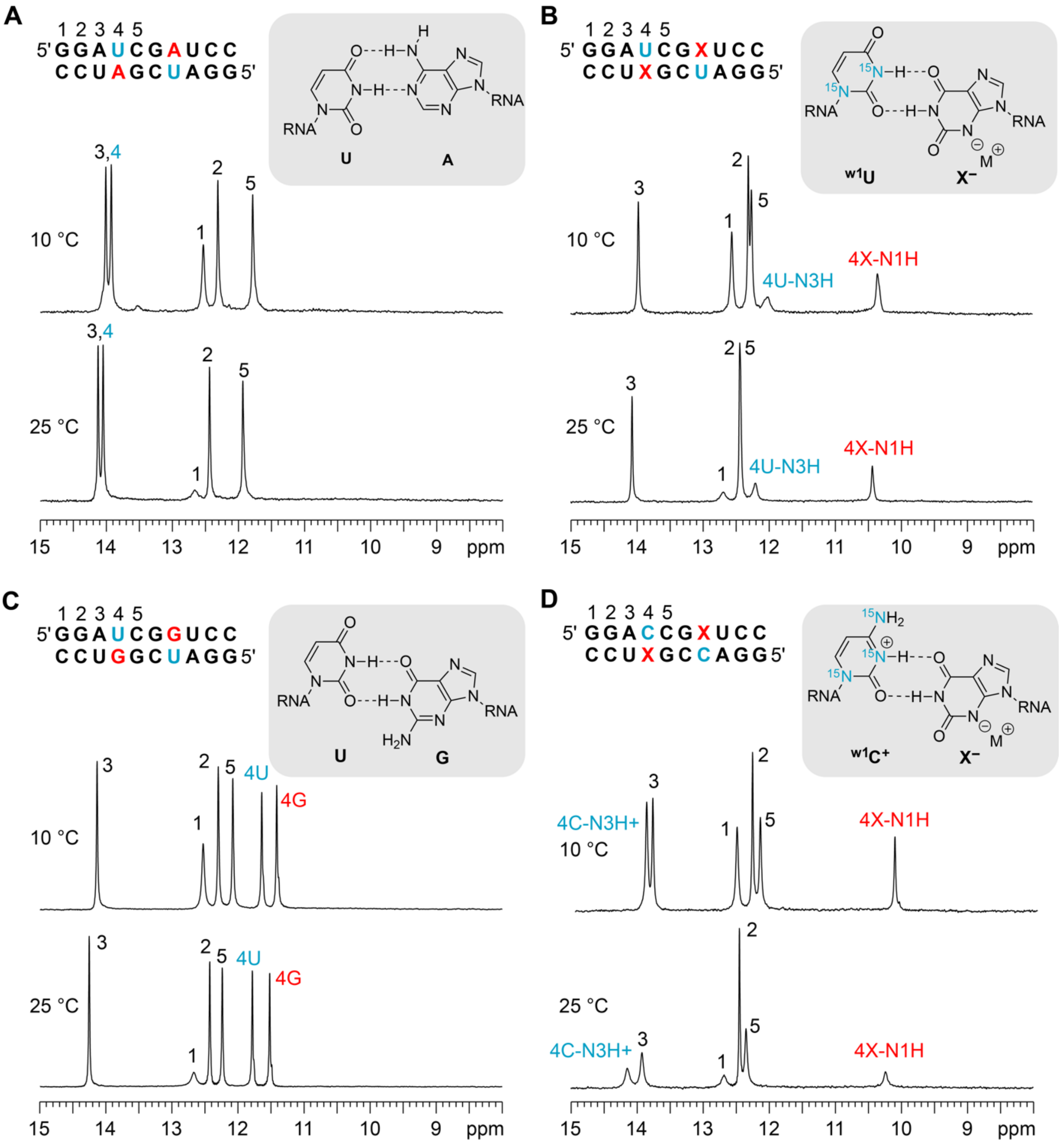

Supporting Figures S17. ^{1}H NMR imino proton spectra of 10 nt RNA palindromes **IIb**, **IId**, **IIe**, and **IIc**, with (**A**) U-A, (**B**) U-X, (**C**) U-G, and (**D**) C-X base pairs in position 4-7' and 4'-7. Conditions: c(RNA) = 0.1 mM, 25 mM NaCl, 15 mM sodium phosphate buffer, 10% D_2O, pH 6.5.

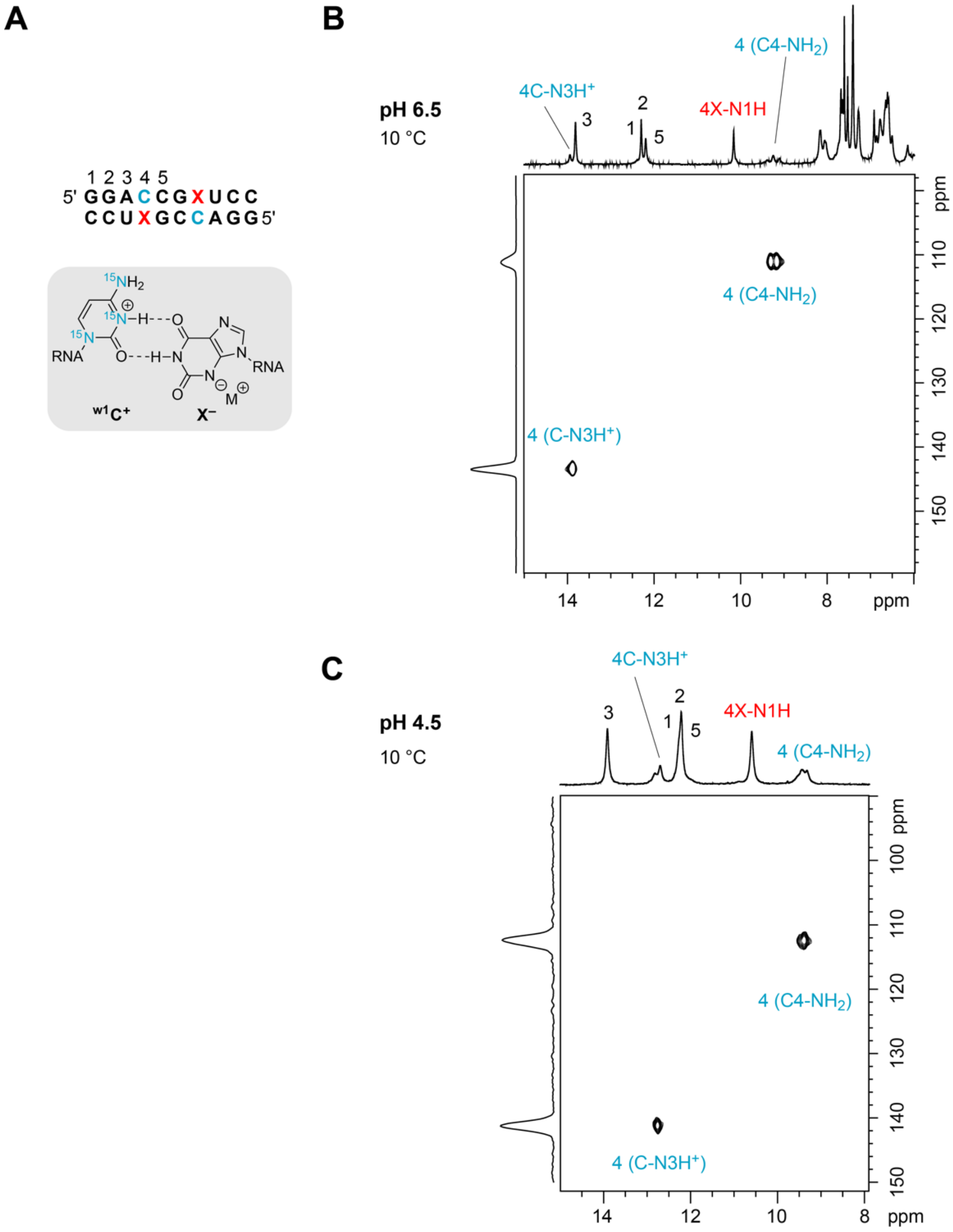

Supporting Figures S18. NMR spectroscopic analysis of the 10 nt RNA palindrome **IIc**. (**A**) RNA sequence and chemical structure C-X wobble pairs in position 4-7' and 4'-7. (**B**) $^{1}H,^{15}N$-HSQC NMR spectrum at pH 6.5. (**C**) ^{1}H,15N-HSQC NMR spectrum at pH 4.5. Conditions: c(RNA) = 1.0 mM, 25 mM NaCl, 15 mM sodium phosphate buffer, 10% D_2O, pH and temperature as indicated.

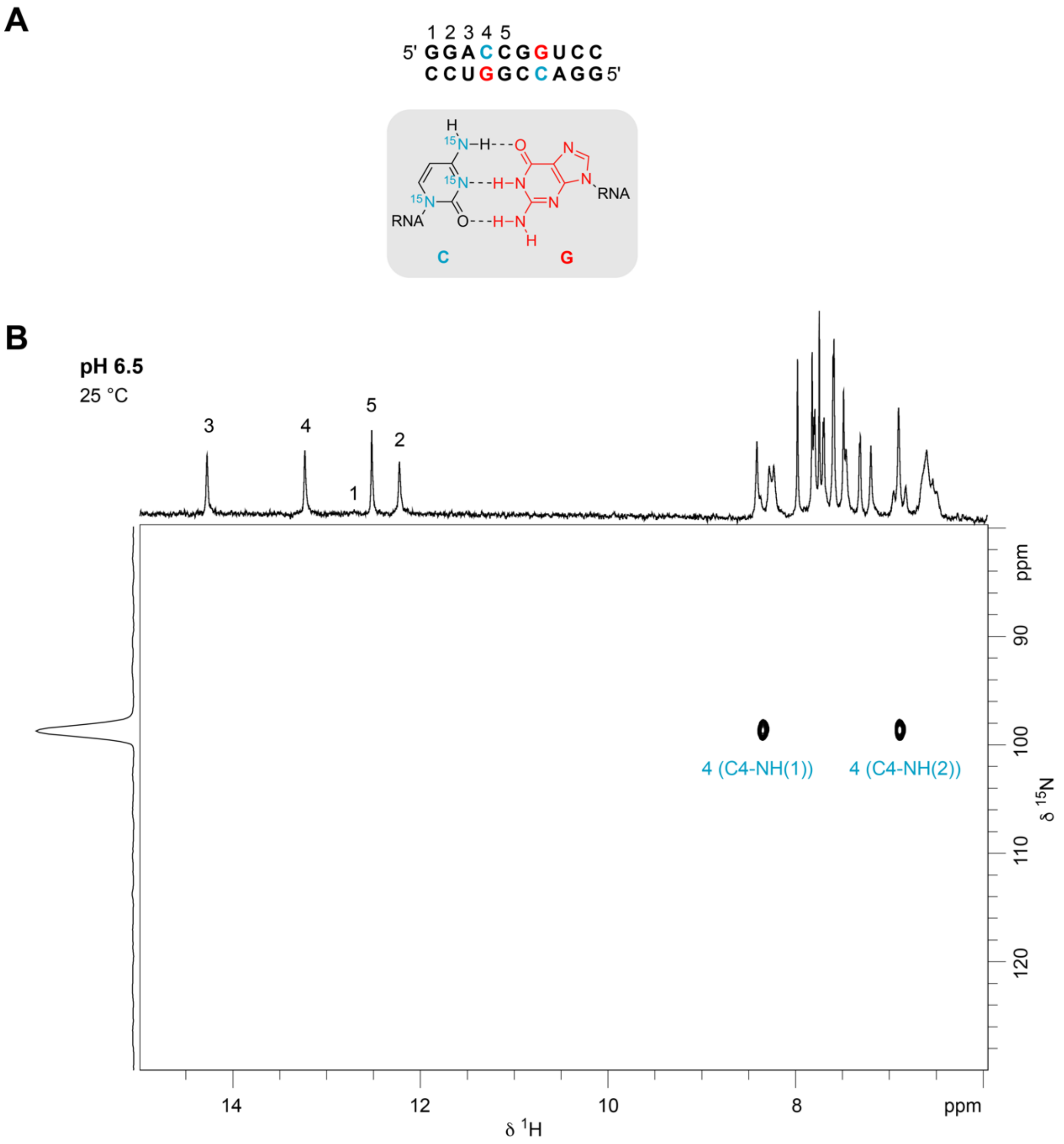

Supporting Figures S19. NMR spectroscopic analysis of the 10 nt RNA palindrome **IIa**. (**A**) RNA sequence and chemical structure C-G pairs in position 4-7' and 4'-7. (**B**) ^{1}H,^{15}N-HSQC NMR spectrum at pH 6.5. c(RNA) = 0.2 mM, 25 mM NaCl, 15 mM sodium phosphate buffer, 10% D_2O, temperature as indicated.

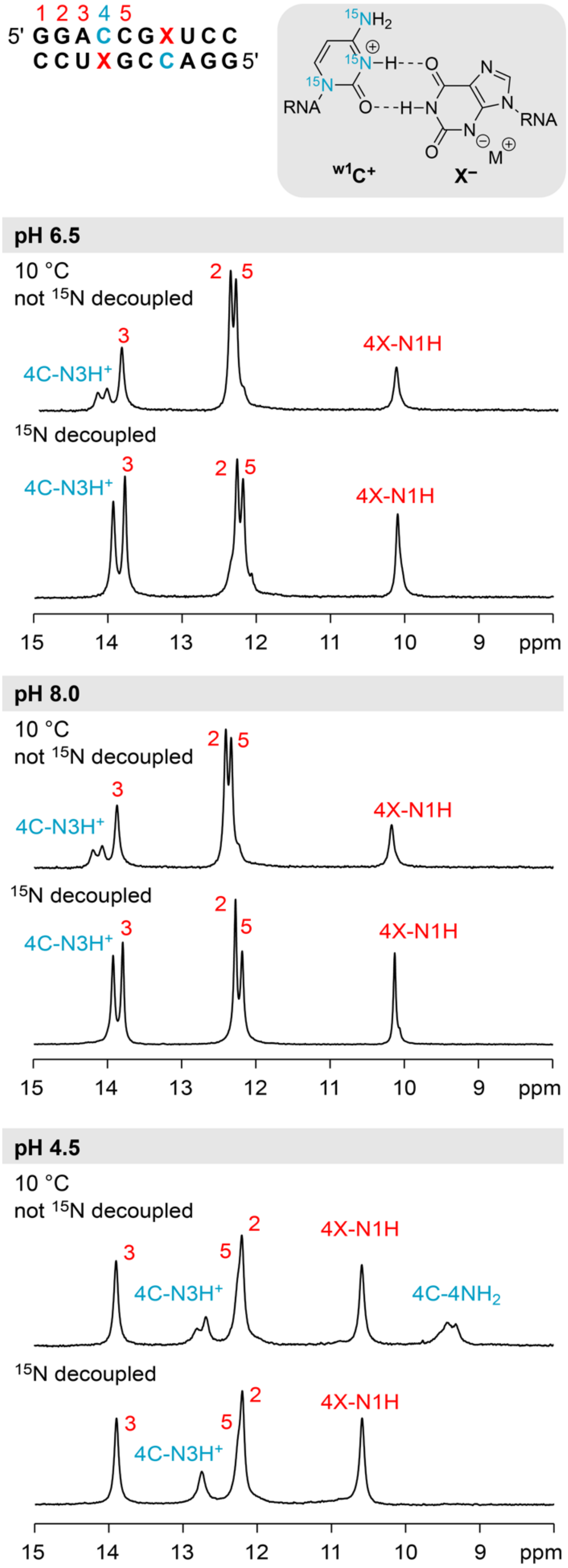

Supporting Figures S20. NMR spectroscopic analysis of the 10 nt RNA palindrome **IIc**. RNA sequence and chemical structure C-X pairs in position 4-7' and 4'-7. ^{1}H-NMR spectra at 10°C. c(RNA) = 1.0 mM, 25 mM NaCl, 15 mM sodium phosphate buffer, 10% D_2O, pH as indicated.

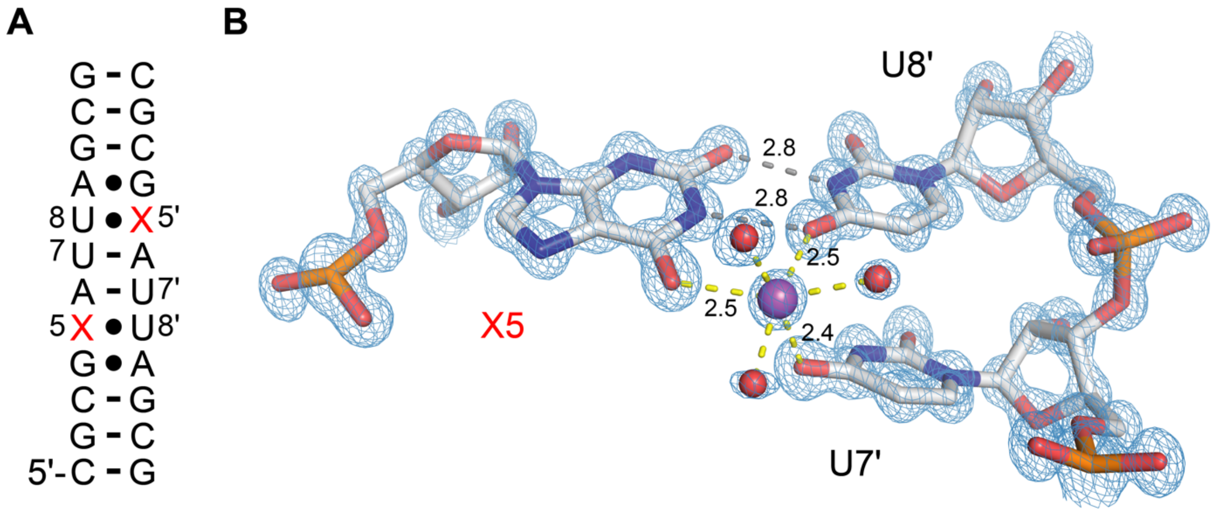

Supporting Figures S21. Crystal structure of a X-U modified 12 nt RNA palindrome. (**A**) RNA sequence and secondary structure. (**B**) Side view on the X5/U8' base pair and U7' highlighting the sodium ion binding site (PDB ID 7QUA).

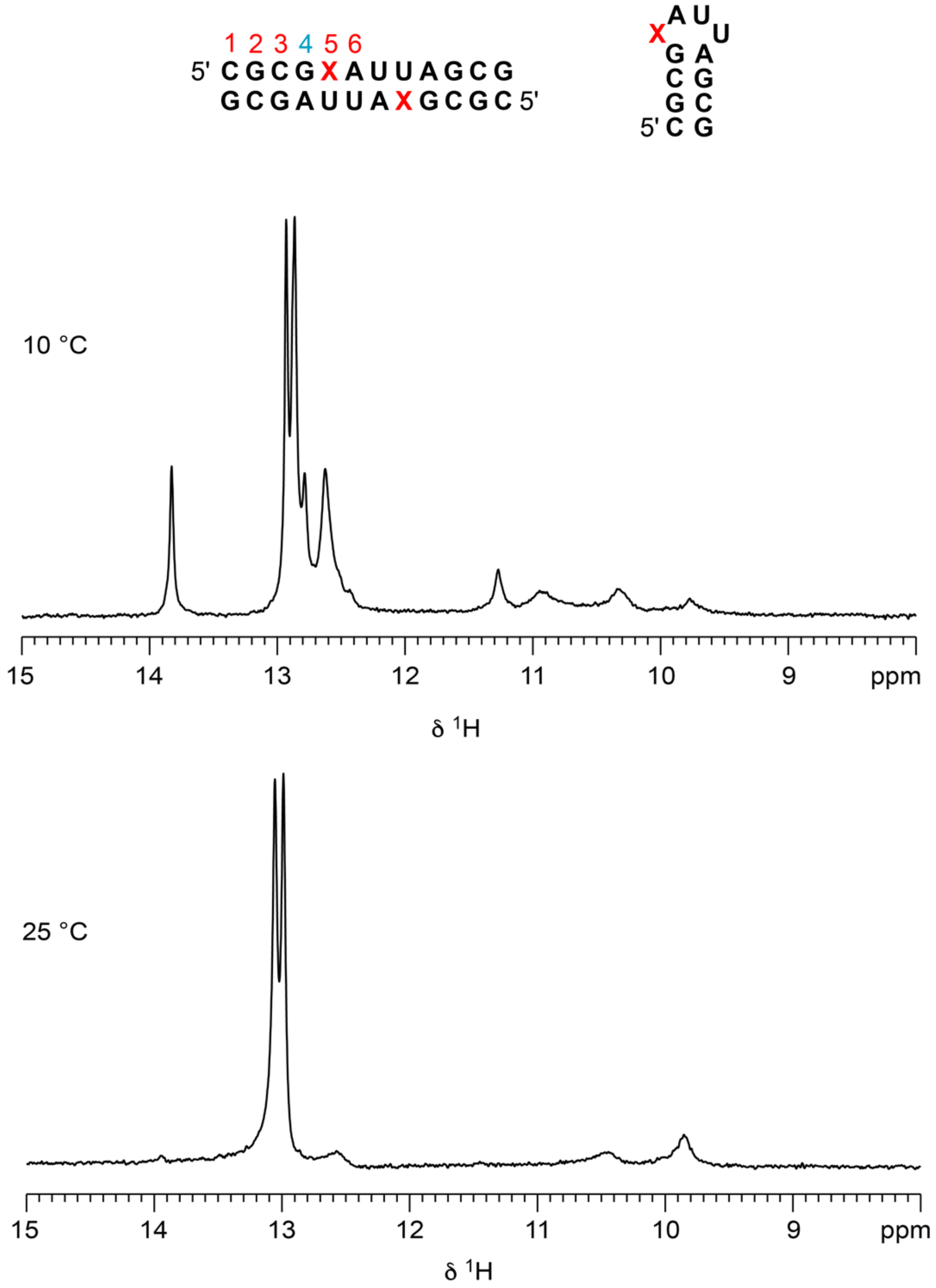

Supporting Figures S22. NMR spectroscopic analysis of the 12 nt RNA palindrome used in the X-ray study. RNA sequence and potential secondary structures. ^{1}H-NMR spectra at pH 6.5. c(RNA) = 0.1 mM, 25 mM NaCl, 15 mM sodium phosphate buffer, 10% D_2O, temperatures as indicated.

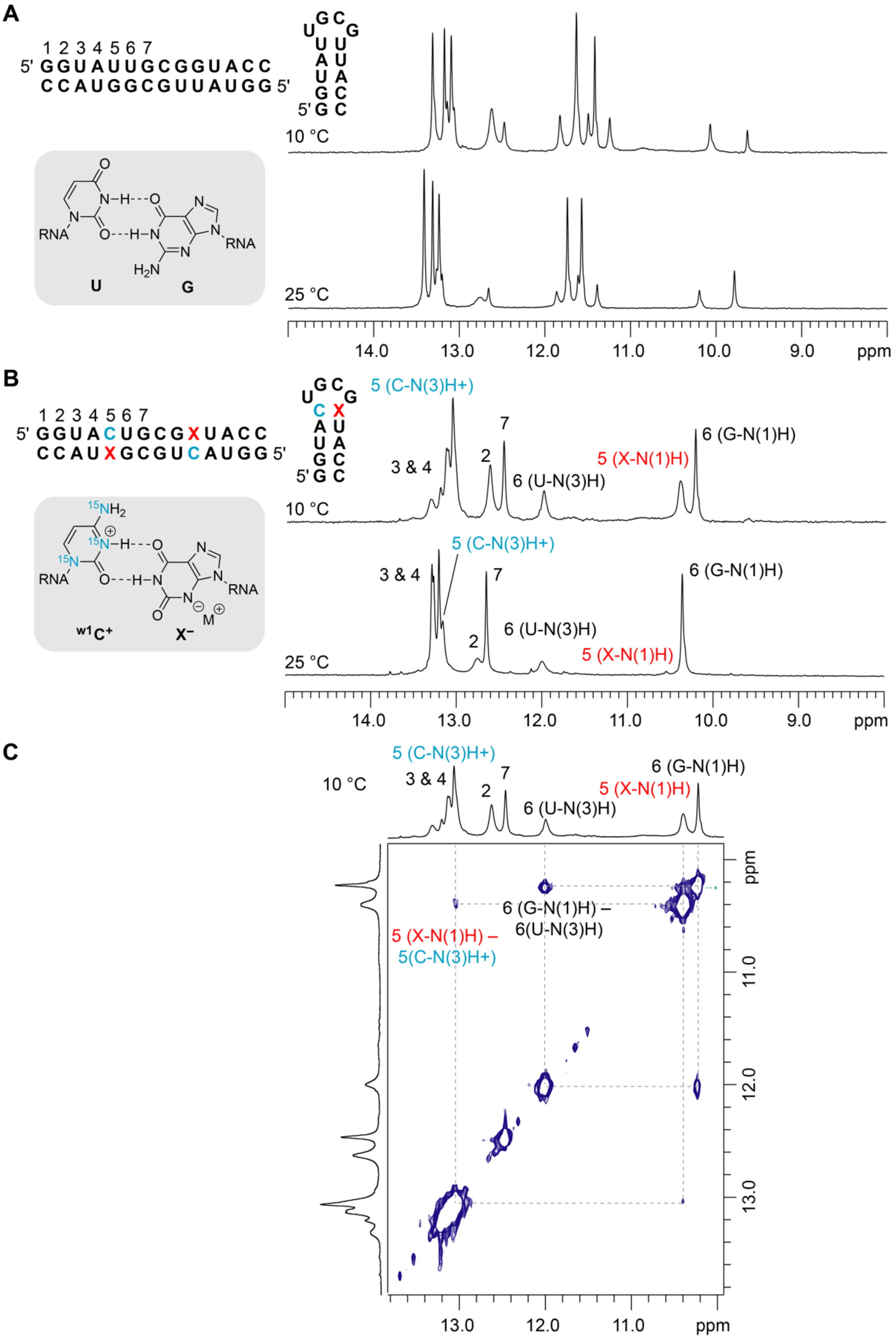

Supporting Figures S23. NMR spectroscopic analysis of the 14 nt RNA palindrome used in the X-ray study. (**A**) RNA sequence, potential secondary structures, and chemical structure U-G wobble pairs in position 5-10' and 5'-10; ^{1}H-NMR spectra at pH 6.5; temperatures as indicated. (**B**) RNA sequence, potential secondary structures, and chemical structure C-X wobble pairs in position 5-10' and 5'-10; ^{1}H-NMR spectra at pH 6.5; temperatures as indicated. (**C**) ^{1}H,^{15}N-HSQC NMR spectrum of the C-X modified RNA palindrome, at pH 6.5, 10°C. Conditions: c(RNA) = 0.1 mM, 25 mM NaCl, 15 mM sodium phosphate buffer, 10% D_2O.
